# Priority Micronutrient Density in Foods

**DOI:** 10.3389/fnut.2022.806566

**Published:** 2022-03-07

**Authors:** Ty Beal, Flaminia Ortenzi

**Affiliations:** ^1^Knowledge Leadership, Global Alliance for Improved Nutrition, Washington, DC, United States; ^2^Department of Environmental Science and Policy, University of California, Davis, Davis, CA, United States; ^3^Knowledge Leadership, Global Alliance for Improved Nutrition, Geneva, Switzerland

**Keywords:** nutrient density, micronutrient deficiencies, animal-source foods, organs, shellfish, fish, dark green leafy vegetables, ruminant meat

## Abstract

**Background:**

Despite concerted efforts to improve diet quality and reduce malnutrition, micronutrient deficiencies remain widespread globally, especially in low- and middle-income countries and among population groups with increased needs, where diets are often inadequate in iron, zinc, folate, vitamin A, calcium, and vitamin B_12_. There is a need to understand the density of these micronutrients and their bioavailability across diverse foods and the suitability of these foods to help meet requirements for populations with high burdens of micronutrient malnutrition.

**Objective:**

We aimed to identify the top food sources of these commonly lacking micronutrients, which are essential for optimal health, to support efforts to reduce micronutrient malnutrition among various populations globally.

**Methods:**

We built an aggregated global food composition database and calculated recommended nutrient intakes for five population groups with varying requirements. An approach was developed to rate foods according to their density in each and all priority micronutrients for various population groups with different nutrient requirements.

**Results:**

We find that the top sources of priority micronutrients are organs, small fish, dark green leafy vegetables, bivalves, crustaceans, goat, beef, eggs, milk, canned fish with bones, mutton, and lamb. Cheese, goat milk, and pork are also good sources, and to a lesser extent, yogurt, fresh fish, pulses, teff, and canned fish without bones.

**Conclusion:**

The results provide insight into which foods to prioritize to fill common micronutrient gaps and reduce undernutrition.

## Introduction

Food is integral to everyday life, providing essential energy and nutrients for human function. An important aspect of food, among others, is the vitamins and minerals it provides. Yet in many low- and middle-income countries (LMICs) diets are known to be lacking in micronutrients, especially for population groups with increased needs, leading to deficiencies, particularly in iron, zinc, folate, vitamin A, calcium, and vitamin B_12_ (hereafter referred to as “priority micronutrients”), that can have severe and lasting effects (1–6). For example, more than four in five Indian adolescents have a deficiency in one or more micronutrients (7). Even in high-income countries (HICs) like the United States and United Kingdom, micronutrient deficiencies such as iron deficiency are often common, especially among women of reproductive age (WRA) (3, 8). Globally, current diets are failing to provide adequate density of these essential micronutrients. Furthermore, anthropogenic CO_2_ emissions are reducing iron and zinc concentrations in crops, which highlights the increasing importance of improving dietary nutrient density (9).

There is an urgent need, therefore, to increase the density of priority micronutrients in diets in countries of all incomes. One efficient and cost-effective strategy for reducing micronutrient deficiencies is food fortification (10). However, there are more than 70,000 compounds in foods (11) bound together in a food matrix, which synergistically impact metabolism, including nutrient absorption, and may have beneficial effects on satiety and the immune system, offering protection from disease, among other potentially important health implications (12–15). Thus, fortifying staple foods with priority micronutrients is important but does not fully replicate inherently nutrient-dense foods and their health effects. Obtaining adequate micronutrients from minimally processed foods may have additional benefits beyond fortification due to the added value of diverse synergistic nutrients within a food matrix (12–14). Moreover, while there is large variation in the health effects of different foods and dietary patterns, energy-dense ultra-processed foods (UPFs) in particular are associated with numerous non-communicable diseases (NCDs) and mortality (16). UPFs make up a large share of calories in most high-income countries and are increasing rapidly in most LMICs (16). Energy-dense ultra-processed foods are generally hyper palatable which can lead to overconsumption and weight gain when they are a predominant component of the food environment (17). Improving overall diet quality, especially the quantity and diversity of minimally processed foods inherently dense in priority micronutrients is crucial to reduce micronutrient malnutrition while minimizing the transition to UPFs and potential associated increase in NCDs.

Our study aims to identify the top food sources of commonly lacking micronutrients, which are essential for optimal health, to support efforts to reduce micronutrient malnutrition among various populations globally, particularly in low- and middle-income countries.

## Materials and Methods

### Recommended Nutrient Intakes

We calculated recommended intakes for adults ≥25 years of age and groups vulnerable to undernutrition, including children 2–4 years, adolescents, non-pregnant and non-lactating WRA, and pregnant women, from the European Food Safety Authority (EFSA) (18) for vitamin A, folate, calcium, and zinc and from the Institute of Medicine (19) for vitamin B_12_ and iron. This aligns with the recently proposed harmonized nutrient reference values (20), except for iron, because EFSA values are based on the assumption that the population has iron stores, which is not the case for many people in LMICs. We used recommended nutrient intakes rather than average requirements because we are interested in target values for individuals, not in estimating population level adequacy.

### Building a Global Food Composition Database

We built a global food composition database (excluding fortified foods), with values for calories, phytate (21), and six priority micronutrients: vitamin A, folate, vitamin B_12_, calcium, iron, and zinc. Nutrient densities are from USDA FoodData Central (FDC) (22) and national and regional food composition tables (FCTs) from LMICs globally (23): Kenya, Malawi, and Western Africa (Sub-Saharan Africa); Bangladesh, Indonesia, Laos, Vietnam, and ASEAN (South and South-East Asia); Mexico and Colombia (Latin America). These FCTs contained values from analyzed foods. However, for teff, fonio, and small dried fish, we also included values from the literature due to limited availability in FCTs (see Supplementary Material for details).

Foods were aggregated when showing relatively low nutrient density variance (for example, pulses and yogurt) or when likely to be targeted as a food group in policy and programming (for example, DGLVs). Global nutrient values for individual foods were obtained by calculating medians of composite values from the selected FCTs. Composite values were obtained by averaging nutrient values for different cooking methods (and/or raw foods) and/or different cuts of a given food for meat. Global nutrient values for aggregated food groups were obtained by averaging composite values at the regional level and from FDC. Composite values for a given region were obtained by calculating the medians of nutrient values for several individual foods within a food group, available in the selected FCTs corresponding to that region. Standard deviations were calculated for all obtained global nutrient values, as a measure of variability across included FCTs.

We accounted for iron and zinc bioavailability. For iron, foods were classified into one of three levels of iron absorption (20% for ruminant meat, 15% for all other animal-source foods, and 10% for all plant-source foods), based on the proportion of heme to non-heme iron contained (1): 68% heme-iron in ruminant meat, including beef (24–26), goat, and lamb/mutton (26, 27); 39% heme-iron in pork (25, 26, 28–30); 26% heme-iron in chicken (25, 26, 28–30), fish and seafood (25, 28–31), and eggs and dairy (29); and 40% heme-iron in all other meat, including offal (24, 29, 30). Regarding zinc, foods were classified into one of four levels of zinc absorption (44, 35, 30, and 26%), based on the amount of phytate contained in each food in a portion equivalent to one-third of daily mass intake, assuming an energy density of 1.3 kcal/g and considering average requirements for energy for a moderately active WRA (18) (see Supplementary Material for details).

### Priority Micronutrient Density Rating

Foods were classified into one of four levels of micronutrient density based on the calories and grams needed to provide one-third (for individual nutrients) or an average of one-third (for the aggregate score) of recommended intakes of vitamin A, folate, vitamin B_12_, calcium, iron, and zinc. For the aggregate score, the average share of recommended intakes (*ASRI*) across the six micronutrients (*A*), for a given quantity of calories and grams (*i*), of a given food (*j*), was calculated as:


$$A\,S\,R\,I_{i,j}=\frac{1}{\left|A\right|}\,\sum_{a\in A}\text{min}\,\left\{\frac{n\,u\,t\,r\,i\,e\,n\,t\,\_\,d\,e\,n\,s\,i\,t\,y_{a,j}*i}{r\,e\,c\,o\,m\,m\,e\,n\,d\,e\,d\,\_\,i\,n\,t\,a\,k\,e\,s_{a}},1\right\}$$

A similar approach was previously used to identify micronutrient-dense complementary foods for young children (4, 32). Ratings were calculated for different population groups according to the following thresholds for Average Requirements (ARs) of energy for a moderately active individual (18) and hypothetical ARs for mass, assuming an energy density of 1.3 kcal/g [the mean energy density of a minimally processed plant-based, low-fat diet and animal-based, ketogenic diet (33)]:

• Very high: ≤one-sixth of ARs for both energy and mass.
• High: ≤one-third of ARs for both energy and mass and <one-sixth of ARs for either energy or mass.
• Moderate: ≤one-third and >one-sixth of ARs for both energy and mass.
• Low: >one-third of ARs for either energy or mass.

Micronutrient density of milk was classified based solely on ARs for energy, since mass is typically not a limiting factor for liquids. The same energy thresholds as for solid foods were used for very high and low micronutrient density. For high micronutrient density, thresholds were ≤ one-fourth and > one-sixth of ARs for energy. For moderate micronutrient density, thresholds were ≤ one-third and > one-fourth of ARs for energy.

As indicated in the formula for the aggregate score, each micronutrient’s contribution was capped at 100% of recommended intakes, which means that each micronutrient can contribute nothing or up to one-half of the total score (4, 32). To illustrate this, a food containing only two of the six nutrients would provide 100% of recommended intakes of both nutrients, while a food with a perfectly even proportion of recommended intakes across all six nutrients would provide 33.3% of recommended intakes of all six nutrients—each micronutrient thus contributing an equal one-sixth of the total score. This approach ensures that for foods to rate high, they need to be high in at least two micronutrients and that foods with very high densities of individual micronutrients are not rated higher for providing amounts well above recommended intakes or above upper limits.

## Results

### Recommended Nutrient Intakes

Recommended nutrient intakes vary by population and, for iron and zinc, bioavailability (Table 1). Among groups with roughly similar ARs for energy, recommended nutrient intakes are generally highest for pregnant women, followed by adults, WRA, and adolescents, but there is variability by nutrient. Notably, recommended folate intake for pregnant women is double than for adults, WRA, and adolescents; recommended iron intake for pregnant women is more than triple than for adults, more than double than for adolescents, and more than 50% higher than for WRA. Recommended intakes for vitamin A, vitamin B_12_, calcium, and zinc vary less across these groups.

**TABLE 1:** Recommended nutrient intakes for select groups.

Group	AER (kcal)	Vit A (mcg RAE)	Folate (mcg DFE)	Vit B_12_ (mcg)	Calcium (mg)	Iron (mg)^1^			Zinc (mg)^2^			
	
							20%	15%	10%	R	SR	SU	U
Children 2–4	1,246	267	128	1.0	590	7.4	9.8	14.8	3.2	3.9	4.7	5.5
Adolescents 10–19	2,296	630	292	2.2	1,085	9.9	13.2	19.8	8.3	9.9	11.4	13.0
Women 15–49	2,305	650	325	2.4	977	15.9	21.2	31.8	8.0	9.6	11.1	12.6
Pregnant women 15–49	2,583	700	600	2.6	977	24.3	32.4	48.6	9.1	10.9	12.6	14.3
Adults 25+^3^	2,227	700	328	2.4	950	9.4	12.8	18.7	8.5	10.5	12.5	14.5

Average energy requirements for a moderately active individual and recommended intakes for vitamin A, folate, calcium and zinc from the European Food Safety Authority (18). Recommended intakes for iron and vitamin B_12_ from the Institute of Medicine (19).

^1^Percentages represent different levels of bioavailability that correspond with the possible classifications of each food in the analysis.

^2^Assuming 300 mg phytate/day and 44% absorption for refined (R) diets, 600 mg phytate/day and 35% absorption for semi-refined (SR) diets, 900 mg phytate/day and 30% absorption for semi-unrefined (SU) diets, and 1,200 mg phytate/day and 26% absorption for unrefined (U) diets.

^3^Includes both men and women. AER, Average Energy Requirement; DFE, dietary folate equivalent; R, refined; RAE, retinol activity equivalent; SR, semi-refined; SU, semi-unrefined; U, unrefined; Vit, vitamin.

### Global Food Composition Database

Table 2 shows the compiled global food composition database of 41 individual and aggregate foods, with values for the six priority micronutrients, energy, phytate, and iron and zinc bioavailability (a version of the global food composition database which includes standard deviations is available in Supplementary Table 1). Interestingly, some food groups showed high nutrient density variance across included foods, such as DGLVs, with spinach, amaranth leaves, and cassava leaves having much higher values than lettuce and cabbage (Supplementary Table 4). Similarly, hard cheese (for example, cheddar and aged goat cheese) and fatty fish (for example, herring and mackerel) were more nutrient-dense than soft cheese (for example, cottage cheese) and lean fish (for example, cod and tilapia), respectively (Supplementary Table 6). Other food groups, such as pulses, presented more equal nutrient density distributions across foods, but there were significant differences across FCTs (Supplementary Tables 2–7). For instance, Sub-Saharan Africa and South/South-East Asia showed much lower values for folate in pulses than Latin America and FDC, which may be due to different varieties, culinary traditions, and cooking methods and times.

**TABLE 2:** Global food composition database.

Food (100 g)	kcal	Vit A (mcg RAE)	Folate (mcg DFE)	Vit B_12_ (mcg)	Calcium (mg)	Iron (mg)	Zinc (mg)	Iron Abs	Zinc Abs	Phytate (mg)
Pulses	134	1	88	0	29	2.4	1.2	0.10	0.26	441
Whole grains^1,9^	204	0	16	0	22	1.9	1.5	0.10	0.26	510
Refined grains^9^	133	0	5	0	9	0.5	0.6	0.10	0.44	45
Unrefined grain products^2,9^	166	2	31	0	29	1.9	1.0	0.10	0.30	129
Refined grain products^9^	168	0	12	0	12	0.8	0.5	0.10	0.44	49
Sorghum^3^	142	0	20	0	9	2.6	0.8	0.10	0.26	272
Millet	148	0	27	0	10	2.6	1.0	0.10	0.26	200
Teff^4,5^	149	0	42	0	49	4.3	1.1	0.10	0.26	284
Fonio^3,4^	139	1	36	0	12	2.8	1.1	0.10	0.30	110
Quinoa^6^	115	0	43	0	17	2.0	1.0	0.10	0.26	554
Roots, tubers, plantains	111	14	12	0	17	0.7	0.3	0.10	0.44	13
Nuts	594	0	72	0	62	4.1	3.0	0.10	0.26	670
Seeds	579	1	98	0	333	7.6	5.5	0.10	0.26	653
Dark green leafy vegetables	30	252	57	0	148	2.2	0.4	0.10	0.44	17
Vitamin A-rich fruits/vegetables	40	124	24	0	20	0.5	0.2	0.10	0.44	24
Other vegetables	28	20	17	0	18	0.5	0.2	0.10	0.44	10
Other fruits	65	4	19	0	11	0.4	0.2	0.10	0.44	10
Eggs	156	163	45	1.1	50	1.6	1.1	0.15	0.44	0
Fresh cow milk	67	44	5	0.4	120	0.1	0.4	0.15	0.44	0
Cooked cow milk	61	39	5	0.5	116	0.1	0.5	0.15	0.44	0
Fresh goat milk	72	35	1	0.1	143	0.1	0.3	0.15	0.44	0
Yogurt	77	27	7	0.4	121	0.1	0.4	0.15	0.44	0
Cheese	359	213	16	1.0	707	0.5	3.0	0.15	0.44	0
Beef	217	3	4	2.1	8	2.5	5.8	0.20	0.44	0
Goat	146	0	3	1.2	17	3.2	5.0	0.20	0.44	0
Lamb/mutton	290	0	9	2.5	14	2.0	4.7	0.20	0.44	0
Pork	242	2	3	0.7	23	1.6	2.6	0.15	0.44	0
Chicken	229	29	6	0.3	14	1.0	1.5	0.15	0.44	0
Beef liver	177	8645	257	76.2	7	9.0	5.2	0.15	0.44	0
Goat/lamb liver^4^	207	18093	349	85.6	11	10.0	6.3	0.15	0.44	0
Chicken liver	141	3492	534	16.1	10	10.3	3.4	0.15	0.44	0
Pork liver^7^	114	4796	148	15.8	10	17.9	4.6	0.15	0.44	0
Heart^7^	112	4	25	5.1	7	4.4	3.1	0.15	0.44	0
Spleen^6^	114	0	3	4.2	10	35.8	2.5	0.15	0.44	0
Kidney	97	69	34	14.5	12	5.6	2.4	0.15	0.44	0
Fresh fish^10^	123	10	10	1.8	39	0.8	0.7	0.15	0.44	0
Small dried fish^4^	294	186	37	12.1	2360	10.0	10.2	0.15	0.44	0
Canned fish, without bones^8^	153	14	5	2.2	12	1.3	0.6	0.15	0.44	0
Canned fish, with bones^8^	201	40	9	5.8	252	2.3	1.4	0.15	0.44	0
Crustaceans	89	8	12	1.3	74	1.2	2.2	0.15	0.44	0
Bivalves	87	77	19	23.5	113	4.8	3.6	0.15	0.44	0

*^1^Only one food item available for the South and South East Asia.*

*^2^No foods available for the South and South East Asia.*

*^3^No values available from FoodData Central.*

*^4^Values available for Sub-Saharan African only. Additional values from the literature used.*

*^5^For zinc absorption, used average phytate of the four other traditional grains (sorghum, millet, fonio, and quinoa).*

*^6^Values available for Latin America only.*

*^7^Values available for South and South East Asia and Latin American only.*

*^8^Values available for Sub-Saharan Africa and Latin America only.*

*^9^The term “grains” (both whole and refined) refers to cereal grains, such as wheat, rice, oats, and barley. The term “grain products” (both unrefined and refined) refers to products obtained from flours, requiring some additional processing, such as breads, pasta, and noodles.*

*^10^Aggregate group including different species of marine and freshwater fish. Abs, absorption; Vit, vitamin.*

### Aggregate Micronutrient Density Scores for Women of Reproductive Age

**FIGURE 1:**
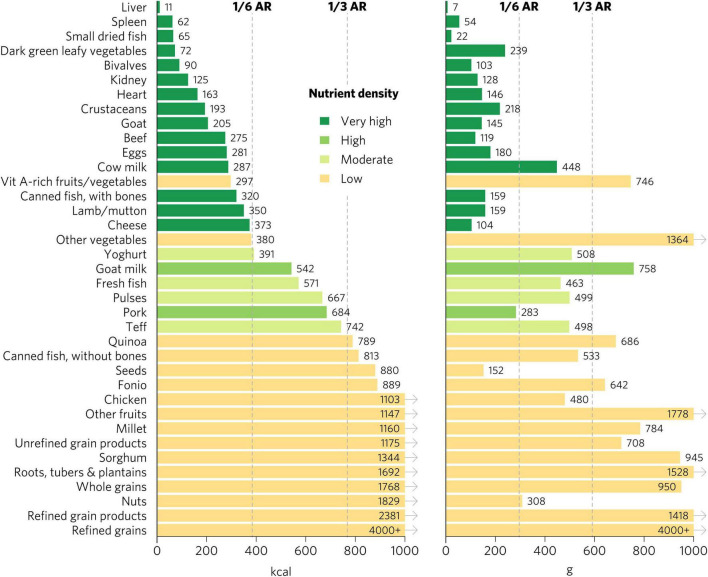
Calories and grams needed to provide an average of one-third of recommended intakes of vitamin A, folate, vitamin B_12_, calcium, iron, and zinc for women of reproductive age. Each micronutrient’s contribution is capped at 100% of recommended intakes. Hypothetical average requirements for mass are based on an energy density of 1.3 kcal/g. AR, average requirement; Vit, vitamin.

We emphasize the results for WRA in the main text because they are the largest population group, >1.8 billion globally, that is at increased risk for micronutrient malnutrition. The quantity of calories and grams required to provide an average of one-third of recommended intakes for WRA of vitamin A, folate, vitamin B_12_, calcium, iron, and zinc varies widely by food (Figure 1). Foods with very high aggregate micronutrient density for WRA include organs (liver, spleen, kidney, and heart from beef, goat, lamb, chicken, and pork), small dried fish, DGLVs, bivalves (clams, mussels, and oysters), crustaceans, goat, beef, eggs, milk, canned fish with bones, lamb/mutton, and cheese. Foods with a high aggregate micronutrient density include goat milk and pork. Foods with a moderate aggregate micronutrient density include yogurt, fresh fish (including different species of marine and freshwater fish), pulses, and teff. All other foods included in the analysis scored as having low aggregate micronutrient density for WRA.

### Individual Micronutrient Density Scores for Women of Reproductive Age

**FIGURE 2:**
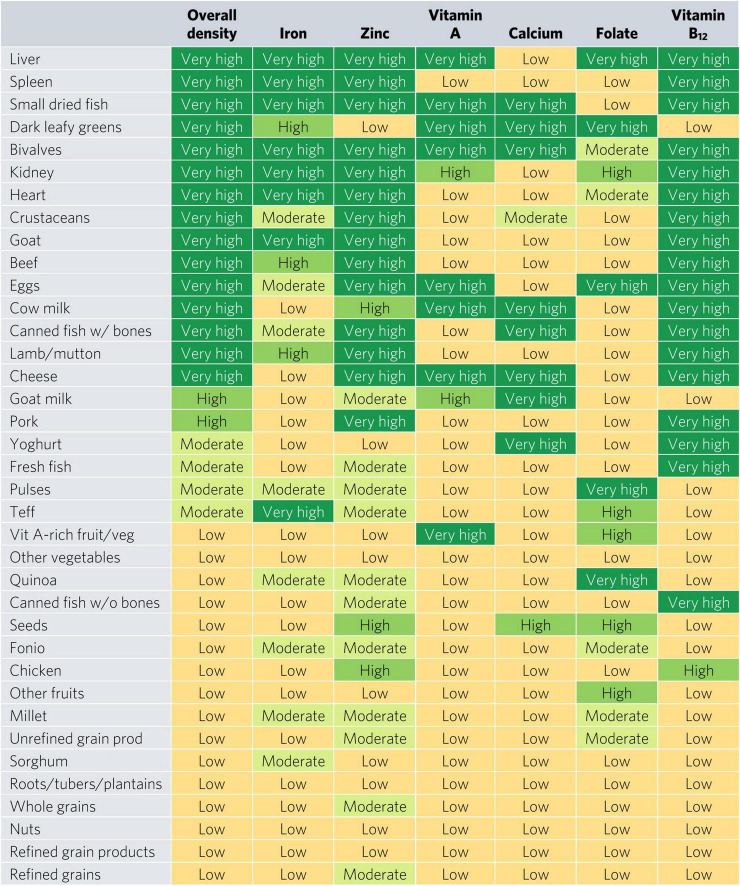
Aggregate and individual micronutrient density scores for women of reproductive age. prod, products; veg, vegetables.

Bivalves are the only food to contain at least a moderate density of all six micronutrients for WRA—they contained a very high density (hereafter referred to as “top sources”) of all micronutrients except for folate, for which they contain a moderate density (Figure 2). Most animal-source foods and DGLVs were top sources of two or more micronutrients. All foods contained at least a moderate density of at least one of the six micronutrients except for other vegetables; roots, tubers, and plantains; nuts; and refined grain products.

**FIGURE 3:**
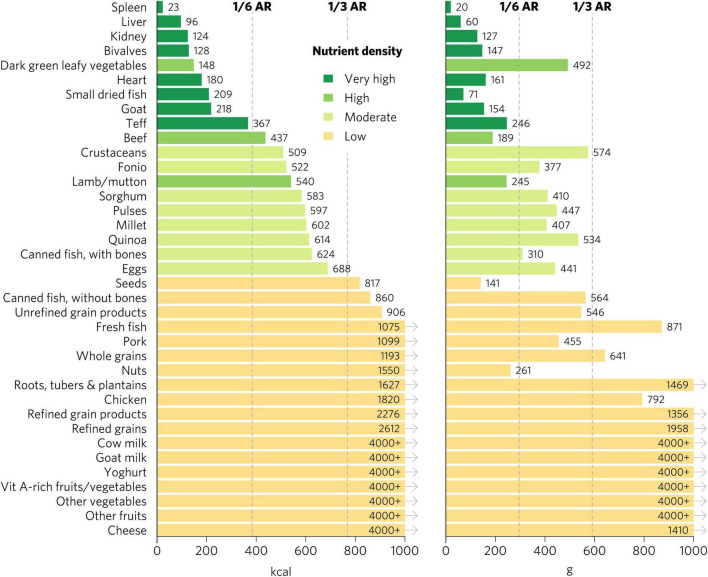
Calories and grams needed to provide one-third of recommended iron intakes for women of reproductive age. Hypothetical average requirements for mass are based on an energy density of 1.3 kcal/g. AR, average requirement; Vit, vitamin.

Top iron sources included organs, bivalves, small dried fish, goat, and teff, each providing one-third of recommended iron intakes with less than one-sixth of ARs for energy and hypothetical ARs for mass (Figures 2, 3). Top zinc sources included organs, bivalves, crustaceans, goat, beef, eggs, canned fish with bones, lamb/mutton, cheese, and pork (Figure 2). Top vitamin A sources included liver (including beef, goat, lamb, chicken, and pork liver), small dried fish, DGLVs, bivalves, eggs, cow milk, cheese, and vitamin A-rich fruits and vegetables. Top calcium sources included small dried fish, DGLVs, bivalves, cow milk, canned fish with bones, cheese, goat milk, and yogurt. Top folate sources included liver, DGLVs, eggs, pulses, and quinoa. Finally, top vitamin B_12_ sources included organs, small dried fish, bivalves, crustaceans, ruminant meat, eggs, milk, cheese, canned fish, pork, yogurt, and fresh fish.

### Aggregate Micronutrient Density Scores for Other Population Groups

**FIGURE 4:**
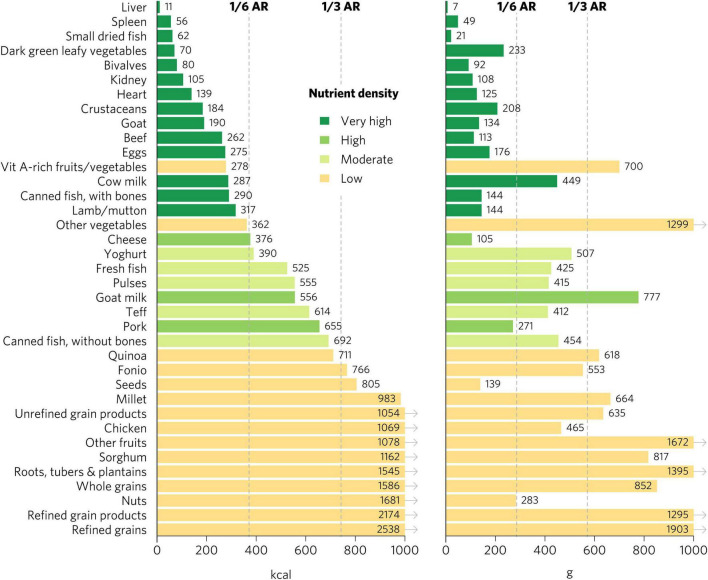
Calories and grams needed to provide an average of one-third of recommended intakes of vitamin A, folate, vitamin B_12_, calcium, iron, and zinc for adults ≥25. Each nutrient’s contribution is capped at 100% of recommended intakes. Hypothetical average requirements for mass are based on an energy density of 1.3 kcal/g. AR, average requirement; Vit, vitamin.

**FIGURE 5:**
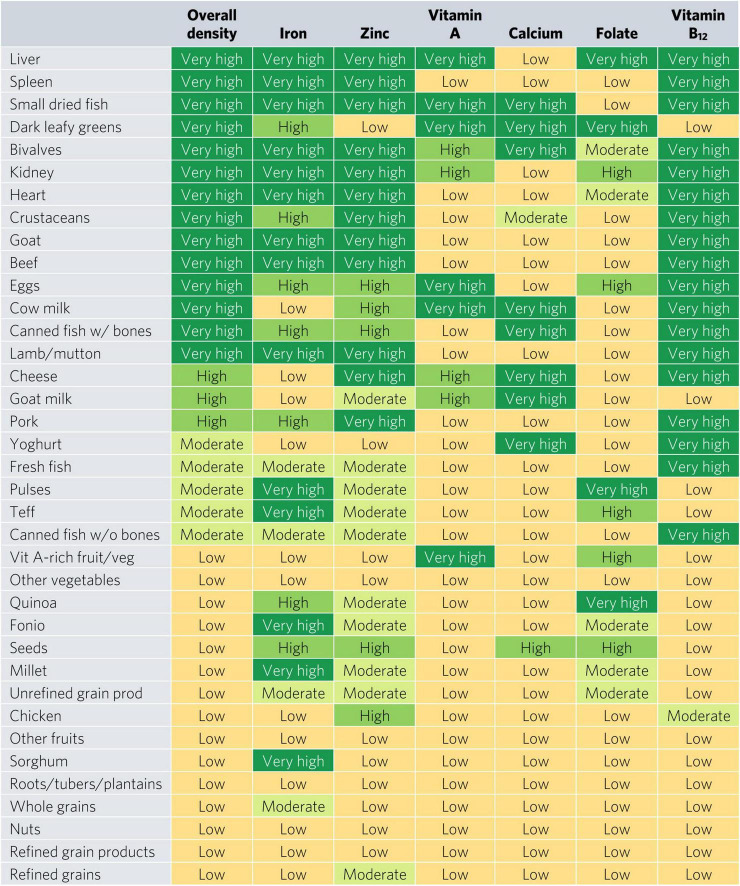
Aggregate and individual micronutrient density scores for adults ≥25. Mod, moderate; prod, products; veg, vegetables.

Micronutrient density scores may vary depending on the population, given differences in recommended nutrient intakes. The aggregate micronutrient density ratings remained similar for all other groups, with a few exceptions (Figures 4, 5 and Supplementary Figures 1–6). Organs, small dried fish, DGLVs, shellfish, beef, goat, eggs, cow milk, canned fish with bones, and lamb/mutton all remained with a rating of very high aggregate micronutrient density. Cheese rated very high for children 2–4 years, adolescents, WRA, and pregnant women but high for adults. Notably, vitamin A-rich fruits and vegetables and seeds rated high for children 2–4 years but low for all other groups. Canned fish without bones rated moderate for children 2–4 years, adolescents, and adults but low for WRA and pregnant women. Quinoa rated moderate for children 2–4 years and adolescents but low for all other groups. Finally, teff rated low for pregnant women but moderate for all other groups.

### Individual Micronutrient Density Scores for Other Population Groups

**FIGURE 6:**
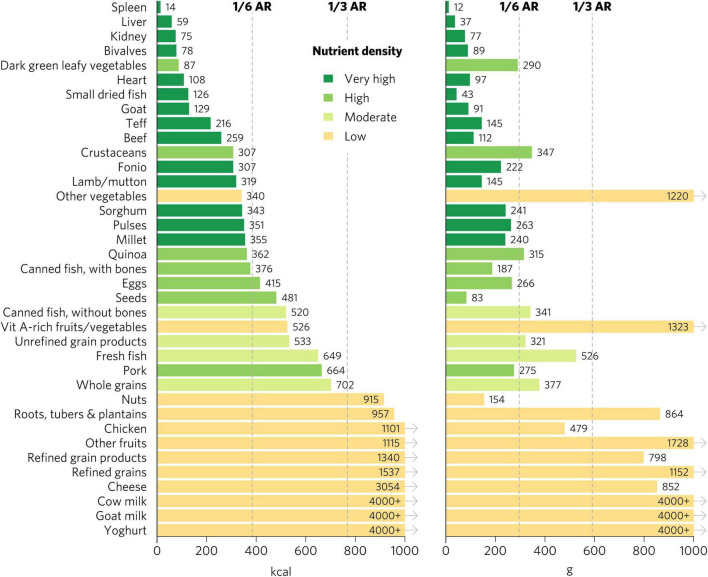
Calories and grams needed to provide one-third of recommended iron intakes for adults ≥25. Hypothetical average requirements for mass are based on an energy density of 1.3 kcal/g. AR, average requirement; Vit, vitamin.

There were many differences in ratings for specific micronutrients depending on the population, especially for iron and folate (Figures 5, 6 and Supplementary Figures 4–6). Organs, bivalves, small dried fish, and goat were the only foods that rated as top iron sources for all population groups. For iron, DGLVs rated low for pregnant women but high for all other groups, while crustaceans rated low for pregnant women, moderate for children 2–4 years and WRA, and high for adolescents and adults. Beef was a top source of iron for children 2–4 years, adolescents, and adults but rated high for WRA and pregnant women. For adults, teff, fonio, sorghum, pulses, and millet were all top iron sources, whereas they all rated low for pregnant women, except for teff and fonio, which rated moderate. Further, quinoa, canned fish with bones, eggs, seeds, and pork also rated high for iron for adults, while they rated moderate (quinoa, canned fish with bones, and eggs) or low (seeds and pork) for WRA and low for pregnant women. In addition, several food groups presented moderate iron density for adults, including fresh fish, canned fish without bones, whole grains, and unrefined grain products, whereas they all rated low for both WRA and pregnant women. Finally, for pregnant women, the only top folate sources were liver and pulses, whereas for adults and WRA top sources also included DGLVs and quinoa, with the addition of eggs for WRA and kidney (including beef, lamb, and pork kidney), fonio, and teff for children 2–4 years and adolescents.

## Discussion

Our analysis has provided ratings of inherent food sources of multiple and individual micronutrients commonly lacking in diets, especially in LMICs, for population groups with increased needs and the broader adult population. In general, animal-source foods and DGLVs are top sources of priority micronutrients. Interestingly, many foods commonly promoted as nutrient-dense, including most fruits and vegetables, nuts and seeds, whole grains and, even animal-source foods like chicken and canned fish without bones, are not particularly dense in bioavailable micronutrients commonly lacking in LMICs. These foods, of course, provide important nutritional benefits beyond these specific nutrients. Indeed, priority micronutrients are just one of many important aspects contributing to overall diet quality, and foods presenting low density in priority micronutrients may be rich in other essential and non-essential beneficial compounds and can contribute to overall energy and protein requirements.

These findings have implications for how to address important micronutrient gaps in the general population through food-based interventions. They are particularly relevant for populations with increased nutrient needs, such as pregnant women and WRA. We show that pregnant women and WRA need particularly nutrient dense foods to meet requirements and our analysis helps identify foods to prioritize. Programs and policies seeking to address undernutrition through dietary interventions in the most vulnerable populations could be improved by promoting specific foods containing the highest densities in bioavailable micronutrients commonly lacking, such as organs, small dried fish, DGLVs, and bivalves. For instance, policy makers and program managers could incentivize production of these foods to increase their availability and affordability, as well as adopt social protection policies and provide cash transfers to low-income households to purchase nutrient-dense foods. In addition, they could establish nutrition education programs within schools and the public health system and implement social and behavior change communication campaigns through mass media, to sensitize the general population or targeted groups on the importance of regularly consuming these foods within the context of a broader healthy diet. Similar policy and program implications have been identified in a related analysis that focused specifically on young children during the complementary feeding period in South and Southeast Asia and which found that organs, eggs, and bivalves had the highest densities of bioavailable micronutrients commonly lacking (34).

These findings also have important implications for vegetarian populations, since animal flesh foods are dense in priority micronutrients. In addition to DGLVs, both eggs and dairy foods are excellent sources of priority micronutrients for lacto-ovo vegetarians. Fortunately, eggs and dairy foods are among the more affordable animal-source foods per unit priority nutrient density, although not as affordable as organs and small fish, and they are still often inaccessible or unaffordable for people with limited resources (32, 35). Importantly, DGLVs and pulses are accessible and affordable sources of several priority micronutrients in most populations (32, 35). Further, traditional grains, including teff, quinoa, fonio, and millet, are at least moderately dense in iron, zinc, and folate and can also make significant contributions to nutrient adequacy. Lacto-ovo vegetarian diets rich in eggs, dairy, DGLVs, pulses, and traditional grains can provide adequate amounts of all six priority micronutrients. Carefully constructed vegan diets could provide adequate amounts of all six priority micronutrients for the general population, except vitamin B_12_, which would need to be consumed through fortified foods or supplements. However, population groups with increased nutritional requirements, such as pregnant women and children during the complementary feeding period, following a vegan diet likely also need fortification or supplementation for other micronutrients, such as iron, in addition to vitamin B_12_.

Importantly, to the extent possible, the pursuit of dietary nutrient adequacy for the global population should not come at the expense of increased environmental destruction. There may be some inevitable trade-offs between achieving micronutrient adequacy and minimizing the environmental impact of diets, but there is great potential to improve the sustainability of all types of foods using productive regenerative practices suitable to local ecosystems (36–38). Particular attention could be given to nutrient dense foods with the greatest potential for sustainable production. For example, seaweeds, bivalves, and small fish are generally highly nutrient dense and sustainable to produce (39). While there may be nutritional advantages of obtaining nutrients through foods (12–14), the very high iron requirements of more than 1.8 billion WRA globally might be challenging to achieve sustainably through foods alone and thus supplementation should be considered. Finally, plant-source foods generally have lower negative environmental impact than animal-source foods per unit protein, energy, or mass based on current production practices and existing metrics used to quantify environmental impact (40). However, this generalization may not hold when considering the higher bioavailable nutrient density of many animal-source foods as shown in the present analysis and others (34, 36), or when considering regenerative production practices and metrics that holistically quantify their environmental impacts (37, 38, 41).

Our study has several strengths. The methods are transparent and based on publicly available data, as has been recommended (42, 43). The food composition data is comprehensive and representative of diets in diverse contexts globally, unlike existing nutrient profiling systems, which are based solely on national food composition data, typically USDA FDC (42, 43), and we adjusted for differences in bioavailability of iron and zinc across foods. Similarly, recommended intakes are based on dietary reference values that are appropriate for global populations, including LMICs, and were calculated for the general adult population as well as groups with increased needs. Our ratings prioritize foods that are optimal sources of micronutrients known to be commonly lacking and causing significant health burdens in LMICs, in alignment with the recommendation to focus nutrient profiling models for LMICs on nutrient density of beneficial nutrients, rather than nutrients to limit (42). Lastly, the results are organized in clear and simple visualizations which are easily interpretable by non-technical audiences, including decision makers.

We focused on inherent priority micronutrient density and bioavailability and did not include fortified foods or address the overall role of food and diets in nutrient adequacy, infectious diseases, and NCDs and their broader impact on the global burden of disease (44). Other essential vitamins and minerals, including vitamin C, vitamin E, riboflavin, thiamin, niacin, potassium, and magnesium, can also be lacking in diets, but data is limited on how widespread these inadequacies are and their public health significance (1). Moreover, adequate calories (45), protein (46), and essential amino acids (47) and fatty acids (especially n-3 fatty acids) (48) are also often lacking and critically important for health. Furthermore, there are countless “non-essential” but nonetheless potentially beneficial compounds including fiber, phytonutrients, and bioactive compounds in plant and animal-source foods which play an important role in health and disease (12–14, 44). Finally, there are numerous compounds that are associated with increased risk of disease when consumed in excess, including sugar, sodium, *trans* fat, cosmetic additives, and contamination and biological hazards in unsafe food, among others, for which the type and level of processing often plays an important role (49–51).

Our analysis has important limitations. First, there are large differences in nutrient densities across food composition databases, which may reflect differences in varieties, production methods, soil conditions, fertilizer use, animal feed quality, culinary traditions, and/or technical and analytical quality of the underlying databases. Moreover, mineral densities have even been shown to vary geospatially within individual countries (52). Since the exact nutrient densities of any given food and context are unknown, we chose to use aggregate values to smooth out these variations, which contributes to the added value of our global food composition database. Second, in addition to significant differences across FCTs, there is sometimes high nutrient-density variance across foods within food groups, meaning that the ranking of a food group as a whole might not reflect the micronutrient density of the most (or least) nutrient-dense foods included. However, we chose to maintain these levels of aggregation because our selected food groups are more likely to be targeted in programming and policies than individual foods and match more closely with food groups in upcoming global diet quality monitoring data (53, 54). Third, country and regional FCTs only included a limited set of commonly consumed foods, which limited the breadth of foods included in our aggregated food composition database. For instance, we were unable to analyze many nutrient-dense wild or indigenous vegetables, nuts, seeds, pulses, and insects, or novel foods like ground eggshells. Fourth, while we adjusted for bioavailability of iron and zinc, actual bioavailability depends on the genetics and micronutrient status of the individual and their overall diet, including a broad set of enhancers and inhibitors. Finally, ratings are sensitive to categorical thresholds for quantities of calories and grams, which requires some attention when interpreting results, since foods near the thresholds could have been rated differently with only small changes in nutrient densities. Some of the differences in ratings across population groups could thus be due to small differences in nutrient densities for foods near thresholds.

These ratings are most applicable for populations in LMICs suffering from widespread micronutrient deficiencies. However, for population groups with increased needs in HICs, such as pregnant women and WRA, who may often be deficient in micronutrients such as iron, zinc, and folate, these results can also help identify relevant foods to prioritize. Importantly, diets should consist of a variety of foods with varying nutrient densities. Even adding just small amounts of particularly nutrient dense animal-source foods (for example, organs, small fish, and bivalves) to largely plant-based diets would go a long way toward ensuring adequacy of micronutrients commonly lacking. Future analyses should focus on understanding how to use these findings to improve food, agriculture, and nutrition policies and programs, which tend to focus on specific foods or food groups. Researchers could build on this work by incorporating additional foods and food groups, including eggshells (55) and wild or indigenous vegetables, nuts, seeds, pulses, and insects (56), many of which contain very high nutrient densities (57). Moreover, these ratings could be paired with broader diet quality metrics (54) and included as an additional way to assess food affordability, for example, by expanding on existing approaches (32, 35), as has been done for other nutrient profiling systems (58). Finally, these ratings could also be used to provide more nutritionally relevant indicators of the environmental impact of foods, for example, by quantifying impact in terms of nutrient density.

## Data Availability Statement

The original contributions presented in the study are included in the article/Supplementary Material, further inquiries can be directed to the corresponding author/s.

## Author Contributions

TB and FO designed the study, conducted the analyses, and wrote the manuscript. Both authors contributed to the article and approved the submitted version.

## Conflict of Interest

The authors declare that the research was conducted in the absence of any commercial or financial relationships that could be construed as a potential conflict of interest.

## Publisher’s Note

All claims expressed in this article are solely those of the authors and do not necessarily represent those of their affiliated organizations, or those of the publisher, the editors and the reviewers. Any product that may be evaluated in this article, or claim that may be made by its manufacturer, is not guaranteed or endorsed by the publisher.

## Abbreviations

ARs: average requirements
DGLVs: dark green leafy vegetables
EFSA: European Food Safety Authority
FCTs: food composition tables
FDC: FoodData Central
HICs: high-income countries
LMICs: low- and middle-income countries
NCDs: non-communicable diseases
UPFs: ultra-processed foods
WRA: women of reproductive age.

## References

[1] Beal T Massiot E Arsenault JE Smith MR Hijmans RJ. Global trends in dietary micronutrient supplies and estimated prevalence of inadequate intakes. *PLoS One.* (2017) 12:e0175554. 10.1371/journal.pone.0175554 28399168 PMC5388500

[2] Bailey RL West KP Black RE. The epidemiology of global micronutrient deficiencies. *Ann Nutr Metab.* (2015) 66(Suppl. 2):22–33. 10.1159/000371618 26045325

[3] WHO. *Vitamin and Mineral Nutrition Information System (VMNIS).* (2021). Available online at: https://www.who.int/teams/nutrition-and-food-safety/databases/vitamin-and-mineral-nutrition-information-system (accessed June 13, 2021)

[4] Beal T White JM Arsenault JE Okronipa H Hinnouho G-M Torlesse H Micronutrient gaps during the complementary feeding period in South Asia: a comprehensive nutrient gap assessment. *Nutr Rev.* (2021) 79(Suppl. 1):26–34. 10.1093/nutrit/nuaa144 33693912 PMC7947968

[5] White JM Beal T Chimanya K Arsenault JE Okronipa H Hinnouho G-M Micronutrient gaps during the complementary feeding period in Eastern and Southern Africa: a comprehensive nutrient gap assessment. *Nutr Rev.* (2021) 79(Suppl. 1):16–25. 10.1093/nutrit/nuaa142 33693910 PMC7947982

[6] Black RE Victora CG Walker SP Bhutta ZA Christian P de Onis M Maternal and child undernutrition and overweight in low-income and middle-income countries. *Lancet.* (2013) 382:427–51. 10.1016/S0140-6736(13)60937-X 23746772

[7] Sethi V Lahiri A Bhanot A Kumar A Chopra M Mishra R. *Adolescents, Diets and Nutrition: Growing Well in a Changing World.* New Delhi: UNICEF India (2019).

[8] Miller EM. Iron status and reproduction in US women: national health and nutrition examination survey, 1999-2006. *PLoS One.* (2014) 9:e112216. 10.1371/journal.pone.0112216 25375360 PMC4223055

[9] Smith MR Myers SS. Impact of anthropogenic CO2 emissions on global human nutrition. *Nat Clim Change.* (2018) 8:834–9. 10.1016/S2214-109X(15)00093-5

[10] Osendarp SJM Martinez H Garrett GS Neufeld LM De-Regil LM Vossenaar M Large-scale food fortification and biofortification in low- and middle-income countries: a review of programs, trends, challenges, and evidence gaps. *Food Nutr Bull.* (2018) 39:315–31. 10.1177/0379572118774229 29793357 PMC7473077

[11] FooDB. *Listing Compounds.* (2021). Available online at: https://foodb.ca/compounds (accessed June 13, 2021)

[12] van Vliet S Kronberg SL Provenza FD. Plant-based meats, human health, and climate change. *Front Sustain Food Syst.* (2020) 4:128. 10.3389/fsufs.2020.00128

[13] Barabási A-L Menichetti G Loscalzo J. The unmapped chemical complexity of our diet. *Nat Food.* (2020) 1:33–7. 10.1038/s43016-019-0005-1

[14] Jacobs DR Tapsell LC. Food, not nutrients, is the fundamental unit in nutrition. *Nutr Rev.* (2007) 65:439–50. 10.1111/j.1753-4887.2007.tb00269.x 17972438

[15] Aguilera JM. The food matrix: implications in processing, nutrition and health. *Crit Rev Food Sci Nutr.* (2019) 59:3612–29. 10.1080/10408398.2018.1502743 30040431

[16] Lane MM Davis JA Beattie S Gómez-Donoso C Loughman A O’Neil A Ultraprocessed food and chronic noncommunicable diseases: a systematic review and meta-analysis of 43 observational studies. *Obes Rev.* (2021) 22:e13146. 10.1111/obr.13146 33167080

[17] Hall KD Ayuketah A Brychta R Cai H Cassimatis T Chen KY Ultra-Processed diets cause excess calorie intake and weight gain: an inpatient randomized controlled trial of ad libitum food intake. *Cell Metab.* (2019) 30:67–77.e3. 10.1016/j.cmet.2019.05.008 31105044 PMC7946062

[18] European Food Safety Authority (Efsa). Dietary reference values for nutrients summary report. *EFSA Support Publ.* (2017) 14:e15121E. 10.2903/sp.efsa.2017.e15121

[19] Institute of Medicine Committee to Review Dietary Reference Intakes for Vitamin D and Calcium. *Dietary Reference Intakes for Calcium and Vitamin D.* Ross AC Taylor CL Yaktine AL Del Valle HB editors. Washington, DC: National Academies Press (2011). 21796828

[20] Allen LH Carriquiry AL Murphy SP. Perspective: proposed harmonized nutrient reference values for populations. *Adv Nutr.* (2020) 11:469–83. 10.1093/advances/nmz096 31701998 PMC7231601

[21] Fao/IZiNCG. *FAO/INFOODS/IZiNCG Global Food Composition Database for Phytate Version 1.0 – PhyFoodComp 1.0.* Rome: FAO (2018).

[22] U.S. Department Of Agriculture (USDA), Agricultural Research Service. *USDA FoodData Central.* (2021). Available online at: https://fdc.nal.usda.gov/ (accessed June 18, 2021).

[23] FAO. *INFOODS: FAO/INFOODS Databases.* (2021). Available online at: http://www.fao.org/infoods/infoods/tables-and-databases/faoinfoods-databases/en/ (accessed June 18, 2021)

[24] Valenzuela C López de Romaña D Olivares M Morales MS Pizarro F. Total iron and heme iron content and their distribution in beef meat and viscera. *Biol Trace Elem Res.* (2009) 132:103–11. 10.1007/s12011-009-8400-3 19475341

[25] Balder HF Vogel J Jansen MCJF Weijenberg MP van den Brandt PA Westenbrink S Heme and chlorophyll intake and risk of colorectal cancer in the Netherlands cohort study. *Cancer Epidemiol Biomarkers Prev.* (2006) 15:717–25. 10.1158/1055-9965.EPI-05-0772 16614114

[26] Lombardi-Boccia G Martinez-Dominguez B Aguzzi A. Total heme and non-heme iron in raw and cooked meats. *J Food Sci.* (2002) 67:1738–41. 10.1021/ac010279h 11575788

[27] Pourkhalili A Mirlohi M Rahimi E. Heme iron content in lamb meat is differentially altered upon boiling, grilling, or frying as assessed by four distinct analytical methods. *Sci World J.* (2013) 2013:e374030. 10.1155/2013/374030 23737716 PMC3662193

[28] Kabat GC Miller AB Jain M Rohan TE. A cohort study of dietary iron and heme iron intake and risk of colorectal cancer in women. *Br J Cancer.* (2007) 97:118–22. 10.1038/sj.bjc.6603837 17551493 PMC2359661

[29] Ronco A Espinosa E Calderon J. *A Case-Control Study on Heme/Non-heme Iron and Breast Cancer Risk 1 MedDocs Publishers of Creative Commons Attribution 4.0 International License Annals of Clinical Nutrition.* Reno, NV: MedDocs Publishers LLC (2018).

[30] Kongkachuichai R Napatthalung P Charoensiri R. Heme and nonheme iron content of animal products commonly consumed in Thailand. *J Food Composit Anal.* (2002) 15:389–98. 10.1006/jfca.2002.1080

[31] Taniguchi CN Dobbs J Dunn MA. Heme iron, non-heme iron, and mineral content of blood clams (*Anadara* spp.) compared to Manila clams (*V. philippinarum*), Pacific oysters (*C. gigas*), and beef liver (*B. taurus*). *J Food Composit Anal.* (2017) 57:49–55. 10.1016/j.jfca.2016.12.018

[32] Ryckman T Beal T Nordhagen S Chimanya K Matji J. Affordability of nutritious foods for complementary feeding in Eastern and Southern Africa. *Nutr Rev.* (2021) 79(Suppl. 1):35–51. 10.1093/nutrit/nuaa137 33693913 PMC7948081

[33] Hall KD Guo J Courville AB Boring J Brychta R Chen KY Effect of a plant-based, low-fat diet versus an animal-based, ketogenic diet on ad libitum energy intake. *Nat Med.* (2021) 27:344–53. 10.1038/s41591-020-01209-1 33479499

[34] Ortenzi F Beal T. Priority micronutrient density of foods for complementary feeding of young children (6–23 months) in South and Southeast Asia. *Front Nutr.* (2021) 8:785227. 10.3389/fnut.2021.785227 34993221 PMC8724761

[35] Ryckman T Beal T Nordhagen S Murira Z Torlesse H. Affordability of nutritious foods for complementary feeding in South Asia. *Nutr Rev.* (2021) 79(Suppl. 1):52–68. 10.1093/nutrit/nuaa139 33693914 PMC7948078

[36] Beal T. Achieving dietary micronutrient adequacy in a finite world. *One Earth.* (2021) 4:1197–200. 10.1016/j.oneear.2021.08.019

[37] LaCanne CE Lundgren JG. Regenerative agriculture: merging farming and natural resource conservation profitably. *PeerJ.* (2018) 6:e4428. 10.7717/peerj.4428 29503771 PMC5831153

[38] Kremen C Miles A. Ecosystem services in biologically diversified versus conventional farming systems: benefits, externalities, and trade-offs. *Ecol Soc.* (2012) 17:40.

[39] Gephart JA Henriksson PJG Parker RWR Shepon A Gorospe KD Bergman K Environmental performance of blue foods. *Nature.* (2021) 597:360–5. 10.1038/s41586-021-03889-2 34526707

[40] Poore J Nemecek T. Reducing food’s environmental impacts through producers and consumers. *Science.* (2018) 360:987–92. 10.1126/science.aaq0216 29853680

[41] Fenster TLD LaCanne CE Pecenka JR Schmid RB Bredeson MM Busenitz KM Defining and validating regenerative farm systems using a composite of ranked agricultural practices. *F1000Res.* (2021) 10:115. 10.12688/f1000research.28450.1 33763202 PMC7953916

[42] Miller GD Drewnowski A Fulgoni V Heaney RP King J Kennedy E. It is time for a positive approach to dietary guidance using nutrient density as a basic principle. *J Nutr.* (2009) 139:1198–202. 10.3945/jn.108.100842 19339707

[43] Drewnowski A Amanquah D Gavin-Smith B. Perspective: how to develop nutrient profiling models intended for global use: a manual. *Adv Nutr.* (2021) 12:609–20. 10.1093/advances/nmab018 33724302 PMC8166553

[44] Afshin A Sur PJ Fay KA Cornaby L Ferrara G Salama JS Health effects of dietary risks in 195 countries, 1990–2017: a systematic analysis for the global burden of disease study 2017. *Lancet.* (2019) 393:1958–72. 10.1016/S0140-6736(19)30041-8 30954305 PMC6899507

[45] Fao, Ifad, Unicef, Wfp, Who. *The State of Food Security and Nutrition in the World 2020: Transforming food systems for Affordable Healthy Diets.* Rome: FAO (2020) p.320. 10.4060/ca9692en

[46] Wu G Fanzo J Miller DD Pingali P Post M Steiner JL Production and supply of high-quality food protein for human consumption: sustainability, challenges, and innovations. *Ann N Y Acad Sci.* (2014) 1321:1–19. 10.1111/nyas.12500 25123207

[47] Semba RD Shardell M Sakr Ashour FA Moaddel R Trehan I Maleta KM Child stunting is associated with low circulating essential amino acids. *EBioMedicine.* (2016) 6:246–52. 10.1016/j.ebiom.2016.02.030 27211567 PMC4856740

[48] Simopoulos AP. Essential fatty acids in health and chronic disease. *Am J Clin Nutr.* (1999) 70:560s–9s. 10.1093/ajcn/70.3.560s 10479232

[49] Mozaffarian D. Dietary and policy priorities for cardiovascular disease, diabetes, and obesity. *Circulation.* (2016) 133:187–225. 10.1161/CIRCULATIONAHA.115.018585 26746178 PMC4814348

[50] Neufeld LM Hendriks S Hugas M. *Healthy Diet: A Definition for the United Nations Food Systems Summit 2021.* New York, NY: United Nations (2020). 38285853

[51] Monteiro CA Cannon G Levy RB Moubarac J-C Louzada ML Rauber F Ultra-processed foods: what they are and how to identify them. *Public Health Nutr.* (2019) 22:936–41. 10.1017/S1368980018003762 30744710 PMC10260459

[52] Gashu D Nalivata PC Amede T Ander EL Bailey EH Botoman L The nutritional quality of cereals varies geospatially in Ethiopia and Malawi. *Nature.* (2021) 594:71–6. 10.1038/s41586-021-03559-3 34012114 PMC8172382

[53] Herforth AW Wiesmann D Martínez-Steele E Andrade G Monteiro CA. Introducing a suite of low-burden diet quality indicators that reflect healthy diet patterns at population level. *Curr Dev Nutr.* (2020) 4:nzaa168. 10.1093/cdn/nzaa168 33344879 PMC7723758

[54] Herforth A Beal T Rzepa A. *Global Diet Quality Project Aims to Bridge Data Gap. Gallup.com.* (2020). Available online at: https://news.gallup.com/opinion/gallup/321968/global-diet-quality-project-aims-bridge-data-gap.aspx (accessed June 29, 2021).

[55] Bartter J Diffey H Yeung YH O’Leary F Häsler B Maulaga W Use of chicken eggshell to improve dietary calcium intake in rural sub-Saharan Africa. *Matern Child Nutr.* (2018) 14:e12649. 10.1111/mcn.12649 30332539 PMC6221107

[56] Smith MR Stull VJ Patz JA Myers SS. Nutritional and environmental benefits of increasing insect consumption in Africa and Asia. *Environ Res Lett.* (2021) 16:065001. 10.1088/1748-9326/abf06c

[57] Nyirenda D Musukwa M Mugode R Shindano J. *Zambia Food Composition Tables.* 4th ed. Lusaka: National Food and Nutrition Commission (2009).

[58] Drewnowski A Smith J Fulgoni VL. The new hybrid nutrient density score NRFh 4:3:3 tested in relation to affordable nutrient density and healthy eating index 2015: analyses of NHANES data 2013–16. *Nutrients.* (2021) 13:1734. 10.3390/nu13051734 34065287 PMC8160959

