# Corrigendum: Priority Micronutrient Density in Foods

**DOI:** 10.3389/fnut.2022.908592

**Published:** 2022-04-25

**Authors:** Ty Beal, Flaminia Ortenzi

**Affiliations:** ^1^Knowledge Leadership, Global Alliance for Improved Nutrition, Washington, DC, United States; ^2^Department of Environmental Science and Policy, University of California, Davis, Davis, CA, United States; ^3^Knowledge Leadership, Global Alliance for Improved Nutrition, Geneva, Switzerland

**Keywords:** nutrient density, micronutrient deficiencies, animal-source foods, organs, shellfish, fish, dark green leafy vegetables, ruminant meat

In the original article, there was an error in Table 1 as published. The values for Zinc in the row “Adults 25+” are incorrect. Column R was written as “9.4” when it should be “8.5”. Column SR was “11.7” when it should be “10.5”. Column SU was “14.0” when it should be “12.5” and Column U was “16.3” when it should be “14.5.” The corrected Table 1 appears below.

**Table 1:** Recommended nutrient intakes for select groups.

**Group**	**AER** **(kcal)**	**Vit A** **(mcg RAE)**	**Folate** **(mcg DFE)**	**Vit B_12_** **(mcg)**	**Calcium** **(mg)**	**Iron** **(mg)^1^**			**Zinc** **(mg)^2^**			
						**20%**	**15%**	**10%**	**R**	**SR**	**SU**	**U**
Children 2–4	1246	230	128	1.0	590	7.4	9.8	14.8	3.2	3.9	4.7	5.5
Adolescents 10–19	2296	632	292	2.2	1085	9.9	13.2	19.8	8.3	9.9	11.4	13.0
Women 15–49	2305	637	325	2.4	977	15.9	21.2	31.8	8.0	9.6	11.1	12.6
Pregnant women 15–49	2583	700	600	2.6	977	24.3	32.4	48.6	9.1	10.9	12.6	14.3
Adults 25+^3^	2227	694	328	2.4	950	7.0	10.0	14.0	8.5	10.5	12.5	14.5

*Average energy requirements for a moderately active individual and recommended intakes for vitamin A, folate, calcium and zinc from the European Food Safety Authority (18). Recommended intakes for iron and vitamin B_12_ from the Institute of Medicine (19).*

1 *Percentages represent different levels of bioavailability that correspond with the possible classifications of each food in the analysis.*

2 *Assuming 300 mg phytate/day and 44% absorption for refined (R) diets, 600 mg phytate/day and 35% absorption for semi-refined (SR) diets, 900 mg phytate/day and 30% absorption for semi-unrefined (SU) diets, and 1200 mg phytate/day and 26% absorption for unrefined (U) diets.*

3 *Includes both men and women. AER, Average Energy Requirement; DFE, dietary folate equivalent; R, refined; RAE, retinol activity equivalent; SR, semi-refined; SU, semi-unrefined; U, unrefined; Vit, vitamin*.

The authors apologize for this error and state that this does not change the scientific conclusions of the article in any way. The original article has been updated.

## Publisher's Note

All claims expressed in this article are solely those of the authors and do not necessarily represent those of their affiliated organizations, or those of the publisher, the editors and the reviewers. Any product that may be evaluated in this article, or claim that may be made by its manufacturer, is not guaranteed or endorsed by the publisher.

