# Supplementary material for: Priority Micronutrient Density in Foods
Source: Front Nutr. 2022 Mar 7;9:806566. doi: 10.3389/fnut.2022.806566 (PMC8936507; doi:10.3389/fnut.2022.806566)
Supplement: Supplementary file 1 [file Data_Sheet_1.PDF]

Supplementary material

Table of Contents

Methods **........ 2**

Supplemental Table 1: Global food composition database, with standard deviations reported in () as a measure of variability of included values. **........ 4**

Supplemental Table 2: first level granular food composition database of all plant-source foods **........ 7**

Supplemental Table 3: first level granular food composition database of all animal-source foods **........ 11**

Supplemental Table 4: second level granular food composition database of aggregated plant-source foods **........ 16**

Supplemental Table 5: second level granular food composition database of individual plant-source foods **........ 37**

Supplemental Table 6: second level granular food composition database of aggregated animal-source foods **........ 40**

Supplemental Table 7: second level granular food composition database of individual animal-source foods **........ 47**

Supplemental Fig. 1 | Calories and grams needed to provide an average of one-third of recommended intakes of vitamin A, folate, vitamin B_{12}, calcium, iron, and zinc for children 2–4 years. **........ 55**

Supplemental Fig. 2 | Calories and grams needed to provide an average of one-third of recommended intakes of vitamin A, folate, vitamin B_{12}, calcium, iron, and zinc for adolescents. **........ 56**

Supplemental Fig. 3 | Calories and grams needed to provide an average of one-third of recommended intakes of vitamin A, folate, vitamin B_{12}, calcium, iron, and zinc for pregnant women. **........ 57**

Supplemental Fig. 4 | Aggregate and individual micronutrient density scores for children 2–4 years. **........ 58**

Supplemental Fig. 5 | Aggregate and individual micronutrient density scores for adolescents. **........ 59**

Supplemental Fig. 6 | Aggregate and individual micronutrient density scores for pregnant women. **........ 60**

Methods

The main rationale for including values from USDA FoodData Central (FDC) was to use them as a reference to verify reliability of values from the selected national and regional food composition tables (FCTs) from LMICs. In addition, values from FDC have occasionally been used to replace missing values in other included FCTs. Such cases have been noted in the "Comments" column of the granular food composition databases (Tables 1–6). The "Comments" column also reports cases in which weight yields and nutrient retention factors were applied to calculate values for cooked foods, starting from corresponding values for raw foods available in the selected FCTs[1]. Moreover, three special cases (teff, fonio, and small dried fish) were mentioned in the "Comments" column, where values from the literature were included in addition to values from the selected FCTs, as the latter were considered insufficient or not fully reliable.

To calculate iron bioavailability of foods, we assumed 10% and 25% bioavailability for non-heme iron and heme iron, respectively[2]. The following calculations were executed:

- Ruminant meat, 68% heme iron content (including beef, goat, lamb/mutton): 68% * 0.25 + 32% * 0.10 = 20% bioavailability
- Pork, 39% heme iron content: 39% * 0.25 + 61% * 0.10 = 16% bioavailability
- Chicken, fish and seafood, eggs and dairy, 26% heme iron content: 26% * 0.25 + 74% * 0.10 = 14% bioavailability
- All other meat, including offal, 40% heme iron content: 40% * 0.25 + 60% * 0.10 = 16% bioavailability
- All plant-source foods, 0% heme iron content: 0% * 0.25 + 100% * 0.10 = 10% bioavailability

As a result of these calculations, we classified foods into one of three levels of iron absorption: 20% for ruminant meat, 15% for all other animal-source foods, and 10% for all plant-source foods.

Regarding zinc, as mentioned in the main text, foods were classified into four levels of zinc absorption, based on the amount of phytate contained in each food in a portion equivalent to one-third of daily mass intake, assuming an energy density of 1.3 kcal/g and considering average requirements for energy for women of reproductive age[3]. The four levels of zinc absorption were defined using the lower limits of thresholds on daily phytate intake identified by European Food Safety Authority[3], which considers the diet as a whole rather than phytate content of individual foods. Indeed, any food is consumed together with a variety of other foods as part of the broader diet, which will likely contribute additional phytate. While these foods constituting the hypothetical remaining two-thirds of the diet may increase the total dietary phytate intake to a higher category, we wanted zinc absorption to reflect bioavailability when attempting to moderate overall dietary phytate, because of its anti-nutrient effect on mineral absorption. Therefore, the four categories for zinc absorption were based on these phytate ranges in the selected food:

- Refined diet: ≤ 300 mg
- Semi-refined: 301–600 mg
- Semi-unrefined: 601–900 mg
- Unrefined: > 900 mg

The refined diet category assumes the lowest phytate intake range, resulting in the highest zinc absorption percentage, while the unrefined diet category assumes the highest phytate intake range, resulting in the lowest zinc absorption percentage. Semi-refined and semi-unrefined diets position themselves in the middle, with intermediate phytate intake ranges, leading to intermediate zinc absorption percentages.

References

1. Bognar, A. (Bundesforschungsanstalt fuer E. Tables on weight yield of food and retention factors of food constituents for the calculation of nutrient composition of cooked foods (dishes). (2002).
2. Beal, T., Massiot, E., Arsenault, J. E., Smith, M. R. & Hijmans, R. J. Global trends in dietary micronutrient supplies and estimated prevalence of inadequate intakes. *PLOS ONE* **12**, e0175554 (2017).
3. European Food Safety Authority (EFSA). Dietary Reference Values for nutrients Summary report. *EFSA Supporting Publications* **14**, e15121E (2017).

Supplemental Table 1: Global food composition database, with standard deviations reported in () as a measure of variability of included values.

Food group	Food (100 g)	Energy (kcal)	Vit A (mcg RAE)	Folate (mcg DFE)	Vit B_{12} (mcg)	Calcium (mg)	Iron (mg)	Zinc (mg)	Iron Abs	Zinc Abs	Phytate (mg)
Pulses	Pulses	133.63 (17.59)	1.0 (0.41)	88.38 (55.04)	0	28.58 (4.51)	2.37 (0.46)	1.19 (0.09)	0.10	0.26	440.95 (106.18)
Whole grains	Whole grains	186.13(75.45)	0.0 (0.00)	15.13 (2.74)	0	16.84 (9.65)	1.65 (0.96)	1.19 (0.55)	0.10	0.26	460.81 (47.34)
Refined grains	Refined grains	133.38 (14.87)	0.0 (0.00)	4.61 (2.04)	0	8.9 (2.81)	0.54 (0.31)	0.63 (0.16)	0.10	0.44	44.59 (5.89)
Unrefined grain products	Unrefined grain products	166.00 (89.72)	2.33 (4.04)	31.33 (22.05)	0	29.33 (18.88)	1.94 (1.80)	1.01 (0.57)	0.10	0.30	128.66 (75.42)
Refined grain products	Refined grain products	167.88 (39.40)	0 (0.00)	12.38 (14.84)	0	11.63 (5.50)	0.78 (0.34)	0.53 (0.12)	0.10	0.44	49.26 (0.00)
Traditional grains	Sorghum	142.25 (1.77)	0.25 (0.35)	19.5 (0.71)	0	8.5 (1.41)	2.58 (0.74)	0.77 (0.04)	0.10	0.26	272 (0.00)
Traditional grains	Millet	148.00 (15.19)	0 (0.45)	26.56 (13.94)	0	10 (6.60)	2.6 (2.22)	1.02 (0.38)	0.10	0.26	200 (0.00)
Traditional grains	Teff	149.00 (67.88)	0 (0.00)	41.5 (33.23)	0	49 (31.97)	4.3 (1.68)	1.11 (0.57)	0.10	0.26	284.04 (191.88)
Traditional grains	Fonio	138.50 (N/A)	0.5 (N/A)	36 (N/A)	0	11.91 (10.03)	2.81 (1.40)	1.1 (0.28)	0.10	0.30	110 (N/A)
Traditional grains	Quinoa	114.97 (7.11)	0 (0.00)	42.52 (0.74)	0	16.54 (0.65)	1.98 (0.69)	1.02 (0.11)	0.10	0.26	554.14 (0.00)
Starchy roots, tubers and plantains	Roots, tubers & plantains	110.75 (14.55)	14 (14.09)	11.63 (3.59)	0	16.5 (9.01)	0.72 (0.10)	0.27 (0.04)	0.10	0.44	12.85 (8.87)
Nuts	Nuts	593.88 (18.38)	0.25 (0.50)	72 (17.66)	0	62.25 (15.65)	4.06 (0.56)	2.96 (0.49)	0.10	0.26	670.38 (109.68)
Seeds	Seeds	579.25 (25.09)	1 (0.82)	98.25 (2.18)	0	333.38 (428.36)	7.55 (1.21)	5.52 (1.23)	0.10	0.26	652.81 (584.46)
DGLVs	Dark green leafy vegetables	30.00 (8.04)	251.75 (46.56)	57.38 (34.12)	0	148.17 (84.69)	2.15 (0.49)	0.44 (0.20)	0.10	0.44	17.13 (14.06)
Vit A-rich fruits and vegetables, excl DGLVs	Vit A-rich fruits/vegetables	39.75 (3.23)	123.88 (32.16)	24.13 (5.20)	0	20.16 (5.21)	0.47 (0.10)	0.19 (0.02)	0.10	0.44	24.38 (1.25)

Other vegetables, excl DGLVs and vit A-rich vegetables	Other vegetables	27.88 (5.27)	19.5 (7.36)	17 (3.03)	0	17.5 (5.05)	0.51 (0.17)	0.24 (0.08)	0.10	0.44	10.08 (3.73)
Other fruits, excl vit A-rich fruits	Other fruits	64.50 (5.12)	3.75 (0.96)	18.75 (2.63)	0	11.13 (1.65)	0.36 (0.10)	0.22 (0.20)	0.10	0.44	10.25 (0.50)
Eggs	Eggs	156.00 (13.29)	162.5 (39.91)	44.5 (9.74)	1.11 (0.29)	49.5 (23.03)	1.6 (0.41)	1.1 (0.62)	0.15	0.44	0.00
Milk and dairy	Fresh cow milk	67.00 (4.58)	44 (7.52)	5 (2.41)	0.44 (0.08)	120 (6.29)	0.1 (1.09)	0.4 (0.09)	0.15	0.44	0.00
Milk and dairy	Cooked cow milk	61.00 (8.73)	39 (7.39)	5 (1.87)	0.45 (0.26)	115.94 (11.31)	0.1 (1.15)	0.45 (0.15)	0.15	0.44	0.00
Milk and dairy	Fresh goat milk	71.50 (9.37)	34.5 (13.93)	1 (0.00)	0.07 (0.11)	143 (27.65)	0.1 (0.19)	0.33 (0.04)	0.15	0.44	0.00
Milk and dairy	Yoghurt	77.00 (11.50)	27 (3.39)	7 (3.13)	0.37 (0.08)	121 (30.08)	0.1 (0.07)	0.38 (0.13)	0.15	0.44	0.00
Milk and dairy	Cheese	358.50 (24.60)	212.88 (22.80)	15.63 (9.32)	1 (0.27)	707.13 (91.75)	0.5 (0.18)	3.03 (0.36)	0.15	0.44	0.00
Ruminant meat	Beef	273.25 (64.19)	2.5 (8.61)	6.33 (3.90)	2.26 (0.54)	10 (5.16)	2.52 (0.89)	5.3 (0.53)	0.20	0.44	0.00
Ruminant meat	Goat	141.50 (52.07)	0 (5.25)	4 (1.36)	1.19 (0.31)	17 (11.49)	3.445 (0.89)	5.095 (1.25)	0.20	0.44	0.00
Ruminant meat	Lamb/mutton	227.28 (105.75)	2.405 (5.22)	5.93 (5.13)	2.53 (0.89)	10.71 (5.69)	1.81 (0.77)	3.655 (1.59)	0.20	0.44	0.00
Other meat	Pork	241.60 (73.75)	1.8 (2.25)	2.6 (2.78)	0.66 (0.21)	23 (7.32)	1.55 (0.71)	2.6 (0.58)	0.15	0.44	0.00
Poultry	Chicken	229.84 (37.62)	27.585 (12.75)	4.27 (2.75)	0.27 (0.08)	13.25 (3.32)	0.89 (0.30)	1.535 (0.39)	0.15	0.44	0.00
Organ meats	Beef liver	176.50 (25.57)	8645 (9076.63)	256.5 (29.52)	76.22 (45.43)	7 (14.73)	8.99 (3.67)	5.23 (1.11)	0.15	0.44	0.00
Organ meats	Goat/lamb liver	206.75 (22.25)	18093.3 (10456.75)	349 (112.50)	85.05 (3.95)	10.75 (2.25)	9.97 (0.73)	6.3 (0.47)	0.15	0.44	0.00
Organ meats	Chicken liver	140.85 (25.44)	3492 (2390.69)	533.59 (278.16)	16.12 (3.52)	10.38 (2.48)	10.34 (2.30)	3.41 (0.36)	0.15	0.44	0.00
Organ meats	Pork liver	113.75 (33.93)	4796.26 (486.90)	147.76 (13.80)	15.76 (1.90)	10 (1.38)	17.92 (2.45)	4.58 (1.27)	0.15	0.44	0.00
Organ meats	Heart	111.54 (47.90)	3.5 (1.53)	24.54 (11.77)	5.13 (2.96)	7.41 (1.91)	4.4 (1.19)	3.12 (0.29)	0.15	0.44	0.00

Organ meats	Spleen	114.28 (49.10)	0 (0.00)	3.33 (0.95)	4.17 (1.20)	10.4 (3.68)	35.84 (4.05)	2.47 (1.52)	0.15	0.44	0.00
Organ meats	Kidney	97.47 (43.49)	68.93 (23.87)	33.58 (26.44)	14.49 (8.38)	11.65 (5.22)	5.56 (1.07)	2.39 (1.12)	0.15	0.44	0.00
Fish and seafood	Fresh fish	123.42 (8.86)	9.86 (186.00)	9.69 (3.79)	1.81 (12.10)	38.62 (2360.00)	0.81 (10.00)	0.74 (10.15)	0.15	0.44	0.00
Fish and seafood	Small dried fish	294.00 (61.82)	186 (218.53)	37 (12.73)	12.1 (27.68)	2360 (887.00)	10 (13.70)	10.15 (3.82)	0.15	0.44	0.00
Fish and seafood	Canned fish, without bones	152.50 (16.50)	13.83 (7.49)	4.5 (1.32)	2.16 (0.44)	12.33 (4.16)	1.25 (0.15)	0.64 (0.13)	0.15	0.44	0.00
Fish and seafood	Canned fish, with bones	201.17 (18.32)	39.83 (10.75)	8.5 (2.18)	5.76 (1.62)	252 (19.92)	2.28 (0.29)	1.41 (0.32)	0.15	0.44	0.00
Fish and seafood	Crustaceans	88.60 (24.37)	8.11 (6.40)	11.84 (5.36)	1.3 (0.67)	73.85 (17.97)	1.23 (0.77)	2.2 (1.33)	0.15	0.44	0.00
Fish and seafood	Bivalves	87.20 (62.22)	76.93 (26.79)	19.11 (23.28)	23.5 (16.79)	113.43 (122.78)	4.81 (2.46)	3.59 (3.47)	0.15	0.44	0.00

Supplemental Table 2: first level granular food composition database of all plant-source foods

Food group	Food (100 g)	National FCT/regional composite FCT	Energy (kcal)	Vit A (mcg RAE)	Folate (mcg DFE)	Vit B_{12} (mcg)	Ca (mg)	Iron (mg)	Zinc (mg)	Phytate (mg)	Comments
Pulses	Pulses	USDA	121.50	0.50	157.50	0.00	26.00	2.92	1.28	404.56	
	Pulses	Sub-Saharan Africa	120.00	1.00	52.00	0.00	26.00	2.10	1.14	316.40	
	Pulses	South/SouthEast Asia	158.00	1.50	37.00	0.00	27.00	2.55	1.25	476.48	
	Pulses	Latin America	135.00	1.00	107.00	0.00	35.30	1.90	1.10	566.35	
	Pulses	**Composite**	133.63	1.00	88.38	0.00	28.58	2.37	1.19	440.95	
Whole grains	Whole grains	USDA	122.00	0.00	16.00	0.00	3.00	0.56	0.71	437.58	
	Whole grains	Sub-Saharan Africa	139.50	0.00	11.50	0.00	17.50	1.20	0.75	426.36	
	Whole grains	South/SouthEast Asia	193.26	0.00	14.94	0.00	23.03	2.75	1.49	448.80	
	Whole grains	Latin America	289.78	0.00	18.06	0.00	23.83	2.08	1.83	530.48	
	Whole grains	**Composite**	186.13	0.00	15.13	0.00	16.84	1.65	1.19	460.81	
Refined grains	Refined grains	USDA	130.00	0.00	6.00	0.00	11.00	0.46	0.69	47.47	
	Refined grains	Sub-Saharan Africa	118.50	0.00	2.00	0.00	6.00	0.30	0.42	47.57	
	Refined grains	South/SouthEast Asia	131.00	0.00	4.00	0.00	7.00	0.40	0.60	35.76	
	Refined grains	Latin America	154.00	0.00	6.44	0.00	11.60	1.00	0.80	47.57	
	Refined grains	**Composite**	133.38	0.00	4.61	0.00	8.90	0.54	0.63	44.59	
Unrefined cereal products	Unrefined cereal products	USDA	149.00	0.00	37.00	0.00	13.00	0.90	1.14	137.99	
	Unrefined cereal products	Sub-Saharan Africa	86.00	7.00	7.00	0.00	25.00	0.90	0.39	198.98	
	Unrefined cereal products	Latin America	263.00	0.00	50.00	0.00	50.00	4.01	1.50	49.00	
	Unrefined cereal products	**Composite**	166.00	2.33	31.33	0.00	29.33	1.94	1.01	128.66	No values for the Asian region
Refined cereal products	Refined cereal products	USDA	158.00	0.00	7.00	0.00	7.00	0.50	0.51	49.26	
	Refined cereal products	Sub-Saharan Africa	137.00	0.00	3.00	0.00	9.00	0.50	0.37	49.26	
	Refined cereal products	South/SouthEast Asia	151.00	0.00	5.00	0.00	11.00	0.90	0.57	49.26	

	Refined cereal products	Latin America	225.50	0.00	34.50	0.00	19.50	1.20	0.65	49.26	
	Refined cereal products	**Composite**	167.88	0.00	12.38	0.00	11.63	0.78	0.53	49.26	
Traditional grains	Sorghum	USDA									Not available in USDA FoodData Central
	Sorghum	Kenya	141.00	0.50	20.00	0.00	7.50	2.05	0.74	272.00	
	Sorghum	Western Africa	143.50	0.00	19.00	0.00	9.50	3.10	0.80	272.00	
	Sorghum	**Composite**	142.25	0.25	19.50	0.00	8.50	2.58	0.77	272.00	Only values from the African region
Traditional grains	Millet	USDA	119.00	0.00	19.00	0.00	3.00	0.63	0.91	200.00	
	Millet	Kenya	148.00	1.00	50.50	0.00	15.00	2.60	1.69	200.00	
	Millet	Western Africa	152.00	0.00	47.00	0.00	10.00	6.30	1.02	200.00	
	Millet	Bangladesh	145.42	0.00	26.56	0.00	17.50	3.33	1.26	200.00	
	Millet	Colombia	158.75	0.00	26.56	0.00	3.33	1.25	0.69	200.00	
	Millet	**Composite**	148.00	0.00	26.56	0.00	10.00	2.60	1.02	200.00	
Traditional grains	Teff	USDA	101.00	0.00	18.00	0.00	49.00	2.05	1.11		
	Teff	Western Africa	197.00	0.00	65.00	0.00	101.00	4.30	1.94		Only values from the African region
	Teff	Nyachoti 2021					42.78	5.34	0.84		Values from the literature
	Teff	**Composite**	149.00	0.00	41.50	0.00	49.00	4.30	1.11	284.04	Used average phytate of the four other traditional grains
Traditional grains	Fonio	USDA									Not available in USDA FoodData Central
	Fonio	Western Africa	138.50	0.50	36.00	0.00	19.00	3.80	1.30	110.00	Only values from the African region
	Fonio	Koroch & Ballogou 2013					4.81	1.82	0.90		Values from the literature
	Fonio	**Composite**	138.50	0.50	36.00	0.00	11.91	2.81	1.10	110.00	
Traditional grains	Quinoa	USDA	120.00	0.00	42.00	0.00	17.00	1.49	1.09	554.14	
	Quinoa	Colombia	109.94	0.00	43.04	0.00	16.08	2.46	0.94	554.14	Only values from the Latin American region
	Quinoa	**Composite**	114.97	0.00	42.52	0.00	16.54	1.98	1.02	554.14	

Nuts	Nuts	USDA	576.50	0.00	62.00	0.00	75.00	3.83	3.20	709.50
	Nuts	Sub-Saharan Africa	595.00	0.00	60.00	0.00	45.00	4.10	2.24	582.00
	Nuts	South/SouthEast Asia	585.00	1.00	98.00	0.00	76.00	4.80	3.09	582.00
	Nuts	Latin America	619.00	0.00	68.00	0.00	53.00	3.49	3.30	808.00
	Nuts	**Composite**	593.88	0.25	72.00	0.00	62.25	4.06	2.96	670.38
Seeds	Seeds	USDA	569.00	0.00	97.50	0.00	120.50	7.44	6.23	381.24
	Seeds	Sub-Saharan Africa	585.00	1.00	101.50	0.00	91.00	7.10	4.05	282.00
	Seeds	South/SouthEast Asia	552.00	1.00	97.00	0.00	147.00	6.40	5.00	423.00
	Seeds	Latin America	611.00	2.00	97.00	0.00	975.00	9.24	6.80	1525.00
	Seeds	**Composite**	579.25	1.00	98.25	0.00	333.38	7.55	5.52	652.81
Starchy roots, tubers and plantains	Starchy roots, tubers and plantains	USDA	104.50	29.00	15.50	0.00	14.50	0.60	0.27	12.00
	Starchy roots, tubers and plantains	Sub-Saharan Africa	109.00	1.00	13.00	0.00	15.00	0.80	0.26	24.50
	Starchy roots, tubers and plantains	South/SouthEast Asia	98.00	3.00	7.00	0.00	29.00	0.80	0.32	12.00
	Starchy roots, tubers and plantains	Latin America	131.50	23.00	11.00	0.00	7.50	0.68	0.22	2.91
	Starchy roots, tubers and plantains	**Composite**	110.75	14.00	11.63	0.00	16.50	0.72	0.27	12.85
Dark green leafy vegetables (DGLV)	DGLV	USDA	21.00	306.00	38.00	0.00	58.00	2.26	0.27	23.01
	DGLV	Sub-Saharan Africa	40.00	195.00	47.50	0.00	262.00	2.80	0.72	34.00
	DGLV	South/SouthEast Asia	32.00	266.00	36.00	0.00	145.00	1.75	0.35	8.51
	DGLV	Latin America	27.00	240.00	108.00	0.00	127.66	1.80	0.40	3.00
	DGLV	**Composite**	30.00	251.75	57.38	0.00	148.17	2.15	0.44	17.13
Vit A-rich fruits and vegetables, excl DGLV	Vit A-rich fruits and vegetables, excl DGLV	USDA	40.50	147.50	20.00	0.00	13.50	0.39	0.18	22.50

	Vit A-rich fruits and vegetables, excl DGLV	Sub-Saharan Africa	42.00	88.50	24.00	0.00	19.40	0.60	0.20	25.00
	Vit A-rich fruits and vegetables, excl DGLV	South/SouthEast Asia	35.00	105.00	21.00	0.00	26.00	0.50	0.17	25.00
	Vit A-rich fruits and vegetables, excl DGLV	Latin America	41.50	154.50	31.50	0.00	21.75	0.40	0.22	25.00
	Vit A-rich fruits and vegetables, excl DGLV	**Composite**	39.75	123.88	24.13	0.00	20.16	0.47	0.19	24.38
Other fruits, excl vit A-rich fruits	Other fruits, excl vit A-rich fruits	USDA	60.50	3.00	19.00	0.00	11.00	0.28	0.11	10.00
	Other fruits, excl vit A-rich fruits	Sub-Saharan Africa	63.00	4.00	20.00	0.00	9.00	0.30	0.16	10.00
	Other fruits, excl vit A-rich fruits	South/SouthEast Asia	62.50	5.00	15.00	0.00	11.50	0.35	0.09	10.00
	Other fruits, excl vit A-rich fruits	Latin America	72.00	3.00	21.00	0.00	13.00	0.50	0.52	11.00
	Other fruits, excl vit A-rich fruits	**Composite**	64.50	3.75	18.75	0.00	11.13	0.36	0.22	10.25
Other vegetables, excl DGLV & vit A-rich veg	Other vegetables, excl DGLV & vit A-rich veg	USDA	22.50	20.50	15.50	0.00	13.50	0.33	0.17	13.00
	Other vegetables, excl DGLV & vit A-rich veg	Sub-Saharan Africa	28.00	26.00	16.00	0.00	19.00	0.70	0.26	13.00
	Other vegetables, excl DGLV & vit A-rich veg	South/SouthEast Asia	26.00	9.00	15.00	0.00	24.00	0.60	0.34	9.10
	Other vegetables, excl DGLV & vit A-rich veg	Latin America	35.00	22.50	21.50	0.00	13.50	0.40	0.20	5.20
	Other vegetables, excl DGLV & vit A-rich veg	**Composite**	27.88	19.50	17.00	0.00	17.50	0.51	0.24	10.08

Supplemental Table 3: first level granular food composition database of all animal-source foods

Food group	Food (100 g)	National FCT/regional composite FCT	Energy (kcal)	Vit A (mcg RAE)	Folate (mcg DFE)	Vit B_{12} (mcg)	Ca (mg)	Iron (mg)	Zinc (mg)	Phytate (mg)	Comments
Eggs	Chicken egg	USDA	155.00	149.00	44.00	1.10	50.00	1.20	1.10	0.00	
	Chicken egg	Kenya	134.00	177.00	66.00	1.00	51.00	1.60	1.10	0.00	
	Chicken egg	Malawi	164.00	74.00	38.00	1.80	43.00	2.00	1.28	0.00	
	Chicken egg	Bangladesh	158.00	178.00	45.00	1.10	35.00	1.80	2.54	0.00	Used vitB12 from FDC
	Chicken egg	Colombia	145.00	176.00	51.00	1.15	49.00	1.60	0.90	0.00	
	Chicken egg	Mexico	170.92	149.00	44.00	1.11	100.06	0.88	0.95	0.00	
	Chicken egg	**Composite**	156.50	162.50	44.50	1.11	49.50	1.60	1.10	0.00	
Ruminant meat	Beef	USDA	273.25	2.75	7.25	2.26	10.00	2.52	5.30	0.00	
	Beef	Western Africa	303.00	19.50	11.50	1.20	18.50	1.85	5.19	0.00	
	Beef	Laos	170.00	2.50	2.00	1.65	9.00	3.95	6.30	0.00	
	Beef	Colombia	189.67	1.67	6.33	2.35	6.67	3.07	5.23	0.00	
	Beef	**Composite**	231.46	2.63	6.79	1.96	9.50	2.80	5.27	0.00	
Ruminant meat	Goat	USDA	146.00	0.00	5.00	1.18	17.00	3.69	5.21	0.00	
	Goat	Western Africa	206.00	10.50	3.00	1.80	35.50	3.20	4.98	0.00	
	Goat	Bangladesh	78.77	0.00	2.37	1.19	6.85	1.82	2.67	0.00	Used vitB12 from FDC
	Goat	Colombia	137.00	0.00	5.00	1.19	17.00	3.70	5.30	0.00	
	Goat	**Composite**	141.50	0.00	4.00	1.19	17.00	3.45	5.10	0.00	
Ruminant meat	Lamb/mutton	USDA	275.75	0.00	12.75	2.96	16.25	2.69	5.60	0.00	
	Lamb/mutton	Malawi	362.00	11.00	2.00	2.90	14.00	3.00	4.69	0.00	
	Lamb/mutton	Bangladesh	130.83	4.81	2.85	2.59	7.42	1.43	2.62	0.00	Used vitB12 from FDC
	Lamb/mutton	Colombia	164.88	0.00	9.01	1.07	4.00	1.63	2.34	0.00	
	Lamb/mutton	**Composite**	220.32	2.41	5.93	2.75	10.71	2.16	3.66	0.00	
Other meat	Pork	USDA	241.60	1.80	2.60	0.66	14.60	0.92	2.60	0.00	
	Pork	Kenya	365.00	0.00	4.50	1.00	28.00	1.55	2.36	0.00	
	Pork	Western Africa	353.50	5.50	8.50	0.44	11.50	2.70	2.77	0.00	
	Pork	Laos	226.50	0.00	2.50	0.62	26.50	2.00	1.54	0.00	
	Pork	Colombia	210.75	2.00	1.50	0.77	23.00	1.13	3.08	0.00	
	Pork	**Composite**	241.60	1.80	2.60	0.66	23.00	1.55	2.60	0.00	

Poultry	Chicken	USDA	237.25	30.50	5.75	0.26	13.00	1.13	1.71	0.00	
	Chicken	Western Africa	231.00	28.50	2.50	0.39	13.50	0.75	1.47	0.00	
	Chicken	Laos	157.42	3.24	2.79	0.20	6.78	0.46	0.83	0.00	
	Chicken	Colombia	228.67	26.67	8.33	0.28	13.67	1.03	1.60	0.00	
	Chicken	**Composite**	229.84	27.59	4.27	0.27	13.25	0.89	1.54	0.00	
Milk and Dairy Products	Fresh cow milk	USDA	60.00	32.00	0.00	0.54	123.00	0.00	0.41	0.00	
	Fresh cow milk	Kenya	70.00	41.00	8.00	0.59	119.00	0.10	0.63	0.00	
	Fresh cow milk	Malawi	67.00	44.00	5.00	0.40	120.00	0.10	0.38	0.00	
	Fresh cow milk	Indonesia	67.00	55.00	6.00	0.40	115.00	0.10	0.40	0.00	
	Fresh cow milk	Vietnam	74.00	52.00	5.00	0.44	120.00	0.10	0.40	0.00	
	Fresh cow milk	Colombia	65.00	43.00	5.00	0.36	120.00	0.20	0.40	0.00	
	Fresh cow milk	Mexico	63.00	46.00	5.00	0.45	104.08	2.99	0.46	0.00	
	Fresh cow milk	**Composite**	67.00	44.00	5.00	0.44	120.00	0.10	0.40	0.00	
Milk and Dairy Products	Cooked cow milk	USDA	61.00	46.00	5.00	0.45	113.00	0.03	0.37	0.00	
	Cooked cow milk	Kenya	82.00	47.00	8.00	1.00	140.00	0.10	0.74	0.00	
	Cooked cow milk	Western Africa	64.00	42.00	8.00	0.59	119.00	0.10	0.63	0.00	
	Cooked cow milk	Bangladesh	63.00	32.00	8.50	0.45	103.00	0.10	0.45	0.00	Used vitB12 from FDC
	Cooked cow milk	Thailand	58.00	29.00	4.00	0.25	113.00	0.10	0.34	0.00	
	Cooked cow milk	Colombia	55.00	39.00	5.00	0.45	120.00	0.00	0.40	0.00	
	Cooked cow milk	Mexico	61.00	31.00	5.00	0.20	115.94	3.10	0.60	0.00	
	Cooked cow milk	**Composite**	61.00	39.00	5.00	0.45	115.94	0.10	0.45	0.00	
Milk and Dairy Products	Fresh goat milk	USDA	69.00	57.00	1.00	0.07	134.00	0.05	0.30	0.00	
	Fresh goat milk	Kenya	78.00	32.00	1.00	0.35	180.00	0.10	0.35	0.00	
	Fresh goat milk	Western Africa	83.00	37.00	1.00	0.10	159.00	0.10	0.38	0.00	
	Fresh goat milk	Bangladesh	68.00	32.00	1.00	0.07	152.00	0.20	0.30	0.00	Used vitB12 from FDC
	Fresh goat milk	Colombia	74.00	24.00	1.00	0.07	120.00	0.10	0.40	0.00	
	Fresh goat milk	Mexico	56.00	57.00	1.00	0.07	104.00	0.57	0.30	0.00	
	Fresh goat milk	**Composite**	71.50	34.50	1.00	0.07	143.00	0.10	0.33	0.00	
Milk and Dairy Products	Yoghurt	USDA	61.00	27.00	7.00	0.37	121.00	0.05	0.59	0.00	

	Yoghurt	Kenya	85.00	34.00	0.00	0.20	149.00	0.20	0.34	0.00	
	Yoghurt	Western Africa	82.00	26.00	7.00	0.40	123.00	0.10	0.35	0.00	
	Yoghurt	Vietnam	61.00	26.00	7.00	0.37	120.00	0.10	0.59	0.00	
	Yoghurt	Mexico	77.00	27.00	7.00	0.37	66.60	0.00	0.38	0.00	Used vitB12 from FDC
	Yoghurt	**Composite**	77.00	27.00	7.00	0.37	121.00	0.10	0.38	0.00	
Milk and Dairy Products	Cheese	USDA	353.50	203.50	9.00	1.21	606.00	0.30	3.30	0.00	
	Cheese	Sub-Saharan Africa	388.50	192.00	15.00	1.15	794.00	0.70	2.57	0.00	
	Cheese	South/South-East Asia	363.00	245.00	29.00	0.62	775.00	0.40	3.33	0.00	
	Cheese	Latin America	329.00	211.00	9.50	1.02	653.50	0.60	2.90	0.00	
	Cheese	**Composite**	358.50	212.88	15.63	1.00	707.13	0.50	3.03	0.00	
Organ meats	Beef liver	USDA	181.50	8521.00	254.50	76.22	6.00	6.31	5.23	0.00	
	Beef liver	Kenya	178.00	23677.00	264.00	111.00	7.00	14.60	6.25	0.00	
	Beef liver	Western Africa	176.00	22650.00	270.00	105.00	40.50	12.80	4.17	0.00	
	Beef liver	Laos	121.03	3810.15	196.70	44.59	14.56	8.99	3.37	0.00	
	Beef liver	Colombia	176.50	8645.00	256.50	1.50	6.50	6.80	5.25	0.00	
	Beef liver	**Composite**	176.50	8645.00	256.50	76.22	7.00	8.99	5.23	0.00	
Organ meats	Goat/lamb liver	USDA	229.00	7636.50	236.50	81.10	8.50	9.24	6.76	0.00	
	Goat/lamb liver	Kenya	184.50	28550.00	461.50	89.00	13.00	10.70	5.83	0.00	Values from the African region only
	Goat/lamb liver	Western Africa	206.75	18093.25	349.00	85.05	10.75	9.97	6.30	0.00	
	Goat/lamb liver	**Composite**	206.75	18093.25	349.00	85.05	10.75	9.97	6.30	0.00	
Organ meats	Chicken liver	USDA	173.50	3758.50	573.25	17.44	10.75	11.52	3.76	0.00	
	Chicken liver	Western Africa	157.00	8155.00	1050.00	21.50	7.00	12.35	3.69	0.00	
	Chicken liver	Laos	121.00	3225.50	444.50	14.80	13.00	7.30	3.12	0.00	
	Chicken liver	Colombia	124.69	3217.50	493.92	13.53	10.01	9.15	3.08	0.00	
	Chicken liver	**Composite**	140.85	3492.00	533.59	16.12	10.38	10.34	3.41	0.00	
Organ meats	Pork liver	USDA	165.00	5405.00	163.00	18.67	10.00	17.92	6.72	0.00	
	Pork liver	Laos	113.75	4442.38	135.45	15.10	10.92	13.79	4.58	0.00	

	Pork liver	Colombia	100.86	4796.26	147.76	15.76	8.20	18.15	4.46	0.00	
	Pork liver	**Composite**	113.75	4796.26	147.76	15.76	10.00	17.92	4.58	0.00	Values from the Asian and Latin American region only
Organ meats	Heart	USDA	175.00	3.50	4.50	9.05	10.50	6.11	3.39	0.00	
	Heart	Vietnam	81.12	5.97	25.20	3.24	7.41	4.40	3.12	0.00	
	Heart	Colombia	111.54	3.16	24.54	5.13	7.02	3.82	2.82	0.00	
	Heart	**Composite**	111.54	3.50	24.54	5.13	7.41	4.40	3.12	0.00	Values from the Asian and Latin American region only
Organ meats	Spleen	USDA	149.00	0.00	4.00	5.02	13.00	38.70	3.54	0.00	
	Spleen	Colombia	79.56	0.00	2.65	3.32	7.80	32.97	1.39	0.00	
	Spleen	**Composite**	114.28	0.00	3.33	4.17	10.40	35.84	2.47	0.00	Values from the Latin American region only
Organ meats	Kidney	USDA	151.00	78.00	81.00	24.90	18.00	5.80	3.80	0.00	
	Kidney	Western Africa	132.64	39.00	42.16	14.54	14.20	20.82	3.13	0.00	
	Kidney	Vietnam	56.70	94.50	24.99	4.46	5.60	5.32	1.64	0.00	
	Kidney	Colombia	62.30	59.85	24.99	14.44	9.10	3.33	1.31	0.00	
	Kidney	**Composite**	97.47	68.93	33.58	14.49	11.65	5.56	2.39	0.00	
Fish	Fish (lean and fatty)	USDA	122.00	10.00	15.00	1.64	13.00	0.71	0.50	0.00	
	Fish (lean and fatty)	Sub-Saharan Africa	135.50	18.50	9.00	2.00	76.00	1.25	0.83	0.00	
	Fish (lean and fatty)	South/South-East Asia	122.00	0.00	6.00	1.86	37.44	0.71	0.98	0.00	
	Fish (lean and fatty)	Latin America	114.16	10.94	8.77	1.74	28.02	0.57	0.66	0.00	
	Fish (lean and fatty)	**Composite**	123.42	9.86	9.69	1.81	38.62	0.81	0.74	0.00	
Fish	Small dried fish	Zambia	209.00	540.00			3000.00	8.50		0.00	Values for dried kapenta
	Small dried fish	Mozambique	255.00			12.10	3436.00	33.60	6.40	0.00	Small dried fish
	Small dried fish	Mozambique GAIN/CGIAR analysis					960.00	36.80	11.10	0.00	Small dried fish
	Small dried fish	Tanzania	335.00		28.00	12.00	1700.00	2.50	5.20	0.00	Small dried fish
	Small dried fish	Kabahenda 2011					1556.00	10.70	10.30	0.00	Values from the literature (dried mukene)
	Small dried fish	Kenya	333.00	141.00	46.00	60.00	2790.00	6.90	16.00	0.00	Values for dried dagaa/omena

	Small dried fish	Steiner-Asiedu 1993		186.00			2360.00	10.00	10.00	0.00	Values from the literature (dried kapenta)
	Small dried fish	**Composite**	294.00	186.00	37.00	12.10	2360.00	10.00	10.15	0.00	Values from the African region only; additional values from the literature
Seafood	Crustaceans	USDA	89.00	1.43	18.00	1.00	91.00	0.29	3.81	0.00	
	Crustaceans	Sub-Saharan Africa	114.50	16.00	13.00	2.15	72.50	1.55	1.69	0.00	
	Crustaceans	South/South-East Asia	95.00	4.75	11.33	1.46	82.50	2.10	2.60	0.00	
	Crustaceans	Latin America	55.90	10.24	5.01	0.59	49.40	0.99	0.68	0.00	
	Crustaceans	**Composite**	88.60	8.11	11.84	1.30	73.85	1.23	2.20	0.00	
Seafood	Bivalves	USDA	171.00	91.00	8.00	20.22	33.00	3.86	2.67	0.00	
	Bivalves	Sub-Saharan Africa	98.00	106.00	54.00	48.00	290.00	8.40	1.85	0.00	
	Bivalves	South/South-East Asia	40.50	45.90	6.09	15.05	103.80	4.11	1.13	0.00	
	Bivalves	Latin America	39.30	64.80	8.36	10.71	26.92	2.86	8.70	0.00	
	Bivalves	**Composite**	87.20	76.93	19.11	23.50	113.43	4.81	3.59	0.00	
Fish	Canned fish, without bones	USDA	136.00	20.00	4.00	2.55	17.00	1.39	0.69	0.00	
	Canned fish, without bones	Sub-Saharan Africa	152.50	16.00	6.00	2.25	9.00	1.10	0.74	0.00	
	Canned fish, without bones	Latin America	169.00	5.50	3.50	1.69	11.00	1.25	0.50	0.00	
	Canned fish, without bones	**Composite**	152.50	13.83	4.50	2.16	12.33	1.25	0.64	0.00	Values from the Asian and Latin American region only
Fish	Canned fish, with bones	USDA	182.00	42.50	10.00	3.91	240.00	2.48	1.17	0.00	
	Canned fish, with bones	Sub-Saharan Africa	203.00	49.00	6.00	6.90	241.00	2.40	1.77	0.00	
	Canned fish, with bones	Latin America	218.50	28.00	9.50	6.47	275.00	1.95	1.30	0.00	
	Canned fish, with bones	**Composite**	201.17	39.83	8.50	5.76	252.00	2.28	1.41	0.00	

Supplemental Table 4: second level granular food composition database of aggregated plant-source foods

National /regional FCT	Food group	Food (100 g)	Item Code	Energy (kcal)	Vit A (mcg RAE)	Folate (mcg DFE)	Vit B_{12} (mcg)	Ca (mg)	Iron (mg)	Zinc (mg)	Phytate (mg)	Comments
**USDA**	Pulses	Lentils, mature seeds, cooked, boiled, without salt	16070	116.00	0.00	181.00	0.00	19.00	3.33	1.27	316.40	
		Chickpea, mature seeds, cooked, boiled, without salt	16057	164.00	1.00	172.00	0.00	49.00	2.89	1.53	566.35	
		Cowpea, common, mature seeds, cooked, boiled, without salt	16063	116.00	1.00	143.00	0.00	24.00	2.51	1.29	492.72	
		Beans, kidney, red, mature seeds, cooked, boiled, with salt	16333	127.00	0.00	130.00	0.00	28.00	2.94	1.07	219.00	
		**USDA composite**		121.50	0.50	157.50	0.00	26.00	2.92	1.28	404.56	
**Western Africa**	Pulses	Cowpea, soaked, boiled in different water* (without salt), drained	03_090	122.00	1.00	78.00	0.00	26.00	2.00	1.20	492.72	
		Lentil, soaked, boiled in different water* (without salt), drained	03_087	124.00	1.00	28.00	0.00	24.00	2.10	1.32	316.40	
**Kenya**	Pulses	Beans, broad, dry, water-soaked, boiled in different water, drained (without salt)	3025	109.00	1.00	45.00	0.00	31.00	1.60	1.14	292.50	
		Beans, lima, dry, water-soaked, boiled in different water, drained (without salt)	3035	120.00	0.00	76.00	0.00	23.00	3.40	0.95	566.35	
		Beans, kidney, dry, water-soaked, boiled in different water, drained (without salt)	3031	117.00	0.00	52.00	0.00	36.00	2.10	0.96	219.00	
		**Africa composite**		120.00	1.00	52.00	0.00	26.00	2.10	1.14	316.40	
**Bangladesh**	Pulses	Bengal gram, whole, boiled* (without salt)	02_0012	182.00	2.00	48.00	0.00	94.00	4.00	1.26	566.35	
		Green gram, split, boiled* (without salt)	02_0013	161.00	1.00	32.00	0.00	30.00	2.90	1.13	476.48	

		Grass pea, split, boiled* (without salt)	02_001 4	142.00	2.00	42.00	0.00	24.00	1.90	1.23		Phytate value unavailable
		Lentis, boiled* (without salt)	02_001 5	155.00	1.00	9.00	0.00	12.00	2.20	1.72	316.40	
		**Asia composite**		158.00	1.50	37.00	0.00	27.00	2.55	1.25	476.48	
**Mexico**	Pulses	Bean Black, cooked		132.00	0.00	149.00	0.00	67.00	2.40	1.10		Phytate value unavailable
		Chickpea, cooked		118.00	0.00	65.00	0.00	14.00	1.29	2.20	566.35	
**Colombia**	Pulses	Cargamento red bean, cooked, without salt	T010	161.00	0.00	172.00	0.00	46.00	2.10	1.00		Phytate value unavailable
		Chickpea, cooked, without salt	T018	187.00	2.00	152.00	0.00	51.00	2.60	1.40	566.35	
		Common lentils, cooked, without salt	T025	122.00	5.00	33.00	0.00	14.00	1.70	1.10	316.40	
		Dried peas, cooked, without salt	T002	138.00	8.00	65.00	0.00	25.00	1.50	1.00		Phytate value unavailable
		**Latin America composite**		135.00	1.00	107.00	0.00	35.50	1.90	1.10	566.35	
**USDA**	Whole grains	Wheat, KAMUT khorasan, cooked	169744	132.00	4.00	11.00	0.00	9.00	1.76	1.84	448.80	
		Corn, sweet, white, cooked, boiled, drained, without salt	168539	97.00	0.00	20.00	0.00	2.00	0.55	0.54	426.36	
		Corn, sweet, yellow, cooked, boiled, drained, without salt	169999	96.00	13.00	23.00	0.00	3.00	0.45	0.62	426.36	
		Rice, brown, cooked, no fat added	110163 1	122.00	0.00	9.00	0.00	3.00	0.56	0.71	612.16	
		Barley, cooked, no added fat	110153 4	122.00	0.00	16.00	0.00	11.00	1.32	0.82		Phytate value unavailable
		**USDA composite**		122.00	0.00	16.00	0.00	3.00	0.56	0.71	437.58	
**Kenya**	Whole grains	Wheat, whole, grain, dry, boiled (without salt)	1061	184.00	0.00	22.00	0.00	23.00	2.80	1.43	448.80	
		Wheat, whole, grain, dry, stewed (without salt)	1062	184.00	0.00	26.00	0.00	23.00	2.80	1.51		Phytate value unavailable
		Maize, grain, white variety, whole, dry, boiled, drained (without salt)	1047	111.00	0.00	16.00	0.00	9.00	0.80	0.58	426.36	

		Maize, grain, yellow variety, whole, dry, boiled, drained (without salt)	1049	110.00	6.00	7.00	0.00	4.00	1.30	0.70	342.66	
		Green Maize, white, whole, grain, fresh, stewed (without salt)	1052	203.00	10.00	81.00	0.00	23.00	1.60	2.53	426.36	
**Malawi**	Whole grains	Maize, grain, green, boiled	MW01_ 0040	133.00	0.00	7.00	0.00	7.00	1.10	0.80	342.66	
**Western Africa**	Whole grains	Rice, brown, boiled* (without salt), drained	01_035	138.00	0.00	6.00	0.00	20.00	0.80	0.64	612.16	
		Rice, red native, hulled, boiled* (without salt), drained	01_066	141.00	0.00	6.00	0.00	15.00	1.10	0.57		Phytate value unavailable
		**Africa Composite**		139.50	0.00	11.50	0.00	17.50	1.20	0.75	426.36	
**Banglades h**	Whole grains	Wheat, whole, boiled		193.26	0.00	14.94	0.00	23.03	2.75	1.49	448.80	Used weight yields and nutrient retention factors
**Colombia**	Whole grains	Rice, brown, boiled, WO salt	A011	370.00	0.00	20.00	0.00	28.00	1.80	2.00	612.16	
		Common wheat, boiled		209.55	0.00	16.12	0.00	19.66	2.36	1.65	448.80	Used weight yields and nutrient retention factors
		**LAtina America composite**		289.78	0.00	18.06	0.00	23.83	2.08	1.83	530.48	
**USDA**	Refined grains	Rice, white, medium-grain, cooked, unenriched	168930	130.00	0.00	2.00	0.00	3.00	0.20	0.42	47.47	
		Rice, fried, meatless	110233 6	174.00	22.00	6.00	0.00	11.00	0.46	0.69		Phytate value unavailable
		Barley, pearled, cooked	170285	123.00	0.00	16.00	0.00	11.00	1.33	0.82		Phytate value unavailable
		**USDA composite**		130.00	0.00	6.00	0.00	11.00	0.46	0.69	47.47	
**Kenya**	Refined grains	Rice, white, milled, polished grain, dry, boiled (without salt)	1059	119.00	0.00	2.00	0.00	9.00	0.30	0.44	47.57	
**Western Africa**	Refined grains	Rice, white, polished, boiled* (without salt), drained	01_069	118.00	0.00	2.00	0.00	3.00	0.30	0.40	47.57	
		**SSA Composite**		118.50	0.00	2.00	0.00	6.00	0.30	0.42	47.57	
**Banglades h**	Refined grains	Rice, white, sunned, aromatic, boiled* (without salt)	01_004 0	110.00	0.00	2.00	0.00	6.00	0.40	0.33	47.57	

**Laos**	Refined grains	Rice, steamed, white	1019	131.00	0.00	4.00	0.00	7.00	0.40	0.60	23.94	
		Rice, white, fried	1025	370.00	0.00	7.00	0.00	11.00	1.60	1.20		Phytate value unavailable
		**Asia composite**		131.00	0.00	4.00	0.00	7.00	0.40	0.60	35.76	
**Colombia**	Refined grains	White rice, polished, boiled, WO salt	A009	161.00	0.00	2.00	0.00	10.00	0.20	0.70	47.57	
		Barley, pearled, boiled		154.00	0.00	6.44	0.00	14.40	1.03	0.80		Phytate value unavailable; used weight yields and nutrient retention factors
**Mexico**	Refined grains	Barley, pearl, boiled		140.80	0.36	6.44	0.00	11.60	1.00	0.81		Phytate value unavailable; used weight yields and nutrient retention factors
		**LAtina America composite**		154.00	0.00	6.44	0.00	11.60	1.00	0.80	47.57	
**USDA**	Unrefined cereal products	Bread, french or vienna, whole wheat	174091	239.00	0.00	131.00	0.00	42.00	0.75	1.16	49.00	
		Bread, rye	172684	259.00	0.00	151.00	0.00	73.00	2.83	1.14	77.00	
		Pasta, whole wheat, cooked	168910	149.00	0.00	21.00	0.00	13.00	1.72	1.34		Phytate value unavailable
		Cereals, oats, regular and quick, unenriched, cooked with water (includes boiling and microwaving), without salt	173905	71.00	0.00	6.00	0.00	9.00	0.90	1.00	809.90	
		Cornmeal mush, no added fat	110156 4	65.00	2.00	37.00	0.00	4.00	0.65	0.11	198.98	
		**USDA composite**		149.00	0.00	37.00	0.00	13.00	0.90	1.14	137.99	
**Kenya**	Unrefined cereal products	Bread, brown	1005	245.00	0.00	37.00	0.00	56.00	2.60	1.30	49.00	
		Whole maize porridge	15001	52.00	7.00	7.00	0.00	25.00	0.30	0.33	198.98	
		Oat porridge	15123	86.00	11.00	3.00	0.00	39.00	0.40	0.45	809.90	
**Malawi**	Unrefined cereal products	Bread, wheat, brown, homemade	MW01_ 0003	362.00	35.00	56.00	0.00	13.00	4.30	0.30	49.00	
		Maize thick porridge, white, whole flour	MW01_ 0034	86.00	0.00	4.00	0.00	8.00	0.90	0.39	198.98	

		**Africa composite**		86.00	7.00	7.00	0.00	25.00	0.90	0.39	198.98	
**ASEAN**	Unrefined cereal products	Bread, wholewheat	AAA19	273.00	0.00		0.00	83.00	1.90		49.00	
**Colombia**	Unrefined cereal products	Wholewheat bread, baked	A069	279.00	0.00	36.00	0.00	50.00	2.70	1.50	49.00	
**Mexico**	Unrefined cereal products	Rye, whole bread		223.50	0.00	134.00	0.00	68.00	4.01	1.48	77.00	
		Wheat, whole, bread loaf		263.00	0.00	50.00	0.00	41.00	4.65	2.00	49.00	
		**LAtina America composite**		263.00	0.00	50.00	0.00	50.00	4.01	1.50	49.00	
**USDA**	Refined cereal products	Bread, pita, white, unenriched	172816	275.00	0.00	24.00	0.00	86.00	1.40	0.84	49.26	
		Pasta, cooked, unenriched, WO added salt	168928	158.00	0.00	7.00	0.00	7.00	0.50	0.51		Phytate value unavailable
		Rice noodles, cooked	168914	108.00	0.00	1.00	0.00	4.00	0.14	0.25		Phytate value unavailable
		**USDA composite**		158.00	0.00	7.00	0.00	7.00	0.50	0.51	49.26	
**Kenya**	Refined cereal products	Bread, white	1007	249.00	0.00	28.00	0.00	37.00	1.70	0.80	49.26	
		Pasta, macaroni, plain, dry, imported, boiled, drained (without salt)	1065	150.00	0.00	5.00	0.00	10.00	0.60	0.25		Phytate value unavailable
**Malawi**	Refined cereal products	Rice porridge	MW01_ 0052	42.00	4.00	1.00	0.00	8.00	0.00	0.19	20.00	
		Bread, wheat, white, homemade	MW01_ 0005	270.00	0.00	29.00	0.00	28.00	1.20	0.59	49.26	
		Macaroni/Spaghetti, unenriched, cooked	MW01_ 0026	124.00	0.00	0.00	0.00	8.00	0.40	0.48		Phytate value unavailable
		Maize thick porridge, degermed-dehulled flour	MW01_ 0031	72.00	0.00	1.00	0.00	5.00	0.20	0.10		Phytate value unavailable
		**Africa Composite**		137.00	0.00	3.00	0.00	9.00	0.50	0.37	49.26	

**Bangladesh**	Refined cereal products	Vermicelli, wheat, boiled, WO salt	01_0043	151.00	0.00	5.00	0.00	11.00	0.90	0.57	164.74	
**Vietnam**	Refined cereal products	Bread, French style	1012	249.00	0.00	33.00	0.00	28.00	2.00	0.85	49.26	
**Laos**	Refined cereal products	Porridge, white rice, boiled	16132	59.00	0.00	1.00	0.00	2.00	0.13	0.19	20.00	
		**Asia composite**		151.00	0.00	5.00	0.00	11.00	0.90	0.57	49.26	
**Colombia**	Refined cereal products	bread, French style, baked	A060	308.00	0.00	62.00	0.00	32.00	1.90	0.80	49.26	
		Pasta, unenriched, boiled, WO salt	A073	143.00	0.00	7.00	0.00	7.00	0.50	0.50		Phytate value unavailable
		**LAtina America composite**		225.50	0.00	34.50	0.00	19.50	1.20	0.65	49.26	
**USDA**	Nuts	Peanuts, all types, dry-roasted, WO salt	173806	587.00	0.00	97.00	0.00	58.00	1.58	2.77	2008.00	
		Peanuts, all types, raw	172430	567.00	0.00	240.00	0.00	92.00	4.58	3.27	582.00	
		Nuts, cashew nuts, raw	170162	553.00	0.00	25.00	0.00	37.00	6.68	5.78	290.00	
		Nuts, cashew nuts, dry roasted, without salt added	170571	574.00	0.00	69.00	0.00	45.00	6.00	5.60	1229.00	
		Nuts, almonds	170567	579.00	0.00	44.00	0.00	269.00	3.71	3.12	964.00	
		Nuts, almonds, dry roasted, without salt added	170158	598.00	0.00	55.00	0.00	268.00	3.73	3.31	2111.00	
		Nuts, pistachio nuts, raw	170184	560.00	26.00	51.00	0.00	105.00	3.92	2.20	808.00	
		Nuts, walnuts, english	170187	654.00	1.00	98.00	0.00	98.00	2.91	3.09	611.00	
		Peanuts, all types, cooked, boiled, with salt	174260	318.00	0.00	75.00	0.00	55.00	1.01	1.83	505.00	
		Nuts, pine nuts, dried	170591	673.00	1.00	34.00	0.00	16.00	5.53	6.45	200.00	
		**USDA composite**		576.50	0.00	62.00	0.00	75.00	3.83	3.20	709.50	
**Kenya**	Nuts	Nut, almond, with or without skin, raw, unsalted	10007	612.00	1.00	37.00	0.00	249.00	4.10	3.44	964.00	

		Nut, cashew, dry, raw, unsalted	10008	595.00	1.00	24.00	0.00	33.00	4.90	5.36	290.00
		Nut, ground nut, with skin, unsalted, dry, raw	10009	593.00	0.00	110.00	0.00	117.0 0	5.50	2.24	582.00
		Nut, macadamia, raw, unsalted	10010	696.00	0.00	114.00	0.00	44.00	2.20	1.30	150.00
		Nut, pistachio, raw, unsalted	10011	591.00	63.00	16.00	0.00	116.0 0	4.80	2.07	808.00
**Malawi**	Nuts	Groundnut, dry	MW02_ 0014	597.00	0.00	110.00	0.00	45.00	2.10	2.32	582.00
		Groundnut, dry, boiled	MW02_ 0015	324.00	0.00	60.00	0.00	41.00	2.10	1.40	505.00
		**Africa Composite**		595.00	0.00	60.00	0.00	45.00	4.10	2.24	582.00
**Banglades h**	Nuts	Cashew nuts, raw	06_000 2	595.00	1.00	25.00	0.00	50.00	5.00	5.78	290.00
		Groundnuts/peanut, raw	06_000 7	585.00	0.00	175.00	0.00	76.00	2.90	3.39	582.00
		Pistachio nuts, dried	06_001 3	574.00	16.00	51.00	0.00	117.0 0	5.30	2.20	808.00
		Walnuts	06_001 6	684.00	1.00	98.00	0.00	100.0 0	4.80	3.09	611.00
**Vietnam**	Nuts	Peanut, dried	3017	573.00	1.00	240.00	0.00	68.00	2.20	1.90	582.00
		**Asia composite**		585.00	1.00	98.00	0.00	76.00	4.80	3.09	582.00
**Colombia**	Nuts	Peanut, with skin, raw	T028	619.00	0.00	240.00	0.00	62.00	3.30	3.30	582.00
		Peanut, with skin, roasted, WO salt	T029	650.00	0.00	68.00	0.00	53.00	1.80	3.30	2008.00
**Mexico**	Nuts	Peanut, raw		519.00	0.00	240.00	0.00	49.50	5.28	2.88	582.00
		Peanut, roasted		610.00	0.00	145.00	0.00	102.1 7	3.49	1.93	2008.00
		Pistachio nut seed		631.00	21.00	51.00	0.00	105.0 0	3.92	2.20	808.00
		Pine nut		673.00	1.00	34.00	0.00	13.00	5.20	6.45	200.00
		Almond		526.00	0.00	25.00	0.00	24.00	1.80	5.78	964.00
		**Latin America composite**		619.00	0.00	68.00	0.00	53.00	3.49	3.30	808.00

**USDA**	Seeds	Seeds, pumpkin and squash seed kernels, dried	170556	559.00	1.00	58.00	0.00	46.00	8.82	7.81	24.25	
		Seeds, pumpkin and squash seed kernels, roasted, without salt	170557	574.00	0.00	57.00	0.00	52.00	8.07	7.64	56.10	
		Seeds, flaxseed	169414	534.00	0.00	87.00	0.00	255.00	5.73	4.34	1859.00	
		Seeds, sesame seeds, whole, dried	170150	573.00	0.00	97.00	0.00	975.00	14.60	7.75	1525.00	
		Seeds, sesame seeds, whole, roasted and toasted	170151	565.00	0.00	98.00	0.00	989.00	14.80	7.16		Phytate value unavailable
		Seeds, sunflower seed kernels, dried	170562	584.00	3.00	227.00	0.00	78.00	5.25	5.00	423.00	
		Seeds, sunflower seed kernels, toasted, without salt	170154	619.00	0.00	238.00	0.00	57.00	6.81	5.30		Phytate value unavailable
		Seeds, lotus seeds, dried	170149	332.00	3.00	104.00	0.00	163.00	3.53	1.05	339.48	
		**USDA composite**		569.00	0.00	97.50	0.00	120.50	7.44	6.23	381.24	
**Kenya**	Seeds	Seed, pumpkin, hulled & dried, unsalted	10013	575.00	19.00	56.00	0.00	50.00	5.50	7.24	24.25	
		Seed, sesame (sim sim) unsalted, dry, raw	10014	602.00	0.00	103.00	0.00	1430.00	9.70	1.72	1525.00	
		Seed, sunflower, unsalted, dry, raw	10015	595.00	1.00	224.00	0.00	98.00	5.00	6.00	423.00	
		Jack fruit, seed, mature, dry, raw	10005	156.00	0.00	148.00	0.00	84.00	5.90	1.56	141.00	
**Western Africa**	Seeds	Pumpkin seed, kernel only, dried, raw	06_038	567.00	2.00	58.00	0.00	62.00	8.30	8.89	24.25	
		Sesame seed, whole, dried, raw	06_015	601.00	1.00	100.00	0.00	777.00	11.20	2.10	1525.00	
		**Africa composite**		585.00	1.00	101.50	0.00	91.00	7.10	4.05	282.00	
**Bangladesh**	Seeds	Sunflower seeds, dried	06_0001	552.00	2.00	227.00	0.00	78.00	5.30	5.00	423.00	
		Linseeds, tisi, raw	06_0009	500.00	0.00	87.00	0.00	255.00	5.70	4.34	1859.00	

		Pumpkin seeds, dried	06_0014	580.00	1.00	58.00	0.00	45.00	8.10	7.21	24.25	
		Sesame seeds, whole, dried	06_0015	563.00	0.00	97.00	0.00	969.00	10.50	7.70	1525.00	
		Lotus seeds, dried	06_0010	339.00	3.00	104.00	0.00	147.00	5.60	1.05	339.48	
**Vietnam**	Seeds	Lotus seed, dried	4041	334.00	3.00	104.00	0.00	89.00	6.40	1.05	339.48	
		Sesame seeds, dried	3020	568.00	1.00	97.00	0.00	975.00	14.60	7.75	1525.00	
		**Asia composite**		552.00	1.00	97.00	0.00	147.00	6.40	5.00	423.00	
**Colombia**	Seeds	Sesame seed, raw	T001	648.00	2.00	97.00	0.00	975.00	12.70	6.80	1525.00	
**Mexico**	Seeds	Sesame seed		540.00	0.00	97.00	0.00	1038.00	9.24	7.75	1525.00	
		Sunflower seed, raw		611.00	3.00	227.00	0.00	78.00	5.45	6.09	423.00	
		**LAtin America composite**		611.00	2.00	97.00	0.00	975.00	9.24	6.80	1525.00	
**USDA**	Stachy roots, tubers and plantains	Potatoes, baked, flesh and skin, without salt	170093	93.00	1.00	28.00	0.00	15.00	1.08	0.36		Phytate value unavailable
		Potatoes, boiled, cooked in skin, flesh, without salt	170438	87.00	0.00	10.00	0.00	5.00	0.31	0.30	4.82	
		Cassava, cooked	1103070	191.00	13.00	24.00	0.00	17.00	0.28	0.36	60.00	
		Sweet potato, cooked, baked in skin, flesh, without salt	168483	90.00	961.00	6.00	0.00	38.00	0.69	0.32		Phytate value unavailable
		Sweet potato, boiled, no added fat	1103247	76.00	784.00	6.00	0.00	27.00	0.72	0.20	12.00	
		Yam, cooked, boiled, drained, or baked, without salt	170072	116.00	6.00	16.00	0.00	14.00	0.52	0.20	50.00	
		Plantains, green, fried	168216	309.00	60.00	15.00	0.00	4.00	0.67	0.23	0.57	
		Plantains, yellow, baked	169131	155.00	45.00	53.00	0.00	3.00	0.28	0.21		Phytate value unavailable
		**USDA composite**		104.50	29.00	15.50	0.00	14.50	0.60	0.27	12.00	

**Kenya**	Starchy roots, tubers and plantains	Arrowroot, peeled, boiled, drained (without salt)	2020	109.00	0.00	0.00	0.00	6.00	1.00	0.86		Phytate value unavailable
		Cassava, root, white, peeled, boiled, drained (without salt)	2023	148.00	1.00	15.00	0.00	27.00	0.70	0.26	60.00	
		Cassava, root, yellow, peeled, boiled, drained (without salt)	2024	107.00	49.00	15.00	0.00	15.00	0.50	0.26	60.00	
		Potato, Irish (English), white variety, peeled, boiled, drained (without salt)	2025	105.00	0.00	34.00	0.00	8.00	1.30	0.29	4.82	
		Potato, Irish (English), white variety, peeled, steamed (without salt)	2026	107.00	0.00	33.00	0.00	8.00	1.30	0.31		Phytate value unavailable
		Sweet potato, brown skin, peeled, boiled, drained (without salt)	2027	76.90	8.00	10.00	0.00	21.00	0.40	0.25	12.00	
		Sweet potato, pink skin, boiled, drained (without salt)	2029	83.00	1.00	9.00	0.00	13.00	0.90	0.13	12.00	
		Yam, white, boiled, drained (without salt)	2030	118.00	0.00	10.00	0.00	16.00	0.80	0.26	50.00	
**Malawi**	Starchy roots, tubers and plantains	Cassava, tuber, boiled	MW01_0010	146.00	1.00	14.00	0.00	26.00	0.60	0.60	60.00	
		Cocoyam, tuber, boiled	MW01_0012	145.00		22.00	0.00	11.00	0.60	0.36	37.00	
		Sweet potato, white-fleshed, without skin, boiled	MW01_0066	97.00	4.00	8.00	0.00	19.00	0.40	0.20	12.00	
		Plantain, green, boiled	MW01_0048	124.00	38.00	13.00	0.00	6.00	0.80	0.10	6.23	
		Cassava, tuber, steamed		144.00	0.81	12.96	0.00	65.84	1.15	0.92		Phytate value unavailable; weight yields and retention factors used
		**Africa composite**		109.00	1.00	13.00	0.00	15.00	0.80	0.26	24.50	

**Banglades h**	starchy roots, tubers and plantains	Colocasia/Taro, boiled* (without salt)	05_001 1	115.00	4.00	16.00	0.00	40.00	0.80	0.23	18.10	No other Asian FCTs have values for cooked tubers
		Potato, Diamond, boiled* (without salt)	05_001 2	67.00	2.00	11.00	0.00	14.00	0.60	0.72	4.82	
		Sweet potato, Komola Sundori, orange flesh, boiled* (without salt)	05_001 6	98.00	692.00	7.00	0.00	31.00	0.70	0.27	12.00	
		Sweet potato, skin purple, flesh pale-yellow, boiled* (without salt)	05_001 5	106.00	3.00	7.00	0.00	33.00	1.40	0.37	12.00	
		Yam, tuber, boiled* (without salt)	05_002 0	109.00	3.00	16.00	0.00	29.00	0.90	0.34	50.00	
		Sweet potato, Komola Sundori, orange flesh, steamed		87.30	582.39	5.94	0.00	25.65	0.46	0.24		Phytate value unavailable; weight yields and retention factors used
		Sweet potato, skin purple, flesh pale-yellow, steamed (without skin)		93.60	2.43	5.94	0.00	27.36	1.07	0.32		Phytate value unavailable; weight yields and retention factors used
		**Asia composite**		98.00	3.00	7.00	0.00	29.00	0.80	0.32	12.00	
**Colombia**	Starchy roots, tubers and plantains	Potato, waxy variety, sabanera, with skin, boiled, WO salt	B064	99.00	0.00	11.00	0.00	8.00	0.80	0.30	4.82	
		Potato, floury variety, pastusa, with skin, boiled, WO salt	B073	85.00	0.00	9.00	0.00	6.00	1.00	0.40	4.82	
		Plantain harton, ripe, boiled, WO salt	B088	130.00	56.00	26.00	0.00	4.00	0.50	0.20	0.00	
		Plantain harton, ripe, fried, sliced, WO salt	B090	312.00	61.00	24.00	0.00	3.00	0.80	0.30	0.57	
		Plantain harton, green, boiled, WO salt	B091	133.00	45.00		0.00	7.00	0.50	0.20	1.00	
		Plantain harton, green, fried, sliced, WO salt	B093	353.00	60.00	15.00	0.00	4.00	0.70	0.20	0.57	

		White cassava, without skin, boiled, WO salt	B106	157.00	1.00	11.00	0.00	16.00	0.50	0.20	60.00	
		White cassava, WO skin, steamed		143.10	0.81	14.58	0.00	13.68	0.23	0.24		Phytate value unavailable; weight yields and retention factors used
		Potato, waxy variety, sabanera, with skin, baked		64.80	0.00	9.72	0.00	9.72	0.65	0.24		Phytate value unavailable; weight yields and retention factors used
**Mexico**	Starchy roots, tubers and plantains	Sweet potato, cooked		78.00	787.00	6.00	0.00	27.00	2.49	0.20	12.00	
		**Latin America composite**		131.50	23.00	11.00	0.00	7.50	0.68	0.22	2.91	
**USDA**	DGLV	Spinach, raw	1103136	23.00	469.00	194.00	0.00	99.00	2.71	0.53	12.01	
		Kale, cooked, boiled, drained, without salt	169355	44.00	146.00	65.00	0.00	150.0 0	0.84	0.27	127.00	
		romaine lettuce, raw	1103106	15.00	370.00	38.00	0.00	36.00	0.86	0.18	3.00	
		Chard, swiss, cooked, boiled, drained, without salt	170401	20.00	306.00	9.00	0.00	58.00	2.26	0.33		Phytate value unavailable
		Pumpkin leaves, cooked, boiled, drained, without salt	168447	21.00	80.00	25.00	0.00	43.00	3.20	0.20	34.00	
		**USDA composite**		21.00	306.00	38.00	0.00	58.00	2.26	0.27	23.01	
**Kenya**	DGLV	Kale (sukuma wiki), boiled, drained (without salt)	4074	28.00	152.00	30.00	0.00	364.0 0	2.00	0.34	127.00	
		Amaranth, leaves, picked, stewed (without salt)	4040	45.00	362.00	55.00	0.00	346.0 0	8.30	1.14	38.00	
		Spinach, leaves, stewed (without salt)	4089	33.00	179.00	58.00	0.00	161.0 0	2.10	0.74		Phytate value unavailable
		Pumpkin, leaves, boiled, drained (without salt)	4082	35.00	121.00	17.00	0.00	347.0 0	4.00	0.64	34.00	
**Malawi**	DGLV	Cabbage, Chinese, boiled, Brassica chinensis, (Chayinizi)	MW04_ 0003	19.00	8.00	13.00	0.00	84.00	0.30	0.60	5.00	
		Leaves, pumpkin, boiled, Cucurbita maxima, (Nkhwani wowilitsa)	MW04_ 00018	47.00	485.00	47.00	0.00	142.0 0	2.20	0.70	34.00	

		Leaves, cassava, boiled, Manihot esculenta, (Ntapasha/Chigwada chowilitsa)	MW04_ 00013	85.00	211.00	48.00	0.00	178.0 0	3.40	1.20	28.54	
		Leaves, amaranth, boiled, Ama - ranthus thunbergii, (Bonongwe wowilitsa)	MW04_ 00010	57.00	283.00	52.00	0.00	372.0 0	6.10	1.30	38.00	
		**Africa Composite**		40.00	195.00	47.50	0.00	262.0 0	2.80	0.72	34.00	
**Banglades h**	DGLV	Indian spinach, boiled* (without salt)	04_003 6	33.00	201.00	92.00	0.00	140.0 0	2.20	0.35		Phytate value unavailable
		Slender amaranth leaves, boiled* (without salt)	04_003 4	55.00	1030.00	50.00	0.00	255.0 0	4.80	1.06	38.00	
		Cabbage, boiled* (without salt)	03_003 4	27.00	5.00	24.00	0.00	35.00	0.50	0.34	5.00	Used phytate for boiled Chinese cabbage
		Spinach, raw	04_002 5	26.00	409.00	194.00	0.00	90.00	2.20	0.90	12.01	
**Indonesia**	DGLV	Spinach, cooked	IDD011	27.00	119.00	48.00	0.00	150.0 0	0.50	0.30		Phytate value unavailable
		Cabbage, raw	IDD123	32.00	5.00	20.00	0.00	46.00	0.50	0.30	1.01	
		Cassava leaf, stir-fried	IDD076	68.00	456.00	12.00	0.00	166.0 0	1.30	2.60	28.54	
		Chinese cabbage, stir-fried	IDD151	32.00	331.00	0.00	0.00	220.0 0	2.90	0.20	5.00	
		**Asia Composite**		32.00	266.00	36.00	0.00	145.0 0	1.75	0.35	8.51	
**Mexico**	DGLV	Spinach, cooked		23.00	524.00	146.00	0.00	136.0 0	3.57	7.22		Phytate value unavailable
		Broccoli, cooked		35.00	77.00	108.00	0.00	40.00	0.67	5.13		Phytate value unavailable
		Watercress, raw		24.50	160.00	9.00	0.00	127.6 6	2.51	0.11		limit of detection (LOD) for phytate
**Colombia**	DGLV	Chard, leaves, raw	B003	38.00	413.00	165.00	0.00	49.00	1.80	0.40		Phytate value unavailable
		Spinach, cooked, without salt	B043	35.00	479.00	146.00	0.00	132.0 0	1.90	0.70		Phytate value unavailable
		Lettuce, raw	B059	18.00	10.00	53.00	0.00	21.00	0.90	0.30	3.00	

		Watercress, raw	B024	27.00	240.00	80.00	0.00	195.00	1.60	0.20		limit of detection (LOD) for phytate
		**Latina America composite**		27.00	240.00	108.00	0.00	127.66	1.80	0.40	3.00	Phytate: LOD (limit of detection)
**USDA**	VitA-rich fruits and veg, excl DGLV	Carrots, raw	170393	41.00	835.00	19.00	0.00	33.00	0.30	0.24	19.60	
		Carrots, cooked, boiled, drained, without salt	170394	35.00	852.00	14.00	0.00	30.00	0.34	0.20		Phytate value unavailable
		Peppers, sweet, red, raw	170108	26.00	157.00	46.00	0.00	7.00	0.43	0.25	17.68	
		Peppers, sweet, yellow, raw	169383	27.00	10.00	26.00	0.00	11.00	0.46	0.17	14.81	
		Peppers, sweet, red, sauteed	168550	133.00	138.00	2.00	0.00	7.00	0.47	0.15		Phytate value unavailable
		Pumpkin, cooked, boiled, drained, without salt	168449	20.00	288.00	9.00	0.00	15.00	0.57	0.23		Phytate value unavailable
		Squash, winter, butternut, cooked, baked, WO salt	169296	40.00	558.00	19.00	0.00	41.00	0.60	0.13		Phytate value unavailable
		Mangos, raw	169910	60.00	54.00	43.00	0.00	11.00	0.16	0.09	25.00	
		Papayas, raw	169926	43.00	47.00	37.00	0.00	20.00	0.25	0.08	25.44	
		Melon, cantaloupe, raw	169092	34.00	169.00	21.00	0.00	9.00	0.21	0.18	20.00	
		Guavas, common, raw	173044	68.00	31.00	49.00	0.00	18.00	0.26	0.23	45.89	
		Passion fruit, (granadilla), purple, raw	169108	97.00	64.00	14.00	0.00	12.00	1.60	0.10	81.40	
		**USDA composite**		40.50	147.50	20.00	0.00	13.50	0.39	0.18	22.50	
**Kenya**	VitA-rich fruits and veg, excl DGLV	Capsicum (sweet pepper), red, raw	4009	26.00	21.00	52.00	0.00	4.00	0.30	0.20	17.68	
		Capsicum (sweet pepper), red, grilled (without salt and fat)	4056	28.00	20.00	39.00	0.00	4.00	0.30	0.22		Phytate value unavailable
		Capsicum (sweet pepper), yellow, raw	4010	27.00	14.00	30.00	0.00	19.00	1.00	0.00	14.81	
		Capsicum (sweet pepper), yellow, baked (without salt)	4060	22.00	13.00	22.00	0.00	20.00	0.70	0.27		phytate value unavailable
		Carrot, peeled, raw	4011	30.00	589.00	18.00	0.00	33.00	0.60	0.14	19.60	

		Carrot, peeled, boiled, drained (without salt)	4061	32.00	564.00	10.00	0.00	34.00	0.50	0.11		phytate value unavailable
		Pumpkin, flesh, yellow w/o seeds, boiled, drained (without salt)	4082	35.00	123.00	27.00	0.00	18.00	0.20	0.17		phytate value unavailable
		Squash, butternut, peeled, flesh, boiled, drained (without salt)	4092	48.00	162.00	18.00	0.00	19.00	0.80	0.16		phytate value unavailable
		Squash, butternut, peeled, flesh, baked (without salt)	4094	51.00	169.00	26.00	0.00	21.00	1.20	0.23		phytate value unavailable
		Mango, ripe, raw	5019	64.00	92.00	54.00	0.00	14.00	0.20	0.14	25.00	
		Papaya, yellow skin, peeled, raw	5024	36.00	85.00	37.00	0.00	21.00	0.60	0.20	25.44	
		Passion fruit, dark purple skin, raw	5025	75.00	64.00	7.00	0.00	10.00	0.60	0.80	81.40	
		Guava, pink-fleshed, raw	5011	48.00	22.00	16.00	0.00	20.00	0.40	0.26	45.89	
**Malawi**	VitA-rich fruits and veg, excl DGLV	Pumpkin, boiled, Cucurbita maxima	MW04_0034	60.00	189.00	9.00	0.00	35.00	2.00	0.20		phytate value unavailable
		Mango, ripe	MW05_0016	66.00	123.00	34.00	0.00	114.00	1.70	0.54	25.00	
		Guavas, Psidium guajava	MW05_0008	71.00	5.00	19.00	0.00	7.00	0.40	0.20	45.89	
		**Africa composite**		42.00	88.50	24.00	0.00	19.50	0.60	0.20	25.00	
**Bangladesh**	VitA-rich fruits and veg, excl DGLV	Carrot, raw	03_0008	34.00	329.00	15.00	0.00	26.00	0.40	0.07	19.60	
		Carrot, boiled (without salt)	03_0035	43.00	364.00	9.00	0.00	34.00	0.50	0.07		phytate value unavailable
		Pumpkin, boiled (without salt)	03_0043	29.00	554.00	14.00	0.00	79.00	1.10	0.14		phytate value unavailable
		Mango, Fazli, orange flesh, ripe, raw	08_0025	70.00	292.00	71.00	0.00	14.00	0.50	0.87	25.00	
		Mango, Langra, yellow flesh, ripe, raw	08_0026	82.00	25.00	71.00	0.00	13.00	0.20	0.60	25.00	

		Papaya, ripe, raw	08_0035	33.00	60.00	58.00	0.00	29.00	0.30	0.17	25.44	
		Melon, Futi, orange flesh, ripe, raw	08_0027	17.00	105.00	21.00	0.00	17.00	0.20	0.06	20.00	
**Vietnam**	VitA-rich fruits and veg, excl DGLV	Carrots, raw	4007	39.00	1686.00	19.00	0.00	43.00	0.80	1.11	19.60	
		Papaya, ripe	5017	35.00	55.00	38.00	0.00	40.00	1.40	0.10	25.44	
		Guava, common	5040	33.00	31.00	49.00	0.00	10.00	0.90	0.50	45.89	
		Mango, common; India mango (ripe)	5055	69.00	38.00	14.00	0.00	10.00	0.40	0.56	25.00	
		**Asia composite**		35.00	105.00	21.00	0.00	26.00	0.50	0.17	25.00	
**Colombia**	VitA-rich fruits and veg, excl DGLV	Carrot, peeled, boiled, without salt	B109	43.00	1667.00	14.00	0.00	30.00	0.40	0.20		phytate value unavailable
		Carrot, peeled, raw	B110	47.00	1318.00	22.00	0.00	27.00	0.40	0.20	19.60	
		Red pepper, raw	B080	35.00	184.00	35.00	0.00	7.00	0.40	0.20	17.68	
		Mango, common, raw	C051	79.00	140.00	39.00	0.00	15.00	0.50	0.10	25.00	
		Melon, raw	C060	25.00	68.00	21.00	0.00	11.00	0.40	0.10	20.00	
		Guava, ripe, raw	C031	71.00	31.00	49.00	0.00	13.00	0.30	0.30	45.89	
		Papaya, ripe, raw	C065	40.00	235.00	37.00	0.00	24.00	0.30	0.10	25.44	
**Mexico**	VitA-rich fruits and veg, excl DGLV	Carrot		51.00	841.00	19.00	0.00	26.50	1.47	0.24	19.60	
		Carrot, boiled		35.00	860.00	14.00	0.00	30.00	0.34	2.00		phytate value unavailable
		Pumpkin, boiled		15.00	56.00	28.00	0.00	18.00	0.37	0.33		phytate value unavailable
		Mango		47.00	54.00	43.00	0.00	20.50	1.03	0.69	25.00	
		Papaya		33.00	47.00	38.00	0.00	17.00	0.46	0.44	25.44	
		Melon, cantaloupe		28.00	169.00	21.00	0.00	23.00	1.40	0.18	20.00	
		Guava		46.00	31.00	49.00	0.00	33.50	0.85	0.23	45.89	
		**Latin America composite**		41.50	154.50	31.50	0.00	21.75	0.40	0.22	25.00	

**USDA**	Other fruits, excl vitA-rich fruits	Bananas, raw	173944	89.00	3.00	20.00	0.00	5.00	0.26	0.15	22.00	
		Apple, raw	1102644	52.00	3.00	3.00	0.00	6.00	0.12	0.04	0.57	
		Avocados, raw, all commercial varieties	171705	160.00	7.00	81.00	0.00	12.00	0.55	0.64	11.00	
		Oranges, raw, all commercial varieties	169097	47.00	11.00	30.00	0.00	40.00	0.10	0.07	10.00	
		Pineapple, raw, all varieties	169124	50.00	3.00	18.00	0.00	13.00	0.29	0.12	8.59	
		Nuts, coconut meat, raw	170169	354.00	0.00	26.00	0.00	14.00	2.43	1.10	136.00	
		Watermelon, raw		30.00	28.00	3.00	0.00	7.00	0.24	0.10	10.00	
		Grapes, red or green (European type, such as Thompson seedless), raw	174683	69.00	3.00	2.00	0.00	10.00	0.36	0.07	0.05	
		**USDA composite**		60.50	3.00	19.00	0.00	11.00	0.28	0.11	10.00	
**Kenya**	Other fruits, excl vitA-rich fruits	Apple, red, unpeeled, raw	5002	57.00	1.00	5.00	0.00	6.00	0.30	0.07	0.57	
		Avocado, ripe, raw	5003	185.00	2.00	67.00	0.00	19.00	1.00	0.39	11.00	
		Banana, cavendish, raw	5004	95.00	5.00	22.00	0.00	10.00	0.30	0.36	22.00	
		Orange, pulp, raw	5023	42.00	5.00	52.00	0.00	23.00	0.20	0.05	10.00	
		Pineapple, raw	5030	41.00	3.00	32.00	0.00	16.00	0.40	0.00	8.59	
		Coconut, fresh, mature fruit, flesh	10002	423.00	0.00	14.00	0.00	18.00	2.30	0.73	136.00	
**Malawi**	Other fruits, excl vitA-rich fruits	Banana, fried	MW05_0003	213.00	2.00	10.00	0.00	8.00	0.80	0.16		Phytate value unavailable
		Banana, white fleshed, raw, peeled	MW05_0004	109.00	4.00	20.00	0.00	9.00	0.30	0.20	22.00	
		Apple, average, raw	MW05_0001	63.00	3.00	1.00	0.00	2.00	0.30	0.09	0.57	
		Avocado, raw, peeled	MW05_0002	152.00	6.00	33.00	0.00	7.00	0.70	0.16	11.00	

		Pineapple, raw, peeled	MW05_0021	58.00	5.00	16.00	0.00	4.00	0.30	0.03	8.59	
		Orange, raw, peeled	MW05_0018	54.00	4.00	30.00	0.00	30.00	0.30	0.22	10.00	
		Watermelon, raw, peeled	MW05_0025	33.00	11.00	4.00	0.00	3.00	0.10	0.09	10.00	
		**Africa composite**		63.00	4.00	20.00	0.00	9.00	0.30	0.16	10.00	
**Bangladesh**	Other fruits, excl vitA-rich fruits	Apple, with skin, raw	08_002	62.00	3.00	3.00	0.00	6.00	0.10	0.04	0.57	
		Banana, Sagar, ripe, raw	08_004	95.00	2.00	20.00	0.00	11.00	0.30	0.24	22.00	
		Orange, sweet, ripe, raw	08_0032	49.00	11.00	46.00	0.00	31.00	0.10	0.07	10.00	
		Lemon, Kagoji, raw	08_0022	56.00	4.00	17.00	0.00	65.00	0.30	0.07	22.00	
		Watermelon, ripe, raw	08_0042	22.00	29.00	3.00	0.00	12.00	0.40	0.15	10.00	
		Grapes, green, raw	08_0014	94.00	3.00	8.00	0.00	22.00	0.50	0.07	0.05	
**Laos**	Other fruits, excl vitA-rich fruits	Apple, pink, fresh	5005	63.00	7.00	3.00	0.00	7.00	0.20	0.04	0.57	
		Orange, sweet, fresh	5003	52.00	3.00	30.00	0.00	33.00	0.40	0.70	10.00	
		Banana, ripe, yellow	5024	105.00	8.00	21.00	0.00	11.00	0.40	0.10	22.00	
		Banana, ripe, yellow, boiled	5034	105.00	6.00	13.00	0.00	10.00	0.40	0.10		phytate value unavailable
		**Asia composite**		62.50	5.00	15.00	0.00	11.50	0.35	0.09	10.00	
**Colombia**	Other fruits, excl vitA-rich fruits	Banana, common, raw	C010	101.00	22.00	21.00	0.00	8.00	0.90	0.20	22.00	
		Lemon, raw	C044	44.00	1.00	11.00	0.00	19.00	0.50	0.10	22.00	
		Coconut, raw	C019	402.00	0.00	26.00	0.00	7.00	1.30	0.80	136.00	
		Apple, common, raw	C054	72.00	3.00	1.00	0.00	16.00	0.30	0.00	0.57	
		Orange, raw	C062	41.00	2.00	30.00	0.00	33.00	1.30	0.50	10.00	
		Avocado Hass, raw	C001	221.00	7.00	89.00	0.00	8.00	0.50	0.70	11.00	

**Mexico**	Other fruits, excl vitA-rich fruits	Apple		52.00	3.00	3.00	0.00	6.17	0.41	0.58	0.57	
		Banana		89.00	3.00	20.00	0.00	5.00	0.26	0.54	22.00	
		Pineapple		33.00	3.00	18.00	0.00	13.00	0.47	0.52	8.59	
		Orange		36.50	11.00	30.00	0.00	39.50	0.23	0.51	10.00	
		Coconut		353.00	0.00	26.00	0.00	14.00	3.10	1.10	136.00	
		**Latin America composite**		72.00	3.00	21.00	0.00	13.00	0.50	0.52	11.00	
**USDA**	Other vegetables, excl DGLV and vitA-rich veg	Tomatoes, red, ripe, raw, year round average	170457	18.00	42.00	15.00	0.00	10.00	0.27	0.17	2.39	
		Tomatoes, red, ripe, cooked	170050	18.00	24.00	13.00	0.00	11.00	0.68	0.14		Phytate value unavailable
		Peppers, sweet, green, raw	170427	20.00	18.00	10.00	0.00	10.00	0.34	0.13	15.96	
		Peppers, sweet, green, cooked, boiled, drained, without salt	170428	28.00	23.00	16.00	0.00	9.00	0.46	0.12		Phytate value unavailable
		Okra, raw	169260	33.00	36.00	60.00	0.00	82.00	0.62	0.58	5.20	
		Okra, cooked, boiled, drained, without salt	169261	22.00	14.00	46.00	0.00	77.00	0.28	0.43	13.00	
		Eggplant, cooked, boiled, drained, without salt	169229	35.00	2.00	14.00	0.00	6.00	0.25	0.12		Phytate value unavailable
		Cauliflower, cooked, boiled, drained, without salt	170397	23.00	1.00	44.00	0.00	16.00	0.32	0.17		Phytate value unavailable
		Beans, snap, green, cooked, boiled, drained, without salt	164191	35.00	32.00	33.00	0.00	44.00	0.65	0.25		Phytate value unavailable
		Cucumber, with peel, raw	168409	15.00	5.00	7.00	0.00	16.00	0.28	0.20	17.52	
		**USDA composite**		22.50	20.50	15.50	0.00	13.50	0.33	0.17	13.00	
**Kenya**	Other vegetables, excl DGLV and vitA-rich veg	Tomato, red, ripe, raw	4036	22.00	26.00	25.00	0.00	8.00	0.90	0.37	2.39	
		Tomato, red, ripe, boiled, drained (without salt)	4098	28.00	30.00	16.00	0.00	10.00	0.80	0.35		Phytate value unavailable

		Capsicum (sweet peper), green, raw	4008	23.00	27.00	8.00	0.00	8.00	0.50	0.18	15.96	
		Capsicum (sweet peper), green, grilled (without salt and fat)	4054	24.00	26.00	6.00	0.00	9.00	0.60	0.19		Phytate value unavailable
		Okra, fresh, raw	4026	32.00	9.00	88.00	0.00	81.00	0.80	0.60	5.20	
		Okra, fresh, boiled, drained (without salt)	4081	29.00	8.00	40.00	0.00	70.00	0.50	0.41	13.00	
		Eggplant / Brinjal, different varieties, whole edible, boiled, drained (without salt)	4068	29.00	12.00	16.00	0.00	19.00	0.30	0.17		Phytate value unavailable
		Eggplant / Brinjal, different varieties, whole edible, stewed (without salt)	4069	33.00	14.00	25.00	0.00	23.00	0.50	0.26		Phytate value unavailable
		Cucumber, green, unpeeled, raw	4016	11.00	0.00	7.00	0.00	20.00	0.70	0.18	17.52	
**Malawi**	Other vegetables, excl DGLV and vitA-rich veg	Green pepper, raw	MW04_0009	24.00	36.00	5.00	0.00	7.00	0.90	0.24	15.96	
		Okra, raw	MW04_0030	61.00	41.00	138.00	0.00	104.00	1.30	0.94	5.20	
		**Africa composite**		28.00	26.00	16.00	0.00	19.00	0.70	0.26	13.00	
**Bangladesh**	Other vegetables, excl DGLV and vitA-rich veg	Tomato, red, ripe,raw	03_0031	16.00	9.00	15.00	0.00	13.00	0.20	0.41	2.39	
		Tomato, red, ripe, boiled* (without salt)	03_0045	30.00	16.00	15.00	0.00	24.00	0.40	0.66		Phytate value unavailable
		Okra/ladies finger, raw	03_0023	39.00	19.00	60.00	0.00	93.00	0.90	0.34	5.20	
		Okra/ladies finger, boiled* (without salt)	03_0040	32.00	15.00	26.00	0.00	72.00	0.70	0.23	13.00	
		Brinjal, purple, long, boiled* (without salt)	03_0033	26.00	4.00	20.00	0.00	24.00	0.40	0.52		Phytate value unavailable

		Cauliflower, boiled* (without salt)	03_0036	28.00	1.00	32.00	0.00	36.00	0.80	0.37		Phytate value unavailable
		Cucumber, peeled, raw	03_0012	17.00	4.00	14.00	0.00	13.00	0.60	0.17	17.52	
**Laos**	Other vegetables, excl DGLV and vitA-rich veg	Cucumber, fresh	4030	23.00	5.00	9.00	0.00	20.00	0.40	0.20	17.52	
		Tomato, fresh	4043	25.00	44.00	9.00	0.00	17.00	0.90	0.20	2.39	
		**Asia composite**		26.00	9.00	15.00	0.00	24.00	0.60	0.34	9.10	
**Mexico**	Other vegetables, excl DGLV and vitA-rich veg	Eggplant, boiled		35.00	2.00	14.00	0.00	6.00	0.25	3.70		Phytate value unavailable
		Okra		49.00	36.00	60.00	0.00	85.00	1.61	0.58	5.20	
		Green Bean, boiled		35.00	35.00	33.00	0.00	44.00	0.65	3.02		Phytate value unavailable
**Colombia**	Other vegetables, excl DGLV and vitA-rich veg	Eggplant, with skin, boiled, WO salt	B022	37.00	6.00	14.00	0.00	7.00	0.20	0.10		Phytate value unavailable
		Cauliflower, boiled, without salt	B038	41.00	1.00	36.00	0.00	16.00	0.30	0.30		Phytate value unavailable
		Okra, raw	B062	45.00	47.00	72.00	0.00	82.00	0.60	0.60	5.20	
		Cucumber, raw	B077	14.00	2.00	14.00	0.00	18.00	0.30	0.10	17.52	
		Green pepper, raw	B081	28.00	20.00	10.00	0.00	11.00	0.40	0.10	15.96	
		Tomato, raw	B103	23.00	62.00	23.00	0.00	9.00	0.50	0.10	2.39	
		Tomato, heated, without salt	B104	25.00	25.00	20.00	0.00	10.00	0.40	0.10		Phytate value unavailable
		**Latin America composite**		35.00	22.50	21.50	0.00	13.50	0.40	0.20	5.20	

Supplemental Table 5: second level granular food composition database of individual plant-source foods

National/regional FCT	Food group	Food (100 g)	Item Code	Energy (kcal)	Vit A (mcg RAE)	Folate (mcg DFE)	Vit B_{12} (mcg)	Ca (mg)	Iron (mg)	Zinc (mg)	Phytate (mg)	Comments
**USDA**	Traditional grains	Millet, cooked	168871	119.00	0.00	19.00	0.00	3.00	0.63	0.91	200.00	
**Kenya**	Traditional grains	Millet, bulrush, whole, grain, dry, boiled, drained (without salt)	1053	148.00	1.00	47.00	0.00	15.00	2.60	1.64	200.00	
		Millet, bulrush, whole, grain, dry, stewed, drained (without salt)	1054	148.00	1.00	54.00	0.00	15.00	2.60	1.73	200.00	
		**Millet composite**		148.00	1.00	50.50	0.00	15.00	2.60	1.69	200.00	
**Western Africa**	traditional grains	Pearl millet, whole grains, boiled* (without salt), drained	01_033	152.00	0.00	47.00	0.00	10.00	6.30	1.02	200.00	
**Bangladesh**	Traditional grains	Pearl millet, whole-grain, stewed		145.42	0.00	28.33	0.00	17.50	3.33	1.29	200.00	Used weight yields and nutrient retention factors
		Pearl millet, whole-grain, boiled		145.42	0.00	24.79	0.00	17.50	3.33	1.23	200.00	Used weight yields and nutrient retention factors
		**Millet composite**		145.42	0.00	26.56	0.00	17.50	3.33	1.26	200.00	
**Colombia**	Traditional grains	Millet, grain, stewed		158.75	0.00	28.33	0.00	3.33	1.25	0.71	200.00	Used weight yields and nutrient retention factors
		Millet, grain, boiled		158.75	0.00	24.79	0.00	3.33	1.25	0.67	200.00	Used weight yields and nutrient retention factors
		**Millet composite**		158.75	0.00	26.56	0.00	3.33	1.25	0.69	200.00	
**Kenya**	Traditional grains	Sorghum, whole, grain, red, dried, boiled, drained (without salt)	1057	140.00	0.00	19.00	0.00	6.00	1.40	0.78	272.00	
		Sorghum, whole, grain, red, dried, stewed, drained (without salt)	1058	140.00	0.00	21.00	0.00	6.00	1.40	0.82		Phytate value unavailable

		Sorghum, whole, grain white, dry, boiled, drained (without salt)	1055	142.00	1.00	19.00	0.00	9.00	2.70	0.67	272.00	
		Sorghum, whole, grain white, dry, stewed, drained (without salt)	1056	142.00	1.00	21.00	0.00	9.00	2.70	0.70		
		**Sorghum composite**		141.00	0.50	20.00	0.00	7.50	2.05	0.74	272.00	
**Western Africa**	Traditional grains	Sorghum, whole grains, red, boiled* (without salt), drained	01_070	143.00	0.00	19.00	0.00	10.00	4.00	0.78	272.00	
		Sorghum, whole grains, white, boiled* (without salt), drained	01_071	144.00	0.00	19.00	0.00	9.00	2.20	0.82	272.00	
		**Sorghum composite**		143.50	0.00	19.00	0.00	9.50	3.10	0.80	272.00	
**USDA**	Traditional grains	Teff, cooked	168918	101.00	0.00	18.00	0.00	49.00	2.05	1.11		Phytate value unavailable
**Western Africa**	Traditional grains	Teff, whole grains, boiled* (WO salt), drained	01_185	197.00	0.00	65.00	0.00	101.00	4.30	1.94		Phytate value unavailable
**Nyachoti 2021**	Traditional grains	Average white & red teff, stewed (Main Ethiopian Rift Valley)						42.78	5.34	0.84		Values from the literature; used weight yields and nutrient retention factors
**Western Africa**	Traditional grains	Fonio, white, whole grains, boiled* (WO salt), drained	01_003	139.00	0.00	36.00	0.00	17.00	3.50	1.10	110.00	Value for iron from the 2012 FCT
		Fonio, black, whole grains, boiled* (WO salt), drained	01_049	138.00	1.00	36.00	0.00	21.00	4.10	1.50		Value for iron from the 2012 FCT; phytate value unavailable
		**Fonio composite**		138.50	0.50	36.00	0.00	19.00	3.80	1.30	110.00	

**Koroch & Ballogou, 2013**	Traditional grains	Average fonio, stewed						4.81	1.82	0.9		Values from the literature; used weight yields and nutrient retention factors
**USDA**	Traditional grains	Quinoa, cooked	168917	120.00	0.00	42.00	0.00	17.00	1.49	1.09	554.14	
**Colombia**	Traditional grains	Quinoa, stewed		109.94	0.00	43.04	0.00	16.08	2.46	0.94	554.14	Used weight yields and nutrient retention factors

Supplemental Table 6: second level granular food composition database of aggregated animal-source foods

National/ regional FCT	Food group	Food (100 g)	Item Code	Energy (kcal)	Vit A (mcg RAE)	Folate (mcg DFE)	Vit B_{12} (mcg)	Ca (mg)	Iron (mg)	Zinc (mg)	Phytate (mg)	Comments
**USDA**	Milk and dairy products	Cheese, cheddar	1098007	408.00	316.00	21.00	1.06	707.00	0.16	3.67	0.00	
		Cottage cheese, farmer's	1098046	143.00	83.00	11.00	0.41	78.00	0.07	0.38	0.00	
		Cheese, mozzarella, whole milk	170845	299.00	179.00	7.00	2.28	505.00	0.44	2.92	0.00	
		Cheese, parmesan, hard	14108020	421.00	228.00	6.00	1.35	884.00	0.45	4.33	0.00	
		**USDA composite**		353.50	203.50	9.00	1.21	606.00	0.30	3.30	0.00	
**Kenya**	Milk and dairy products	Cheese, cheddar, regular fat	6003	401.00	174.00	52.00	1.90	800.00	0.30	3.55	0.00	
		Cheese, cottage (cow milk), plain, whole	6005	121.00	35.00	3.00	0.60	89.00	0.10	0.24	0.00	
		Cheese, cottage, Milk, Cow, Sour	6006	122.00	37.00	12.00	0.43	112.00	0.70	0.40	0.00	
**Western Africa**	milk and dairy products	Cheese, cheddar, from cow's milk	10_006	397.00	263.00	18.00	1.80	788.00	0.70	3.93	0.00	
		Cheese, Gouda, from cow's milk	10_008	380.00	210.00	21.00	1.70	806.00	0.80	3.74	0.00	
		Cheese, hard type, from goat's milk	10_007	447.00	486.00	4.00	0.12	895.00	1.90	1.59	0.00	
		**Africa composite**		388.50	192.00	15.00	1.15	794.00	0.70	2.57	0.00	
**Bangladesh**	Milk and dairy products	Cheese, cottage, 25% fat	12_0002	346.00	205.00	40.00	0.41	790.00	0.30	3.55	0.00	Used vitB12 from USDA
**Vietnam**	Milk and dairy products	Cheese, cheddar	10009	380.00	285.00	18.00	0.83	760.00	0.50	3.11	0.00	
		**Asia composite**		363.00	245.00	29.00	0.62	775.00	0.40	3.33	0.00	
**Colombia**	Milk and dairy products	Fresh cheese, semi-hard, semi-fat, farmer's	G017	301.00	420.00	5.00	0.30	586.00	0.40	2.90	0.00	

		Fresh cheese, semi-hard, semi-fat, type mozzarella	G019	296.00	241.00	7.00	2.28	517.00	0.20	2.90	0.00
		Cheese type cottage, soft, skimmed, with cream	G022	101.00	48.00	12.00	0.58	60.00	0.10	0.40	0.00
		Aged cheese, hard, semi-fat, type parmesan	G024	389.00	181.00	7.00	1.20	1184.00	0.80	2.80	0.00
**Mexico**	Milk and dairy products	Aged cheese (queso añejo)		492.00	64.00	1.00	1.38	860.00	2.42	2.94	0.00
		Cheese, cheddar		403.00	265.00	18.00	0.83	721.00	0.90	3.11	0.00
		Cheese, edam		357.00	243.00	16.00	1.54	829.00	1.74	3.75	0.00
		Cheese, type cottage		112.18	37.00	12.00	0.04	123.97	0.07	0.37	0.00
		**Latin America composite**		329.00	211.00	9.50	1.02	653.50	0.60	2.90	0.00
**USDA**	Fresh fish	Sea bass, baked or broiled, no fat added	1098987	122.00	52.00	6.00	0.34	13.00	0.37	0.50	0.00
		Sea bass, steamed or poached	1098991	122.00	46.00	5.00	0.32	13.00	0.36	0.50	0.00
		Tilapia, baked or broiled, no fat added	1099064	121.00	0.00	27.00	1.79	13.00	0.71	0.42	0.00
		Tilapia, steamed or poached	1099076	121.00	0.00	24.00	1.69	13.00	0.70	0.42	0.00
		Herring, baked or broiled, no fat added	1098878	199.00	30.00	12.00	14.63	72.00	1.39	1.25	0.00
		Carp, baked or broiled, no fat added	1098776	160.00	10.00	17.00	1.64	52.00	1.56	1.86	0.00
		Carp, steamed or poached	1098780	160.00	10.00	15.00	1.64	52.00	1.56	1.86	0.00
		**USDA composite**		122.00	10.00	15.00	1.64	13.00	0.71	0.50	0.00

**Kenya**	Fresh fish	Cod, fillet, steamed (without salt)	8015	90.00	2.00	8.00	2.00	18.00	0.20	0.45	0.00	
		Cod, fillet, grilled (without salt and fat)	8016	98.00	2.00	10.00	2.00	20.00	0.20	0.49	0.00	
		Herring stock, steamed (without salt)	8022	137.00	34.00	7.00	12.00	80.00	1.10	0.97	0.00	
		Herring stock, grilled (without salt)	8023	148.00	36.00	8.00	14.00	87.00	1.30	1.05	0.00	
**Malawi**	Fresh fish	Fish, catfish, fresh, fried	MW03_0021	207.00	38.00	18.00	1.90	44.00	1.70	0.84	0.00	
		Fish, tilapia, fresh, grilled	MW03_0030	146.00	54.00	11.00	0.10	117.00	4.00	0.00	0.00	
**Western Africa**	Fresh fish	Perch, Nile, fillet, grilled* (without salt or fat)	09_034	134.00	20.00	8.00	1.70	161.00	1.20	0.81	0.00	
		Perch, Nile, fillet, steamed* (without salt)	09_033	111.00	16.00	6.00	1.30	134.00	1.00	0.67	0.00	
		Sardine, fillet, grilled* (without salt or fat)	09_036	154.00	17.00	26.00	11.00	72.00	2.10	2.22	0.00	
		Sardine, fillet, steamed* (without salt)	09_035	128.00	14.00	19.00	8.00	60.00	1.70	1.85	0.00	
		**Africa composite**		135.50	18.50	9.00	2.00	76.00	1.25	0.83	0.00	
**Laos**	Fresh fish	Short bodied mackarel fried	7028	236.00	26.00	2.00	4.84	114.00	2.40	1.00	0.00	
		Short bodied mackarel roasted	7031	122.00	27.00	1.00	5.70	62.00	1.40	0.60	0.00	
		Nile tilapia roasted	7027	128.00	0.00	6.00	1.86	14.00	0.69	0.41	0.00	
**Vietnam**	Fresh fish	Carp, bighead, boiled		103.68	0.00	7.56	0.88	37.44	0.71	1.07	0.00	Used weight yields and nutrient retention factors
		Tilapia, stewed		66.00	0.00	6.93	0.81	32.80	0.28	0.98	0.00	Used weight yields and nutrient retention factors
		**Asia composite**		122.00	0.00	6.00	1.86	37.44	0.71	0.98	0.00	

**Mexico**	Fresh fish	Carp, broiled		116.00	10.00	17.00	1.47	102.60	1.59	1.90	0.00	Used weight yields and nutrient retention factors
		Sea bass, stewed		51.48	11.88	5.54	0.58	6.60	0.08	0.24	0.00	
**Colombia**	Fresh fish	Catfish, lean, whole, boiled, WO salt	E006	138.00	1.00	12.00	2.00	15.00	0.50	0.60	0.00	
		Herring, boiled		112.32	16.13	5.04	7.80	41.04	0.63	0.72	0.00	Used weight yields and nutrient retention factors
		**Latin America composite**		114.16	10.94	8.77	1.74	28.02	0.57	0.66	0.00	
**USDA**	Crustaceans	Crustaceans, crab, blue, cooked, moist heat	174205	83.00	1.00	51.00	3.33	91.00	0.50	3.81	0.00	
		Crustaceans, lobster, northern, cooked, moist heat	174209	89.00	1.43	11.00	1.00	96.00	0.29	4.05	0.00	
		Shrimp, steamed or boiled	1099155	91.00	62.00	18.00	0.85	69.00	0.24	1.24	0.00	
		**USDA composite**		89.00	1.43	18.00	1.00	91.00	0.29	3.81	0.00	
**Kenya**	Crustaceans	Prawns, fresh, steamed (without salt)	8031	119.00	16.00	11.00	2.00	71.00	1.40	1.72	0.00	
		Prawns, fresh, grilled (without salt and fat)	8032	115.00	16.00	13.00	2.00	69.00	1.70	1.66	0.00	
**Western Africa**	Crustaceans	Shrimp, penaeid, flesh, grilled* (without salt or fat)	09_106	114.00	16.00	13.00	2.20	76.00	1.60	1.51	0.00	
		Shrimp, penaeid, flesh, steamed* (without salt)	09_105	118.00	16.00	11.00	2.10	79.00	1.50	1.56	0.00	
		Crab, flesh (body and claw), grilled* (without salt or fat)	09_097	108.00	78.00	41.00	9.40	73.00	1.60	4.97	0.00	
		Crab, flesh (body and claw), steamed* (without salt)	09_096	107.00	74.00	35.00	8.30	72.00	1.50	4.91	0.00	

		**Africa composite**		114.50	16.00	13.00	2.15	72.50	1.55	1.69	0.00	
**Vietnam**	Crustaceans	Shrimp, sea water, boiled		53.30	11.70	1.37	0.60	51.35	0.83	0.72	0.00	Used weight yields and nutrient retention factors
		Shrimp, fields river, boiled		58.50	8.78	8.65	0.58	47.45	1.14	0.63	0.00	Used weight yields and nutrient retention factors
		**Asia composite**		55.90	10.24	5.01	0.59	49.40	0.99	0.68	0.00	
**Colombia**	Crustaceans	Shrimp, boiled, WO salt	E016	103.00	68.00	4.00	1.49	90.00	3.00	1.60	0.00	
		Blue crab, boiled, WO salt	E022	87.00	2.00	44.00	9.00	89.00	0.70	3.60	0.00	
**Mexico**	Crustaceans	Lobster, cooked		106.00	1.43	11.00	1.43	76.00	3.86	4.05	0.00	
		Crawfish, boiled		39.96	7.49	11.66	0.93	13.88	1.20	0.56	0.00	Used weight yields and nutrient retention factors
		**Latin America composite**		95.00	4.75	11.33	1.46	82.50	2.10	2.60	0.00	
**USDA**	Bivalves	Mollusks, mussel, blue, cooked, moist heat	174217	172.00	91.00	76.00	24.00	33.00	6.72	2.67	0.00	
		Clams, steamed or boiled	1099108	171.00	161.00	7.00	20.22	78.00	2.90	1.02	0.00	
		Ostrich, oyster, raw	174487	125.00	0.00	8.00	4.91	6.00	3.86	3.59	0.00	
		**USDA composite**		171.00	91.00	8.00	20.22	33.00	3.86	2.67	0.00	
**Western Africa**	Bivalves	Venus clams, flesh, steamed* (without salt)	09_094	98.00	106.00	54.00	48.00	290.00	8.40	1.85	0.00	
**Vietnam**	Bivalves	Mussels, fresh water, boiled		22.80	48.60	6.72	23.71	15.00	0.72	0.82	0.00	Used weight yields and nutrient retention factors
**Indonesia**	Bivalves	Clams, boiled		58.20	43.20	5.46	6.38	192.60	7.49	1.44	0.00	Used weight yields and nutrient retention factors
		**Asia composite**		40.50	45.90	6.09	15.05	103.80	4.11	1.13	0.00	
**Colombia**	Bivalves	Clams, fresh water, boiled		39.60	48.60	6.72	5.41	25.80	0.82	0.78	0.00	Used weight yields and nutrient retention factors
**Mexio**	Bivalves	Oyster, raw		39.00	81.00	10.00	16.00	28.04	4.90	16.62	0.00	
		**Latin America composite**		39.30	64.80	8.36	10.71	26.92	2.86	8.70	0.00	

**USDA**	Canned fish, without bones	Fish, tuna, light, canned in oil, drained solids	173708	198.00	23.00	5.00	2.20	13.00	1.39	0.90	0.00
		Fish, tuna, light, canned in water, drained solids	173709	86.00	17.00	4.00	2.55	17.00	1.63	0.69	0.00
		Fish, Salmon, pink, canned, drained solids, without skin and bones	173724	136.00	20.00	4.00	4.96	60.00	0.57	0.65	0.00
		**USDA composite**		136.00	20.00	4.00	2.55	17.00	1.39	0.69	0.00
**Western Africa**	Canned fish, without bones	Tuna, canned in water, drained	09_110	119.00	15.00	9.00	2.20	11.00	1.30	0.76	0.00
		Tuna, canned in oil, drained	09_111	186.00	17.00	3.00	2.30	7.00	0.90	0.72	0.00
		**Africa composite**		152.50	16.00	6.00	2.25	9.00	1.10	0.74	0.00
**Colombia**	Canned fish, without bones	Tuna, canned in oil	E003	211.00	5.00	5.00	2.20	8.00	1.50	0.50	0.00
		Tuna, canned in water	E004	127.00	6.00	2.00	1.17	14.00	1.00	0.50	0.00
		**Latin America composite**		169.00	5.50	3.50	1.69	11.00	1.25	0.50	0.00
**USDA**	Canned fish, with bones	Fish, salmon, sockeye, canned, without salt, drained solids with bone	174226	153.00	53.00	10.00	0.30	239.00	1.06	1.02	0.00
		Fish, anchovy, european, canned in oil, drained solids	174183	210.00	12.00	13.00	0.88	232.00	4.63	2.44	0.00
		Fish, mackerel, jack, canned, drained solids	175121	156.00	130.00	5.00	6.94	241.00	2.04	1.02	0.00
		Fish, sardine, Atlantic, canned in oil, drained solids with bone	175139	208.00	32.00	10.00	8.94	382.00	2.92	1.31	0.00

		**USDA composite**		182.00	42.50	10.00	3.91	240.00	2.48	1.17	0.00
**Western Africa**	Canned fish, with bones	Anchovy, canned in oil, drained	09_012	203.00	12.00	13.00	0.88	232.00	4.60	2.44	0.00
		Mackerel, jack, canned in oil, drained	09_109	149.00	130.00	5.00	6.90	241.00	2.00	1.02	0.00
		Sardine, canned in oil, drained, with bones	09_037	239.00	49.00	6.00	9.00	421.00	2.40	1.77	0.00
		**Africa composite**		203.00	49.00	6.00	6.90	241.00	2.40	1.77	0.00
**Colombia**	Canned fish, with bones	Salmon, canned in oil	E037	212.00	28.00	11.00	4.00	200.00	1.00	1.00	0.00
		Sardines, canned in oil	E039	225.00	28.00	8.00	8.94	350.00	2.90	1.60	0.00
		**Latin America composite**		218.50	28.00	9.50	6.47	275.00	1.95	1.30	0.00

Supplemental Table 7: second level granular food composition database of individual animal-source foods

National/ regional FCT	Food group	Food (100 g)	Item Code	Energy (kcal)	Vit A (mcg RAE)	Folate (mcg DFE)	Vit B_{12} (mcg)	Ca (mg)	Iron (mg)	Zinc (mg)	Phytate (mg)	Comments
**USDA**	Ruminant meat	Beef, stew meat, cooked, lean and fat eaten	1098202	236.00	0.00	9.00	2.60	5.00	3.13	4.30	0.00	
		Beef, brisket, whole, separable lean and fat, trimmed to 1/8" fat, all grades, cooked, braised	168665	331.00	0.00	7.00	2.40	7.00	2.46	5.77	0.00	
		Beef, rib eye steak, boneless, lip-on, separable lean and fat, trimmed to 1/8" fat, all grades, cooked, grilled	173392	291.00	8.00	6.00	2.10	11.00	2.24	5.91	0.00	
		Beef steak, fried, lean and fat eaten	1098174	235.00	3.00	7.00	1.94	17.00	2.26	5.21	0.00	
		**Beef composite**		273.25	2.75	7.25	2.26	10.00	2.52	5.30	0.00	
**Western Africa**	Ruminant meat	Beef meat, moderately fat, ca. 20% fat, boiled* (without salt), drained	07_012	320.00	21.00	11.00	1.10	19.00	2.10	5.66	0.00	
		Beef meat, moderately fat, ca. 20% fat, grilled* (without salt or fat)	07_013	286.00	18.00	12.00	1.30	18.00	1.60	4.72	0.00	
		**Beef composite**		303.00	19.50	11.50	1.20	18.50	1.85	5.19	0.00	
**Laos**	Ruminant meat	Beef, grilled	06018	190.00	3.00	3.00	2.20	9.00	4.90	7.60	0.00	
		Beef, blanched	06034	150.00	2.00	1.00	1.10	9.00	3.00	5.00	0.00	
		**Beef composite**		170.00	2.50	2.00	1.65	9.00	3.95	6.30	0.00	
**Colombia**	Ruminant meat	Beef, steak, roasted/grilled, without salt	F104	175.00	4.00	5.00	2.32	5.00	3.10	4.30	0.00	
		Beef, tenderloin, boiled/braised, without salt	F114	218.00	0.00	9.00	2.74	5.00	3.10	6.20	0.00	
		Beef, round, fried, without salt	F095	176.00	1.00	5.00	2.00	10.00	3.00	5.20	0.00	
		**Beef composite**		189.67	1.67	6.33	2.35	6.67	3.07	5.23	0.00	

**USDA**	Ruminant meat	Goat, boiled	1098358	142.00	0.00	5.00	1.18	17.00	3.70	5.23	0.00	
		Game meat, goat, cooked, roasted	175304	143.00	0.00	5.00	1.19	17.00	3.73	5.27	0.00	
		Goat, fried	1098359	153.00	0.00	5.00	1.16	17.00	3.64	5.14	0.00	
		**Goat composite**		146.00	0.00	5.00	1.18	17.00	3.69	5.21	0.00	
**Western Africa**	Ruminant meat	Goat meat, moderately fat, ca. 10% fat, boiled* (without salt), drained	07_047	208.00	11.00	3.00	1.60	32.00	3.30	5.05	0.00	
		Goat meat, moderately fat, ca. 10% fat, grilled* (without salt or fat)	07_048	204.00	10.00	3.00	2.00	39.00	3.10	4.91	0.00	
		**Goat composite**		206.00	10.50	3.00	1.80	35.50	3.20	4.98	0.00	
**Bangladesh**	Ruminant meat	Goat meat, lean, boiled		70.80	0.00	1.80	1.18	5.76	1.68	2.40	0.00	Used weight yields and nutrient retention factors; used VitB12 values from USDA
		Goat meat, lean, roasted		86.73	0.00	2.94	1.19	7.94	1.96	2.94	0.00	Used weight yields and nutrient retention factors; used VitB12 values from USDA
		**Goat composite**		78.77	0.00	2.37	1.19	6.85	1.82	2.67	0.00	
**Colombia**	Ruminant meat	Goat meat, boiled/braised, without salt	F004	137.00	0.00	5.00	1.19	17.00	3.70	5.30	0.00	
**USDA**	Ruminant meat	Lamb, roast, cooked, lean and fat eaten	1098355	265.00	0.00	20.00	2.59	16.00	1.96	4.78	0.00	
		Lamb, foreshank, separable lean and fat, trimmed to 1/4" fat, choice, cooked, braised	172482	243.00	0.00	17.00	2.28	20.00	2.14	7.69	0.00	
		Lamb, rib, separable lean and fat, trimmed to 1/4'' fat, choice, cooked, broiled	174322	361.00	0.00	14.00	2.54	19.00	1.88	4.00	0.00	
		Mutton, cooked, roasted	167634	234.00		0.00	4.44	10.00	4.76	5.93	0.00	Value for vitamin A is missing
		**Lamb/Mutton composite**		275.75	0.00	12.75	2.96	16.25	2.69	5.60	0.00	
**Malawi**	Ruminant meat	Lamb, meat, roast	MW03_0056	362.00	11.00	2.00	2.90	14.00	3.00	4.69	0.00	

**Bangladesh**	Ruminant meat	Lamb/mutton, meat, moderately fat, roasted		144.06	5.29	3.53	2.59	8.60	1.54	2.88	0.00	Used weight yields and nutrient retention factors; used VitB12 values from USDA
		Lamb/mutton, meat, moderately fat, boiled		117.60	4.32	2.16		6.24	1.32	2.35	0.00	Used weight yields and nutrient retention factors
		**Lamb/Mutton composite**		130.83	4.81	2.85	2.59	7.42	1.43	2.62	0.00	
**Colombia**	Ruminant meat	Lamb/mutton, moderately fat, roasted		181.55	0.00	11.17	1.26	4.63	1.75	2.57	0.00	Used weight yields and nutrient retention factors
		Lamb/mutton, moderately fat, boiled		148.20	0.00	6.84	0.88	3.36	1.50	2.10	0.00	Used weight yields and nutrient retention factors
		**Lamb/Mutton composite**		164.88	0.00	9.01	1.07	4.00	1.63	2.34	0.00	
**USDA**	Poultry	Chicken, stewing, meat and skin, cooked, stewed	172401	285.00	39.00	5.00	0.23	13.00	1.37	1.77	0.00	
		Chicken, broilers or fryers, meat and skin, cooked, roasted	171450	239.00	48.00	5.00	0.30	15.00	1.26	1.94	0.00	
		Chicken breast, grilled, WO sauce, skin eaten	1098456	206.00	17.00	6.00	0.17	7.00	0.53	0.87	0.00	
		Chicken, broilers or fryers, meat only, cooked, fried		219.00	18.00	7.00	0.34	17.00	1.35	2.24	0.00	
		**Chicken composite**		237.25	30.50	5.75	0.26	13.00	1.13	1.71	0.00	
**Western Africa**	Poultry	Chicken, dark meat with skin, boiled* (without salt), drained	07_031	268.00	36.00	5.00	0.19	12.00	1.00	2.08	0.00	
		Chicken, light meat with skin, grilled* (without salt or fat)	07_038	194.00	21.00	0.00	0.58	15.00	0.50	0.86	0.00	
		**Chicken composite**		231.00	28.50	2.50	0.39	13.50	0.75	1.47	0.00	
**Laos**	Poultry	Chicken, boiled	6053	193.00	5.00	4.00	0.24	7.00	0.60	1.00	0.00	
		Chicken, roasted		121.83	1.47	1.57	0.15	6.55	0.31	0.66	0.00	Used weight yields and nutrient retention factors
		**Chicken composite**		157.42	3.24	2.79	0.20	6.78	0.46	0.83	0.00	
**Colombia**	Poultry	Chicken leg, with skin, fried, WO salt	F092	265.00	43.00	13.00	0.29	11.00	1.40	2.20	0.00	
		Chicken breast, with skin, boiled, WO salt	F084	174.00	25.00	3.00	0.21	12.00	0.90	1.00	0.00	

		Chicken wing, with skin, grilled, WO salt	F067	247.00	12.00	9.00	0.35	18.00	0.80	1.60	0.00
		**Chicken composite**		228.67	26.67	8.33	0.28	13.67	1.03	1.60	0.00
**USDA**	Other meat	Pork roast, loin, cooked, lean and fat eaten	1098304	246.00	3.00	6.00	0.70	19.00	0.98	2.30	0.00
		Pork, fresh, shoulder, (Boston butt), blade (steaks), separable lean and fat, cooked, braised	167850	267.00	2.00	0.00	0.93	26.00	1.75	4.84	0.00
		Pork, fresh, loin, center rib (chops), boneless, separable lean and fat, cooked, pan-fried	167892	273.00	2.00	7.00	0.59	11.00	0.73	2.04	0.00
		Pork chop, fried, lean and fat eaten	1098246	211.00	1.00	0.00	0.64	8.00	0.50	1.92	0.00
		Pork chop, stewed, lean and fat eaten	1098255	211.00	1.00	0.00	0.43	9.00	0.63	1.92	0.00
		**Pork composite**		241.60	1.80	2.60	0.66	14.60	0.92	2.60	0.00
**Kenya**	Other meat	Pork, meat, unspecified part, boiled (without salt)	7051	374.00	0.00	4.00	1.00	30.00	1.80	2.45	0.00
		Pork, meat, unspecified part, grilled (without salt and fat)	7052	356.00	0.00	5.00	1.00	26.00	1.30	2.27	0.00
		**Pork composite**		365.00	0.00	4.50	1.00	28.00	1.55	2.36	0.00
**Western Africa**	Other meat	Pork meat, moderately fat, ca. 20% fat, boiled* (without salt), drained	07_057	362.00	6.00	8.00	0.37	12.00	3.10	2.56	0.00
		Pork meat, moderately fat, ca. 20% fat, grilled* (without salt or fat)	07_058	345.00	5.00	9.00	0.51	11.00	2.30	2.97	0.00
		**Pork composite**		353.50	5.50	8.50	0.44	11.50	2.70	2.77	0.00
**Laos**	Other meat	Pork, boiled	6051	204.00	0.00	2.00	0.58	10.00	1.50	1.30	0.00
		Pork, grilled	6016	249.00	0.00	3.00	0.65	43.00	2.50	1.78	0.00
		**Pork composite**		226.50	0.00	2.50	0.62	26.50	2.00	1.54	0.00
**Colombia**	Other meat	Pork, ribs, baked, WO salt	F016	341.00	6.00	0.00	0.77	45.00	1.30	3.30	0.00
		Pork, loin, baked, WO salt	F020	170.00	0.00	0.00	0.56	16.00	1.20	2.50	0.00
		Pork, leg, boiled, WO salt	F024	148.00	0.00	1.00	1.00	17.00	1.10	3.10	0.00

		Pork, picnic shoulder, boiled, WO salt	F007	184.00	2.00	5.00	0.76	14.00	0.90	3.40	0.00	
		**Pork composite**		210.75	2.00	1.50	0.77	23.00	1.13	3.08	0.00	
**USDA**	Organ meats	Beef liver, braised	1098653	189.00	9363.00	251.00	69.99	6.00	6.49	5.26	0.00	
		Beef liver, fried	1098654	174.00	7679.00	258.00	82.44	6.00	6.12	5.19	0.00	
		**Beef liver composite**		181.50	8521.00	254.50	76.22	6.00	6.31	5.23	0.00	
**Kenya**	Organ meats	Beef, liver, boiled (without salt)	7037	178.00	23677.00	264.00	111.00	7.00	14.60	6.25	0.00	
**Western Africa**	Organ meats	Beef liver, boiled* (without salt), drained	07_018	194.00	23600.00	260.00	110.00	40.00	14.10	4.59	0.00	
		Beef liver, grilled* (without salt or fat)	07_088	158.00	21700.00	280.00	100.00	41.00	11.50	3.74	0.00	
		**Beef liver composite**		176.00	22650.00	270.00	105.00	40.50	12.80	4.17	0.00	
**Laos**	Organ meats	Beef, liver, grilled	6023	133.00	3841.00	185.00	51.60	16.00	10.10	3.87	0.00	
		Beef, liver, pan fried		109.06	3779.30	208.40	37.58	13.12	7.87	2.86	0.00	Used weight yields and nutrient retention factors
		**Beef liver composite**		121.03	3810.15	196.70	44.59	14.56	8.99	3.37	0.00	
**Colombia**	Organ meats	Beef, liver, roasted, WO salt	F108	184.00	9491.00	253.00	2.00	7.00	6.50	5.30	0.00	
		Beef, liver, fried, WO salt	F111	169.00	7799.00	260.00	1.00	6.00	7.10	5.20	0.00	
		**Beef liver composite**		176.50	8645.00	256.50	1.50	6.50	6.80	5.25	0.00	
**USDA**	Organ meats	Lamb, variety meats and by-products, liver, cooked, braised	172532	220.00	7491.00	73.00	76.50	8.00	8.28	7.89	0.00	
		Lamb, variety meats and by-products, liver, cooked, pan-fried	172533	238.00	7782.00	400.00	85.70	9.00	10.20	5.63	0.00	
		**Goat/lamb composite**		229.00	7636.50	236.50	81.10	8.50	9.24	6.76	0.00	
**Kenya**	Organ meats	Goat, liver, boiled (without salt)	7045	176.00	19000.00	175.00		14.00	9.40	5.27	0.00	
		Lamb, liver, boiled (without salt)	7048	193.00	38100.00	748.00	89.00	12.00	12.00	6.39	0.00	

		**Goat/lamb composite**		184.50	28550.00	461.50	89.00	13.00	10.70	5.83	0.00	
**Western Africa**	Organ meats	Lamb liver, boiled* (without salt), drained	07_054	218.00	17100.00	200.00	89.00	9.00	14.60	4.53	0.00	
		Lamb liver, grilled* (without salt or fat)	07_131	189.00	15700.00	220.00	83.00	9.00	11.90	3.69	0.00	
		**Goat/lamb composite**		203.50	16400.00	210.00	86.00	9.00	13.25	4.11	0.00	
**USDA**	Organ meats	Chicken liver, braised	1098655	166.00	3948.00	573.00	16.71	11.00	11.53	3.95	0.00	
		Chicken liver, fried	1098656	189.00	2809.00	582.00	15.07	11.00	10.04	3.08	0.00	
		Chicken, liver, all classes, cooked, simmered	171061	167.00	3981.00	578.00	16.85	11.00	11.63	3.98	0.00	
		Chicken, liver, all classes, cooked, pan-fried	174491	172.00	4296.00	560.00	21.13	10.00	12.88	4.01	0.00	
		**Chicken liver composite**		173.50	3758.50	573.25	17.44	10.75	11.52	3.76	0.00	
**Western Africa**	Organ meats	Chicken liver, boiled* (without salt), drained	07_108	173.00	8510.00	1000.00	22.00	7.00	13.60	4.06	0.00	
		Chicken liver, grilled* (without salt or fat)	07_109	141.00	7800.00	1100.00	21.00	7.00	11.10	3.31	0.00	
		**Chicken liver composite**		157.00	8155.00	1050.00	21.50	7.00	12.35	3.69	0.00	
**Laos**	Organ meats	Chicken liver, boiled	6020	121.00	3178.00	462.00	13.50	13.00	7.30	3.18	0.00	
		Chicken liver, grilled	6025	121.00	3273.00	427.00	16.10	13.00	7.30	3.06	0.00	
		**Chicken liver composite**		121.00	3225.50	444.50	14.80	13.00	7.30	3.12	0.00	
**Colombia**	Organ meats	Chicken liver, boiled, WO salt	F078	160.00	3990.00	578.00	16.85	11.00	11.60	4.00	0.00	
		Chicken liver, pan fried		89.38	2444.99	409.84	10.20	9.02	6.70	2.16	0.00	Used weight yields and nutrient retention factors
		**Chicken liver composite**		124.69	3217.50	493.92	13.53	10.01	9.15	3.08	0.00	
**USDA**	Organ meats	Pork, fresh, variety meats and by-products, liver, cooked, braised	167863	165.00	5405.00	163.00	18.67	10.00	17.92	6.72	0.00	
**Laos**	Organ meats	Pork, liver, grilled	6024	125.00	4242.00	128.00	14.70	12.00	15.50	5.27	0.00	
		Pork liver, pan fried		102.50	4642.76	142.89	15.50	9.84	12.07	3.88	0.00	Used weight yields and nutrient retention factors

		**Pork liver composite**		113.75	4442.38	135.45	15.10	10.92	13.79	4.58	0.00	
**Colombia**	Meats & their products	Pork liver, pan fried		100.86	4796.26	147.76	15.76	8.20	18.15	4.46	0.00	Used weight yields and nutrient retention factors
**USDA**	Organ meats	Chicken, heart, all classes, cooked, simmered	171059	185.00	8.00	80.00	7.29	19.00	9.03	7.30	0.00	
		Lamb, variety meats and by-products, heart, cooked, braised	172528	185.00	0.00	2.00	11.20	14.00	5.52	3.68	0.00	
		Beef, variety meats and by-products, heart, cooked, simmered	169448	165.00	0.00	5.00	10.80	5.00	6.38	2.87	0.00	
		Pork, fresh, variety meats and by-products, heart, cooked, braised	168268	148.00	7.00	4.00	3.79	7.00	5.83	3.09	0.00	
		**Heart composite**		175.00	3.50	4.50	9.05	10.50	6.11	3.39	0.00	
**Vietnam**	Organ meats	Chicken heart, pan fried		88.92	6.32	47.74	4.26	9.36	4.42	4.37	0.00	Used weight yields and nutrient retention factors
		Pig heart, pan fried		73.32	5.62	2.65	2.22	5.46	4.37	1.86	0.00	Used weight yields and nutrient retention factors
		**Heart composite**		81.12	5.97	25.20	3.24	7.41	4.40	3.12	0.00	
**Colombia**	Organ meats	Lamb heart, pan fried		105.30	0.00	1.33	6.00	4.68	3.19	1.26	0.00	Used weight yields and nutrient retention factors
		Chicken heart, pan fried		117.78	6.32	47.74	4.26	9.36	4.45	4.38	0.00	Used weight yields and nutrient retention factors
		**Heart composite**		111.54	3.16	24.54	5.13	7.02	3.82	2.82	0.00	
**USDA**	Organ meats	Beef, variety meats and by-products, spleen, cooked, braised	170597	145.00	0.00	4.00	5.02	12.00	39.40	2.79	0.00	
		Lamb, variety meats and by-products, spleen, cooked, braised	174365	156.00	0.00	4.00	5.29	13.00	38.70	3.94	0.00	
		Pork, fresh, variety meats and by-products, spleen, cooked, braised	167866	149.00	0.00	4.00	2.76	13.00	22.20	3.54	0.00	
		**Spleen composite**		149.00	0.00	4.00	5.02	13.00	38.70	3.54	0.00	

**Colombia**	Organ meats	Beef spleen, pan fried		79.56	0.00	2.65	3.32	7.80	32.97	1.39	0.00	Used weight yields and nutrient retention factors
**USDA**	Organ meats	Pork, fresh, variety meats and by-products, kidneys, cooked, braised	167860	151.00	78.00	41.00	7.79	13.00	5.29	4.15	0.00	
		Lamb, variety meats and by-products, kidneys, cooked, braised	174355	137.00	137.00	81.00	78.90	18.00	12.40	3.80	0.00	
		Beef, variety meats and by-products, kidneys, cooked, simmered	169450	158.00	0.00	83.00	24.90	19.00	5.80	2.84	0.00	
		**Kidney composite**		151.00	78.00	81.00	24.90	18.00	5.80	3.80	0.00	
**Western Africa**	Organ meats	Beef kidney, boiled* (without salt), drained	07_090	156.00	166.00	83.00	28.00	8.00	6.20	3.53	0.00	
		Beef kidney, grilled* (without salt or fat)	07_091	154.00	184.00	100.00	32.00	9.00	6.10	3.48	0.00	
		**Kidney composite**		155.00	175.00	91.50	30.00	8.50	6.15	3.51	0.00	
**Vietnam**	Organ meats	Pork kidney, pan fried		56.70	94.50	24.99	4.46	5.60	5.32	1.64	0.00	Used weight yields and nutrient retention factors
**Colombia**	Organ meats	Pork kidney, pan fried		62.30	37.17	24.99	4.46	7.00	3.33	1.61	0.00	Used weight yields and nutrient retention factors
		Lamb kidney, pan fried		72.80	59.85	16.66	27.52	9.10	2.73	1.31	0.00	Used weight yields and nutrient retention factors
		Beef kidney, pan fried		60.90	263.97	58.31	14.44	9.10	3.79	1.13	0.00	Used weight yields and nutrient retention factors
		**Kidney composite**		62.30	59.85	24.99	14.44	9.10	3.33	1.31	0.00	

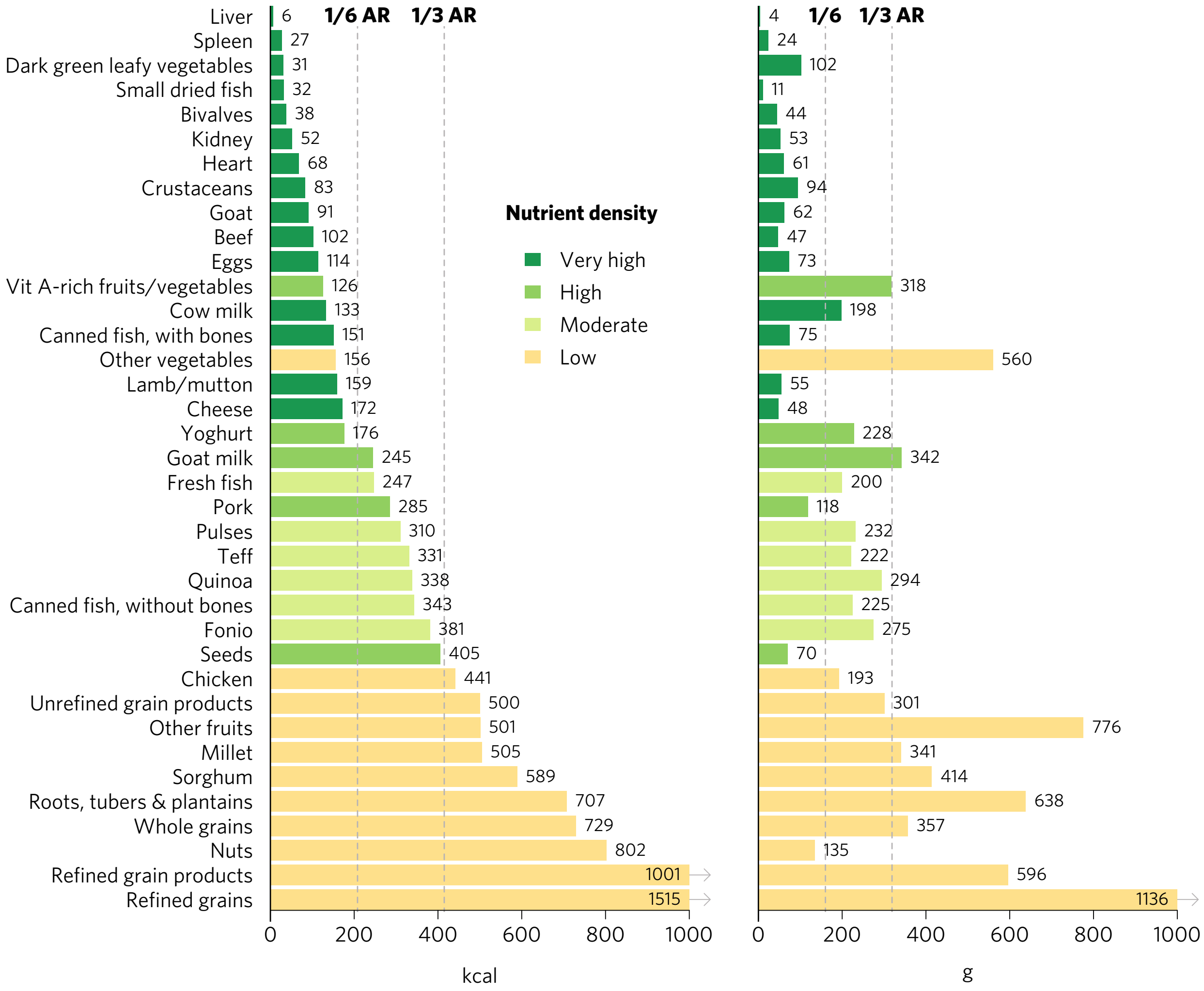

Supplemental Fig. 1 | Calories and grams needed to provide an average of one-third of recommended intakes of vitamin A, folate, vitamin B_{12}, calcium, iron, and zinc for children 2–4 years. Each nutrient's contribution is capped at 100% of recommended intakes. Hypothetical average requirements for mass are based on an energy density of 1.3 kcal/g. AR, average requirement; Vit, vitamin.

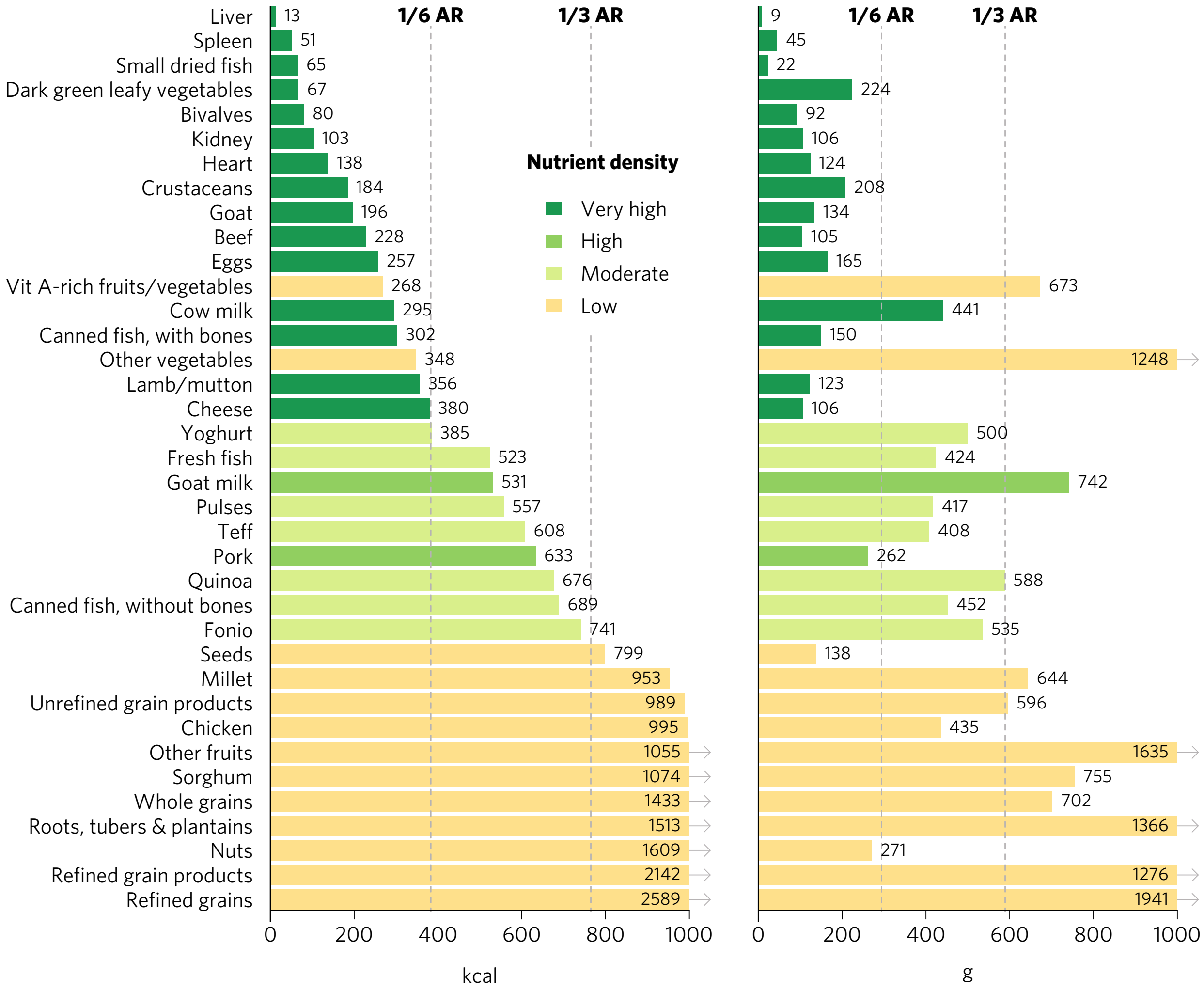

Supplemental Fig. 2 | Calories and grams needed to provide an average of one-third of recommended intakes of vitamin A, folate, vitamin B_{12}, calcium, iron, and zinc for adolescents. Each nutrient's contribution is capped at 100% of recommended intakes. Hypothetical average requirements for mass are based on an energy density of 1.3 kcal/g. AR, average requirement; Vit, vitamin.

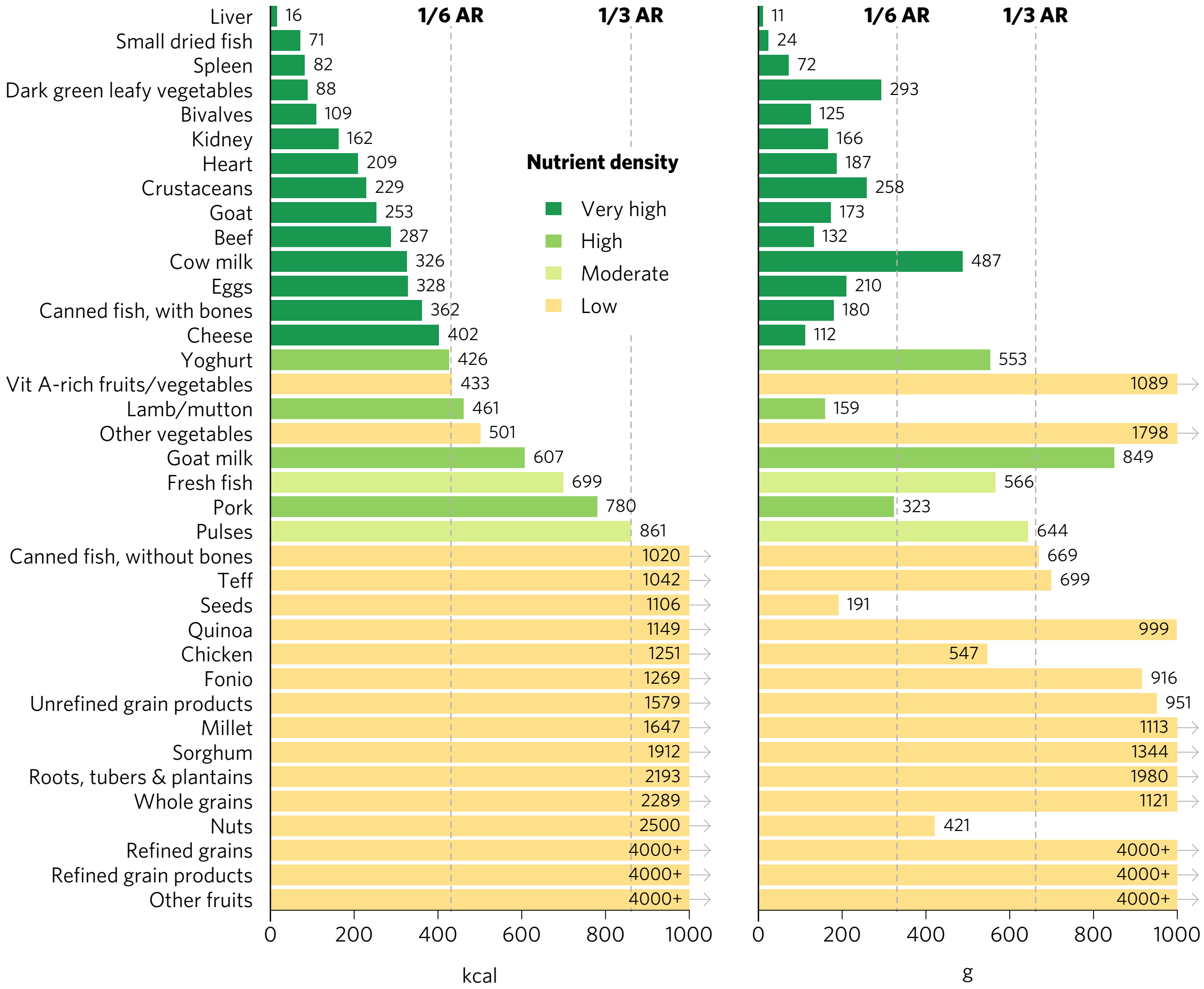

Supplemental Fig. 3 | Calories and grams needed to provide an average of one-third of recommended intakes of vitamin A, folate, vitamin B_{12}, calcium, iron, and zinc for pregnant women. Each nutrient's contribution is capped at 100% of recommended intakes. Hypothetical average requirements for mass are based on an energy density of 1.3 kcal/g. AR, average requirement; Vit, vitamin.

	2+ nutrients	Iron	Zinc	Vitamin A	Calcium	Folate	Vitamin B_{12}
Liver	Very high	Very high	Very high	Very high	Low	Very high	Very high
Spleen	Very high	Very high	Very high	Low	Low	Low	Very high
Dark leafy greens	Very high	High	High	Very high	Very high	Very high	Low
Small dried fish	Very high	Very high	Very high	Very high	Very high	High	Very high
Bivalves	Very high	Very high	Very high	Very high	High	High	Very high
Kidney	Very high	Very high	Very high	Very high	Low	Very high	Very high
Heart	Very high	Very high	Very high	Low	Low	High	Very high
Crustaceans	Very high	Mod	Very high	Low	Mod	Low	Very high
Goat	Very high	Very high	Very high	Low	Low	Low	Very high
Beef	Very high	High	Very high	Low	Low	Low	Very high
Eggs	Very high	Mod	Very high	Very high	Low	Very high	Very high
Cow milk	Very high	Low	Very high	Very high	Very high	Low	Very high
Canned fish w/ bones	Very high	High	Very high	Mod	Very high	Low	Very high
Lamb/mutton	Very high	High	Very high	Low	Low	Low	Very high
Cheese	Very high	Low	Very high	Very high	Very high	Low	Very high
Vit A-rich fruit/veg	High	Low	Low	Very high	Low	High	Low
Yoghurt	High	Low	Mod	Mod	High	Low	Very high
Goat milk	High	Low	High	Very high	Very high	Low	Mod
Pork	High	Low	Very high	Low	Low	Low	Very high
Seeds	High	High	Very high	Low	High	High	Low
Fresh fish	Mod	Low	Very high	Low	Low	Low	Very high
Pulses	Mod	Mod	Very high	Low	Low	Very high	Low
Teff	Mod	Very high	Mod	Low	Low	Very high	Low
Quinoa	Mod	Mod	High	Low	Low	Very high	Low
Canned fish w/o bones	Mod	Mod	Mod	Low	Low	Low	Very high
Fonio	Mod	Mod	Very high	Low	Low	Very high	Low
Other vegetables	Low	Low	Low	Low	Low	High	Low
Chicken	Low	Low	Very high	Low	Low	Low	High
Unrefined grain prod	Low	Mod	High	Low	Low	High	Low
Other fruits	Low	Low	Low	Low	Low	High	Low
Millet	Low	Mod	Mod	Low	Low	Mod	Low
Sorghum	Low	Mod	Mod	Low	Low	Mod	Low
Roots/tubers/plantains	Low	Low	Low	Low	Low	Low	Low
Whole grains	Low	Low	High	Low	Low	Low	Low
Nuts	Low	Low	High	Low	Low	High	Low
Refined grain products	Low	Low	Mod	Low	Low	Low	Low
Refined grains	Low	Low	Mod	Low	Low	Low	Low

Supplemental Fig. 4 | Aggregate and individual micronutrient density scores for children 2–4 years. Mod, Moderate; prod, products; veg, vegetables.

	2+ nutrients	Iron	Zinc	Vitamin A	Calcium	Folate	Vitamin B_{12}
Liver	Very high	Very high	Very high	Very high	Low	Very high	Very high
Spleen	Very high	Very high	Very high	Low	Low	Low	Very high
Small dried fish	Very high	Very high	Very high	Very high	Very high	Low	Very high
Dark leafy greens	Very high	High	Low	Very high	Very high	Very high	Low
Bivalves	Very high	Very high	Very high	Very high	High	Mod	Very high
Kidney	Very high	Very high	Very high	High	Low	Very high	Very high
Heart	Very high	Very high	Very high	Low	Low	Mod	Very high
Crustaceans	Very high	High	Very high	Low	Mod	Low	Very high
Goat	Very high	Very high	Very high	Low	Low	Low	Very high
Beef	Very high	Very high	Very high	Low	Low	Low	Very high
Eggs	Very high	High	High	Very high	Low	Very high	Very high
Cow milk	Very high	Low	High	Very high	Very high	Low	Very high
Canned fish w/ bones	Very high	High	High	Low	Very high	Low	Very high
Lamb/mutton	Very high	High	Very high	Low	Low	Low	Very high
Cheese	Very high	Low	Very high	Very high	Very high	Low	Very high
Goat milk	High	Low	Mod	High	Very high	Low	Mod
Pork	High	High	Very high	Low	Low	Low	Very high
Yoghurt	Mod	Low	Low	Low	High	Low	Very high
Fresh fish	Mod	Mod	Mod	Low	Low	Low	Very high
Pulses	Mod	Very high	Mod	Low	Low	Very high	Low
Teff	Mod	Very high	Mod	Low	Low	Very high	Low
Quinoa	Mod	Mod	Mod	Low	Low	Very high	Low
Canned fish w/o bones	Mod	Mod	Mod	Low	Low	Low	Very high
Fonio	Mod	Very high	Mod	Low	Low	Very high	Low
Vit A-rich fruit/veg	Low	Low	Low	Very high	Low	High	Low
Other vegetables	Low	Low	Low	Low	Low	High	Low
Seeds	Low	High	High	Low	High	High	Low
Millet	Low	Very high	Mod	Low	Low	Mod	Low
Unrefined grain prod	Low	Mod	Mod	Low	Low	Mod	Low
Chicken	Low	Low	High	Low	Low	Low	High
Other fruits	Low	Low	Low	Low	Low	High	Low
Sorghum	Low	Very high	Low	Low	Low	Mod	Low
Whole grains	Low	Mod	Mod	Low	Low	Low	Low
Roots/tubers/plantains	Low	Low	Low	Low	Low	Low	Low
Nuts	Low	Low	Low	Low	Low	Low	Low
Refined grain products	Low	Low	Low	Low	Low	Low	Low
Refined grains	Low	Low	Mod	Low	Low	Low	Low

Supplemental Fig. 5 | Aggregate and individual micronutrient density scores for adolescents. Mod, Moderate; prod, products; veg, vegetables.

	2+ nutrients	Iron	Zinc	Vitamin A	Calcium	Folate	Vitamin B_{12}
Liver	Very high	Very high	Very high	Very high	Low	Very high	Very high
Small dried fish	Very high	Very high	Very high	Very high	Very high	Low	Very high
Spleen	Very high	Very high	Very high	Low	Low	Low	Very high
Dark leafy greens	Very high	Low	Low	Very high	Very high	High	Low
Bivalves	Very high	Very high	Very high	Very high	Very high	Low	Very high
Kidney	Very high	Very high	Very high	High	Low	Mod	Very high
Heart	Very high	Very high	Very high	Low	Low	Low	Very high
Crustaceans	Very high	Low	Very high	Low	High	Low	Very high
Goat	Very high	Very high	Very high	Low	Low	Low	Very high
Beef	Very high	High	Very high	Low	Low	Low	Very high
Eggs	Very high	Low	High	Very high	Low	Mod	Very high
Cow milk	Very high	Low	High	Very high	Very high	Low	Very high
Canned fish w/ bones	Very high	Low	High	Low	Very high	Low	Very high
Cheese	Very high	Low	Very high	Very high	Very high	Low	Very high
Yoghurt	High	Low	Low	Low	Very high	Low	Very high
Lamb/mutton	High	Low	Very high	Low	Low	Low	Very high
Goat milk	High	Low	Mod	High	Very high	Low	Low
Pork	High	Low	Very high	Low	Low	Low	Very high
Fresh fish	Mod	Low	Mod	Low	Low	Low	Very high
Pulses	Mod	Low	Mod	Low	Low	Very high	Low
Vit A-rich fruit/veg	Low	Low	Low	Very high	Low	Low	Low
Other vegetables	Low	Low	Low	Low	Low	Low	Low
Canned fish w/o bones	Low	Low	Mod	Low	Low	Low	Very high
Teff	Low	Mod	Mod	Low	Low	Mod	Low
Seeds	Low	Low	High	Low	High	Low	Low
Quinoa	Low	Low	Mod	Low	Low	Mod	Low
Chicken	Low	Low	High	Low	Low	Low	High
Fonio	Low	Mod	Mod	Low	Low	Mod	Low
Unrefined grain prod	Low	Low	Mod	Low	Low	Low	Low
Millet	Low	Low	Mod	Low	Low	Low	Low
Sorghum	Low	Low	Low	Low	Low	Low	Low
Roots/tubers/plantains	Low	Low	Low	Low	Low	Low	Low
Whole grains	Low	Low	High	Low	Low	Low	Low
Nuts	Low	Low	Low	Low	Low	Low	Low
Refined grains	Low	Low	Mod	Low	Low	Low	Low
Refined grain products	Low	Low	Low	Low	Low	Low	Low
Other fruits	Low	Low	Low	Low	Low	Low	Low

Supplemental Fig. 6 | Aggregate and individual micronutrient density scores for pregnant women. Mod, Moderate; prod, products; veg, vegetables.
